# Promising impact of push–pull configuration into designed octacyclic naphthalene-based organic scaffolds for nonlinear optical amplitudes: a quantum chemical approach

**DOI:** 10.1038/s41598-023-44327-9

**Published:** 2023-11-16

**Authors:** Muhammad Khalid, Iqra Shafiq, Muhammad Adnan Asghar, Ataualpa Albert Carmo Braga, Saad M. Alshehri, Muhammad Haroon, Muhammed Lamin Sanyang

**Affiliations:** 1 Institute of Chemistry, Khwaja Fareed University of Engineering & Information Technology, Rahim Yar Khan, 64200 Pakistan; 2 Centre for Theoretical and Computational Research, Khwaja Fareed University of Engineering & Information Technology, Rahim Yar Khan, 64200 Pakistan; 3 Division of Science and Technology, Department of Chemistry, University of Education, Lahore, Pakistan; 4 Departamento de Química Fundamental, Instituto de Química, Universidade de São Paulo, Av. Prof. Lineu Prestes, 748, São Paulo, 05508-000 Brazil; 5 Department of Chemistry, College of Science, King Saud University, Riyadh, Saudi Arabia; 6 Department of Chemistry and Biochemistry, Miami University, 651 E. High Street, Oxford, OH 45056 USA; 7 University of the Gambia, Kanifing Campus, MDI Road, P.O Box 3530, Kanifing, The Gambia

**Keywords:** Chemistry, Materials science, Optics and photonics

## Abstract

In opto-electronics, non-fullerene (NF) derivatives are regarded as efficient non-linear optical (NLO) materials. The present investigation was based on designing NF naphthalene-based derivatives (PCMD1–D9) with D-π*-*A configuration from PCMR. DFT analysis at M06/6-311G (d,p) level was accomplished to explore the photonic behavior of PCMD1–D9 compounds. Various kind of analysis like; UV–Vis, density of state (DOS), natural bond orbitals (NBOs), transition density matrix (TDM) and frontier molecular orbitals (FMOs) analyses were accomplished to understand the NLO properties of said chromophores. The configuration change led to considerable charge distribution over highest occupied and lowest unoccupied molecular orbitals with minimum band difference. The energy gap trend for all the entitled compounds was observed as; PCMD8 < PCMD5 = PCMD9 < PCMD6 < PCMD7 < PCMD4 < PCMD3 < PCMD2 < PCMD1 with the least band gap of 2.048 eV in PCMD8 among all the compounds. The UV–Visible spectrum of the entitled chromophores manifested high values of *λ*_*max*_ in derivatives contrary to PCMR. Additionally, NBO findings explored effective intramolecular charge transfer and maximum energy of stabilization (34.31 kcal/mol) for PCMD8 chromophore. The highest linear polarizability (<*α*>) and dipole moment (*µ*_*tot*_) values were exhibited by PCMD5 at 2.712 × 10^–22^*.* and 1.995 × 10^–17^ esu, respectively. PCMD8 push–pull configured molecular entity exhibited highest first hyper-polarizability (*β*_*tot*_) at 4.747 × 10^–27^ esu and second hyper-polarizability at 6.867 × 10^–32^ esu. Overall, all the formulated chromophores exhibited significant NLO results contrary to PCMR. Hence, through this structural tailoring via various acceptors, effective NLO materials were obtained for optoelectronic applications.

## Introduction

In the past few decades, non-linear optics (NLO) has emerged as a rapidly expanding field of scientific exploration. It delves into the intricate relationship between light interacts with matter, especially when subjected to external electric fields, this phenomenon referred to as ‘nonlinear optical phenomena’ due to the non-trivial relationship between the response of matter and the strength of the applied electric field^1^. One particularly fascinating aspect of NLO is Second Harmonic Generation (SHG), a process that transforms incoming light waves into waves with double their initial frequency. This phenomenon has garnered extensive attention for its practical applications, particularly in advanced technologies like photovoltaics and optoelectronics^2^.

When an electric field interacts with dielectric materials, it induces a rearrangement of the spatial distribution of electrons around the nucleus. This distortion leads to the establishment of electric dipoles within the material, resulting from these electron–nucleus distortions. The choice of a suitable crystal for a specific application in nonlinear optics depends on several factors, including the nonlinear phenomenon being employed, the characteristics of the pump laser, and the desired properties of the device^3^. Each material possesses unique attributes that may make it well-suited for one application while less relevant for others. The performance of materials becomes notably significant when they exhibit a high degree of nonlinearity, demonstrates promising potential for crystal growth, and possesses a high damage threshold, among other qualities. The continuous development of novel materials with exceptional characteristics plays a pivotal role in advancing leading-edge technologies^4–6^. The strength of optical parallelism and fast speed will progressively generate optoelectronic systems where more functions could be executed optically. Nevertheless, the technological evolution of photonics dependent on formulating novel compounds with enhanced performance^7^.

Different NLO substances have been attained by many scientific efforts during current years to bring out synthetic resins, molecular dyes, organic and inorganic semiconductor diodes. Low dielectric constants, low cost, high photoelectric coefficients, accessibility, the contribution of π-bonding system and electronic displacement besides facile formulation made the organic compounds should be selected in preferences. Intra-molecular charge transfer (ICT) is an important phenomenon in NLO response. The NLO substances demonstrating “push–pull” system due to donor-π-acceptor framework is the reason for development of ICT^8^. Due to D–π–A architecture increased conjugation and these are utilized in number of fields causing organic compound having excellent NLO properties^9^. Conjugated polymers are considered the most comprehensive researched materials for nonlinear optics and among the organics. Having a quality of existence of a delocalized π-electron system making it quick response giving and substantial third-order nonlinear optical characteristics^10,11^. They can also be created in many geometries, such as waveguides, films, fibers, and single crystals and they can be utilized by molecular engineering. So, polymers with the π-conjugated structures are considered as top applicants for succeeding optical photonic technologies.

Due to electronic delocalization, particularly conjugation present in organic materials, they exhibit distinctive optoelectronic characteristics such as photocatalytic, photovoltaic and photoconductive behavior. High second order nonlinear optical response is present in organic compounds demonstrating intramolecular charge transfer (ICT). Charge spread asymmetrically in the p electron structure in organic materials having electron deficient (acceptor) and rich (donor) motifs and hence exhibit enhanced NLO behavior. The conditions of chemical and mechanical stability, large damage threshold, and high phase matchable NLO coefficient must be present in NLO crystals. Molecular properties based on a few of the above conditions can be fulfilled through molecular formulation. By choosing appropriate acceptor and donor combinations, the molecular ICT can be regulated. Various approaches have been presented to develop organic compounds with non-centro symmetric structures like hydrogen bonding^12,13^, reduced ground state dipole interaction, chirality^14,15^, and organometallic complex^16–18^, however physical and chemical methods have been successful in attaining non-centro symmetric organic compounds.

A large and delocalized π-electron system of octacyclic naphthalene-based organic compounds which leads to exceptional hyperpolarizabilities (*β* values) and high nonlinear optical coefficients. Due to their expanded aromatic rings in structure, they have an extremely conjugated π-electron system. This conjugation gives rise to efficient electron delocalization and charge transfer, which are important for generating nonlinear optical responses. Having highly linear planar structure and having strong molecular packing makes the octacyclic naphthalene-based organic scaffolds (NITT) selection as π-spacer for current study. Yang et al. inserted a terminal end group i.e., IC-2F with NITT core, namely NITTBF to further explore nonlinear properties^19^. NITT-BF compound showed red shifted absorption along with higher electron mobility because of enhanced intermolecular π–π stacking. Its blend film gives increased exciton dissociation and charge collection properties. NITT-BF delivers higher electron mobility and stronger intermolecular π–π stacking, which account for the higher exciton dissociation and charge collection efficiency in NITT-BF-based device. In addition, NITT-BF has an optical energy gap of 1.25 eV which corresponds to higher nonlinearity.

Therefore, in present investigation we have formulated novel donor–π–acceptor (D–π–A) configured PCMD1–D9 organic chromophores by substituting acceptor motif in the reference PCMR having acceptor–π–acceptor (A–π–A) configuration from parent NITT-BF. The reference compound has been obtained by substituting R1 (3-ethylheptane) and R2 (1-hexyl-4-methylbenzene) with methyl group to reduce computational cost and time of investigation. However, in PCMD9 the one side acceptor is replaced with donor motif which remained constant in the rest of the compounds. But from PCMD1–D9 the end capped acceptor group was substituted by variant acceptor groups to study their molecular nonlinearity and impact on ICT. The analyses were performed at M06/6-311G(d,p) functional. The TD-DFT and DFT calculations would be executed to interpret the effect of variant acceptors on intramolecular charge transmission, band gap, nonlinear response and absorption spectra. Therefore, various analysis such as FMO, NLO, NBO, UV–Vis, TDM and DOS were performed. We hope these theoretically engineered molecules will lead to more advancements in leading optical technology.

### Computational procedure

The FMO, NLO, NBO and absorption spectra of naphthalene-based reference and derivatives (PCMR with acceptor-π-acceptor (A–π–A) and PCMD1–D9 having donor-π-acceptor (D–π–A) system) were calculated employing density functional theory (DFT) via Gaussian 09 program^20^. Complete investigation of the present research were accomplished utilizing M06^21^/6-311G(d,p)^22^ theory level. Transition density matrix (TDM) as well as natural bond orbital (NBO) analyzed charge transition interactions through Multiwfn 3.7^23^ and NBO package 3.1^24^. The energies of frontier molecular orbitals (FMOs) and orbital diagrams were obtained through Avogadro^25^. The Ultraviolet–Visible (UV–Vis) study was executed utilizing GaussSum^26^ and the spectral diagram was depicted via Origin 8.0^27^. However, the density of states illustrations were obtained through PyMOlyze^28^. The dipole moment (*µ*_*tot*_)^29^ and nonlinear optical parameters like linear polarizability (<*α*>)^30^ as well as nonlinear hyperpolarizability (*β*_*tot*_ and *γ*_*tot*_)^31^ were also computed at the same level M06/6-311G(d,p) through Eqs. (1)–(4).

1 $$ \mu = \, \left( {\mu^{{2}}_{x} + \mu^{2}_{y} + \mu^{{2}}_{z} } \right)^{{{1}/{2}}} , $$

2 $$ \left\langle \alpha \right\rangle = {1}/{3}(\alpha_{{{\text{xx}}}} + \alpha_{{{\text{yy}}}} + \alpha_{{{\text{zz}}}} ), $$

3 $$ \beta_{tot} = [\beta_{{\text{x}}}^{{2}} + \beta_{{\text{y}}}^{{2}} + \beta_{{\text{z}}}^{{2}} ]^{{{1}/{2}}} , $$

where *β*_*x*_ = *β*_*xxx*+_*β*_*xyy*+_*β*_*xzz*_, *β*_*y*_ = *β*_*yxx*+_*β*_*yyy*+_*β*_*yzz*_ and *β*_*z*_ = *β*_*zxx*+_*β*_*zyy*+_*β*_*zzz*_

4 $${\gamma }_{tot}=\sqrt{{\gamma }_{x }^{2}+ {\gamma }_{y}^{2}+{\gamma }_{z}^{2}},$$

where $${\gamma }_{i}= \frac{1}{15 }\sum_{j}({\gamma }_{ijji}+{\gamma }_{ijij}+{\gamma }_{iijj}) \quad i,j = \{x, y, z\}$$.

Equations (5)–(11) were employed to calculate global reactivity descriptors i.e. global softness (*σ*), chemical potential (*μ*)^32^, ionization potential (*IP*)^33^, electronegativity (*X*)^34^, global hardness (*η*)^35^, electron affinity (*EA*) and global electrophilicity index (*ω*)^36^.

5 $$ IP = - E_{{{\text{HOMO}}}} , $$

6 $$ EA = - E_{{{\text{LUMO}}}} , $$

7 $$X=\frac{\left[IP+EA\right]}{2},$$

8 $$\eta =\frac{\left[IP-EA\right]}{2},$$

9 $$\mu =\frac{{E}_{\mathrm{HOMO}}{+E}_{\mathrm{LUMO}}}{2},$$

10 $$\sigma =\frac{1}{2\eta },$$

11 $$\omega =\frac{{\mu }^{2}}{2\eta }.$$

## Results and discussion

**Figure 1:**
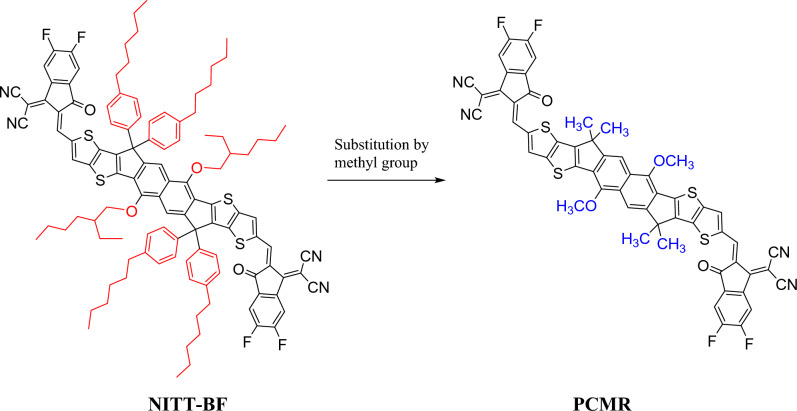
Synthesized chromophore NITT-BF modification into reference PCMR by substitution of methyl group. These structures are drawn with the help of ChemDraw software (https://chemistrydocs.com/chemdraw-pro-8-0/ ).

**Figure 2:**
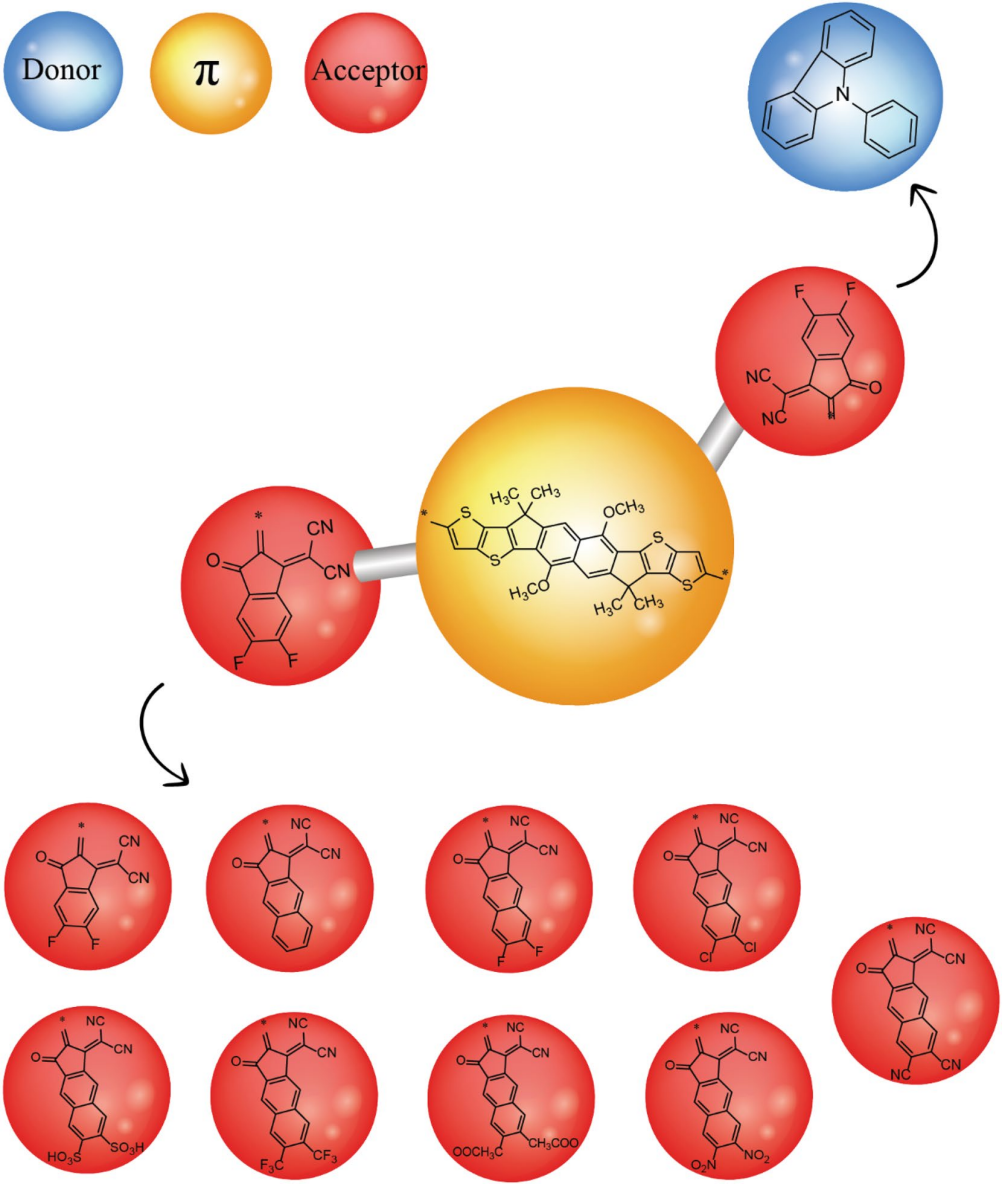
Schematic demonstration of D–π–A configured PCMR and PCMD1–D9 chromophores with variant acceptors. This scheme is drawn with the help of ChemDraw software (https://chemistrydocs.com/chemdraw-pro-8-0/).

The present research present exploration of new organic chromophores with high nonlinearity. For this purpose, already synthesized organic compound NITT-BF^19^ was utilized. The structural modification of NITT-BF into three parts; 2-(5,6-difluoro-2-methylene-3-oxo-2,3-dihydro-1H-inden-1-ylidene) malononitrile two acceptor groups on either side and NITT core. The R1 (3-ethylheptane) and R2 (1-hexyl-4-methylbenzene) groups in NITT core were replaced by methyl groups to convert NITT-BF to reference PCMR with A–π–A configuration (Fig. 1). We have designed a series of derivatives (PCMD1–D9) by substituting first A in PCMR with 9-phenyl-9H-carbazole donor group and varying the second acceptor group with 2-(5,6-difluoro-2-methylene-3-oxo-2,3-dihydro-1H-inden-1-ylidene)malononitrile in PCMD1, 2-(2-methylene-3-oxo-2,3-dihydro-1H-cyclopenta[b]naphthalen-1-ylidene)malononitrile in PCMD2, 2-(6,7-difluoro-2-methylene-3-oxo-2,3-dihydro-1H-cyclopenta[b]naphthalen-1-ylidene)malononitrile in PCMD3, 2-(6,7-dichloro-2-methylene-3-oxo-2,3-dihydro-1H-cyclopenta[b]naphthalen-1-ylidene)malononitrile in PCMD4, 1-(dicyanomethylene)-2-methylene-3-oxo-2,3-dihydro-1H-cyclopenta[b]naphthalene-6,7-disulfonic acid in PCMD5, 2-(2-methylene-3-oxo-6,7-bis(trifluoromethyl)-2,3-dihydro-1H-cyclopenta[b]naphthalen-1-ylidene)malononitrile in PCMD6, 2-(6,7-dimethyl-2-methylene-3-oxo-2,3-dihydro-1H-cyclopenta[b]naphthalen-1-ylidene)malononitrile-carbon(IV) oxide (1/2) in PCMD7, 2-(2-methylene-6,7-dinitro-3-oxo-2,3-dihydro-1H-cyclopenta[b]naphthalen-1-ylidene)malononitrile in PCMD8 and 1-(dicyanomethylene)-2-methylene-3-oxo-2,3-dihydro-1H-cyclopenta[b]naphthalene-6,7-dicarbonitrile in PCMD9, where the scheme is shown in Fig. 2. The ChemDraw structures of reference and derivatives are presented in Fig. S1. However, the optimized structures are presented in Fig. S2 while their cartesian coordinates are illustrated in Tables S1–S10. The TD-DFT and DFT calculations are executed to interpret the effect of variant acceptors on intramolecular charge transmission, band gap, nonlinear response and absorption spectra. To deduce the impact of different acceptors on ICT, nonlinearity, absorption spectra and band gap, DFT computations were performed.

### Electronic structures

NLO characteristics of molecule’s electronic structure is determine by frontier molecular investigation^37^. Light absorption molecular capability, electronic properties and chemical stability are comprehended via FMO analysis^30,38,39^. Highest occupied molecular orbital/lowest unoccupied molecular orbital (HOMO/LUMO) energy gap is directly influenced by above mentioned factors. These orbitals determine intra-molecular charge transference efficiency^40–42^. However, their energy difference (Δ*E*) is efficient to know molecular chemical reactivity as well as dynamic stability Less Δ*E* value corresponds to high polarizability which in turn lead to exceptional NLO behavior^43^. On the other hand, high Δ*E* value corresponds to molecular stability and hardness leading to less reactivity and chemical alteration. Table 1 displays energy difference along with energy values of HOMO and LUMO for the designed organic compounds.

**Table 1:** Energy difference between highest occupied and lowest unoccupied molecular orbitals along with their energy values for PCMR and PCMD1–D9 (in eV).

Compounds	*E*_*HOMO*_	*E*_*LUMO*_	Δ*E*
PCMR	− 5.941	− 3.505	2.436
PCMD1	− 5.658	− 3.362	2.296
PCMD2	− 5.637	− 3.352	2.285
PCMD3	− 5.649	− 3.402	2.247
PCMD4	− 5.657	− 3.445	2.212
PCMD5	− 5.697	− 3.623	2.074
PCMD6	− 5.682	− 3.525	2.157
PCMD7	− 5.659	− 3.459	2.200
PCMD8	− 5.696	− 3.648	2.048
PCMD9	− 5.693	− 3.619	2.074

From the above table, the reference organic molecule (PCMR) possesses − 5.941 and − 3.505 eV of HOMO and LUMO energies, which are comparable to the experimental values of − 5.75 and − 4.15 eV, respectively, indicating the accurateness in the selection of functional group for the present analysis. The achieved values of HOMO for all the derivatives (PCMD1–D9) are higher than the reference compound at − 5.658, − 5.637, − 5.649, − 5.657, − 5.697, − 5.682, − 5.659, − 5.696 and − 5.693 eV, respectively. However, the obtained LUMO values for PCMD1–D4 and PCMD7 are higher than the reference i.e., − 3.362, − 3.352, − 3.402, − 3.445 and − 3.459 eV, correspondingly but on the other hand PCMD5, PCMD6, PCMD8 and PCMD9 exhibit less values than PCMR at − 3.623, − 3.525, 3.648 and − 3.619 eV, respectively. Hence, the high HOMO and less LUMO values result in less band gap in PCMD5, PCMD6, PCMD8 and PCMD9 showing high charge transmission probability in these molecules.

The highest band gap is exhibited by PCMR at 2.436 eV. This largest energy difference is attributed to the presence of 2-(5,6-difluoro-3-oxo-2,3-dihydro-1H-inden-1-ylidene)malononitrile as a acceptor motifs with A–π–A configuration. Because moving from PCMR to PCMD1 the one end capped acceptor group is substituted with 9-phenyl-9H-carbazole which significantly reduces the band gap to 2.296 eV in PCMD1.

**Figure 3:**
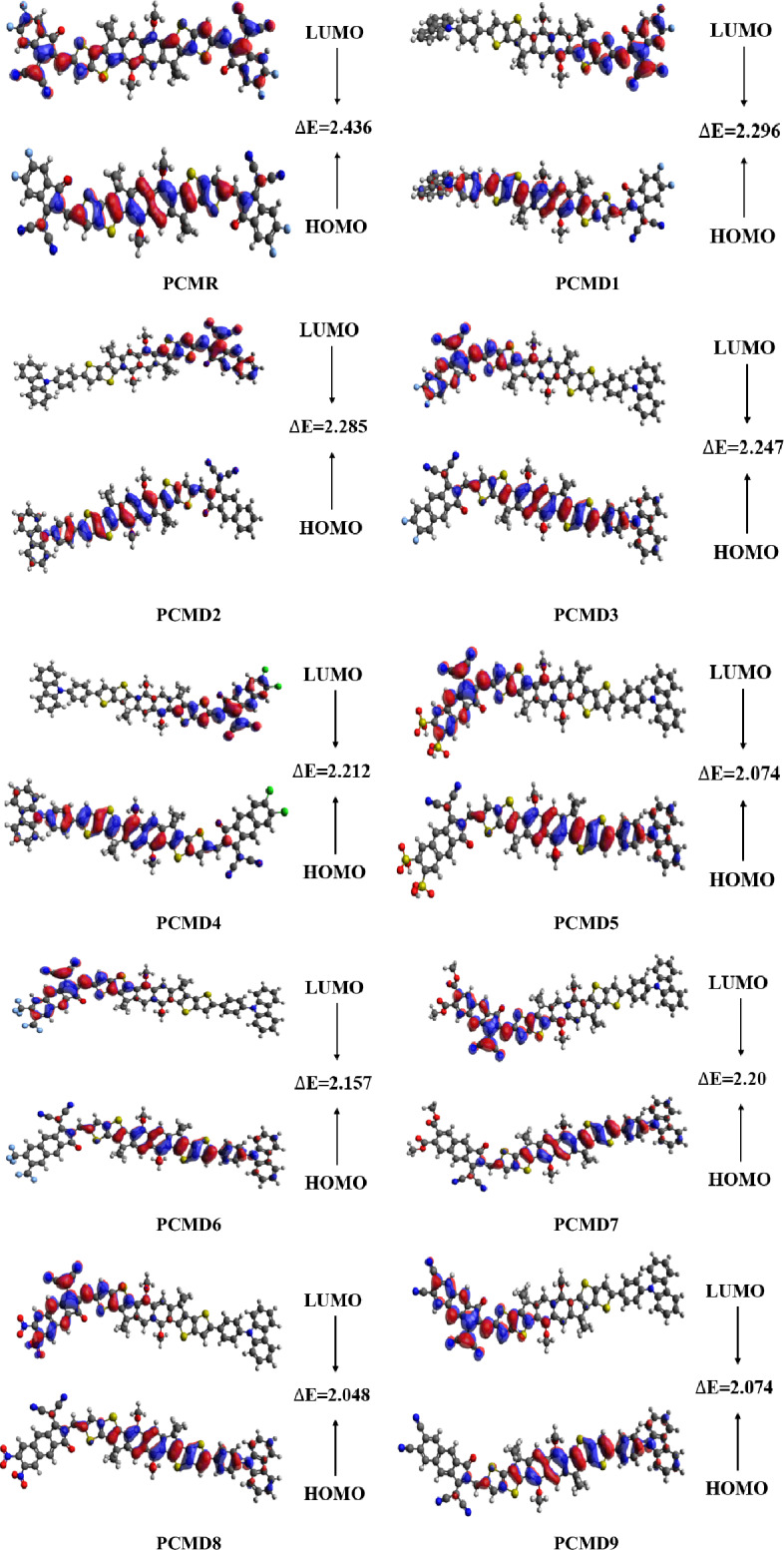
The excited and ground states (HOMO–LUMO) Δ*E* values for PCMR and PCMD1–D9 at M06/6-311G(d,p) and output files were calculated through Gaussian 09 version D.01.

Conversely, the least band gap value of 2.048 eV is present in PCMD8, that might be owing to the presence of high electronegative nitro functional groups at 6 and 7 positioning of 1-(dicyanomethylene)-3-oxo-2,3-dihydro-1H-cyclopenta[b]naphthalene (DMP) acceptor group. The second lowest energy gap value is exhibited by PCMD5 and PCMD9 at 2.074 eV because of the presence of sulfonic acid groups and cyano groups at 6 and 7 positioning of DMP acceptor moiety rendering high electron withdrawing tendency. However, PCMD6 possess 2.157 eV energy gap resulted from the 6, 7 positioning of trifluoromethyl functional group in DMP. The presence of acetate, chloro and fluoro groups at 6, 7 positioning of DMP acceptor group in PCMD7, PCMD4 and PCMD3 corresponds to band gap of 2.200, 2.212 and 2.247 eV, respectively. 2.285 eV of energy gap is present in PCMD2 in relation to the absence of any electron withdrawing functional groups in DMP. The ascending order of energy difference (HOMO/LUMO) for the derivatives is PCMD8 < PCMD5 = PCMD9 < PCMD6 < PCMD7 < PCMD4 < PCMD3 < PCMD2 < PCMD1. Figure 3 presents MO surfaces of PCMR and PCMD1–D9 showing complete electronic transmission from HOMO of donor and π-spacer groups towards acceptor. Concluding our discussion, the less energy difference as exhibited by formulated derivatives, which supported the grater absorption properties that indicates their high efficiency in modern optical devices.

### Density of states (DOS)

**Figure 4:**
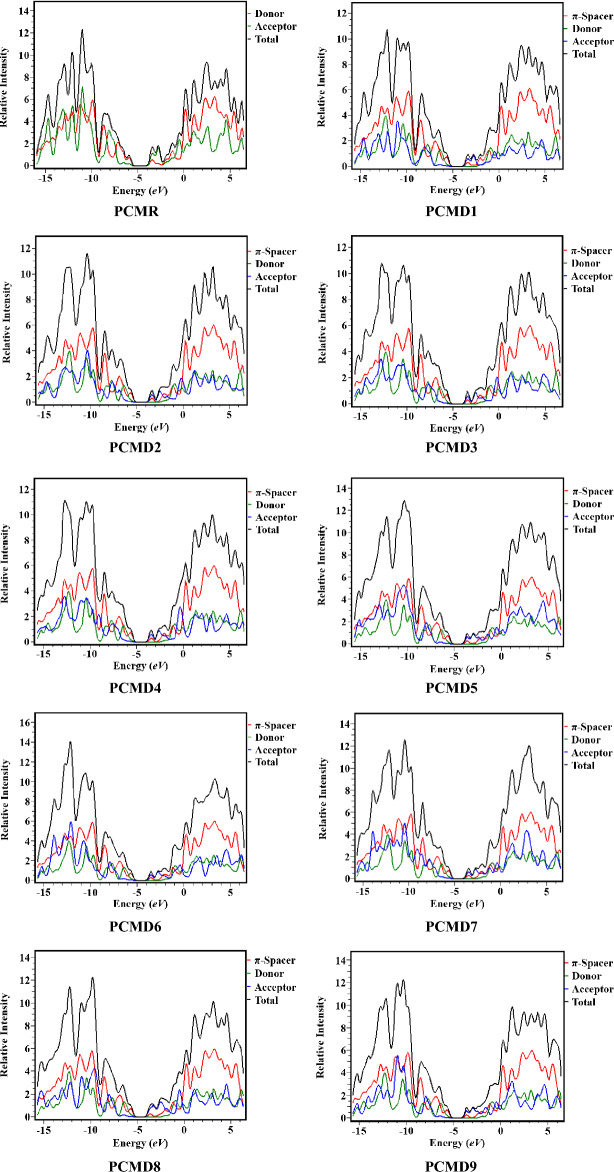
Density of states diagrams of PCMR and PCMD1–D9 at M06/6-311G(d,p) level. Figures were drawn by utilizing PyMOlyze 1.1 version and output files were calculated through Gaussian 09 version D.01.

The DOS analysis verify the results shown by the FMOs diagrams and explain the electronic distribution in frontier molecular orbitals^44^. In DOS pictographs, the HOMO signifies valence band showing negative values while the positive values are represented by the conduction band (LUMO)^45^. The analysis was performed at the same DFT functional for PCMR and PCMD1–D9. DOS indicates the contribution of each fragment of molecule in charge transfer. For determining the density of states (DOS), we split our studied molecules into se98parate fragments. The PCMR1 was divided into two fragments i.e*.,* donor (D) and acceptor (A) while, the derivatives (PCMD1–D9) were divided into three segments i.e*.,* donor, π-spacer, and acceptor. From Fig. 4, it is demonstrated that in PCMR molecule HOMO density appear on donor unit while LUMO density is majorly present on acceptor units. In the designed molecules PCMD1–D9, distribution pattern of HOMO and LUMO density is same as HOMO density is majorly placed on π-spacer while minor HOMO density is situated on donor atoms. LUMO density in all the designed molecules is majorly present on acceptor units whereas minor amount is spread on donor atoms. In LUMO, the donor core percentage contribution for reference PCMR and designed molecules PCMD1–D9 are noted as 38.2, 0.2, 0.2, 0.2, 0.2, 0.1, 0.2, 0.2, 0.1 and 0.2% respectively which are correlated with FMOs surfaces. Similarly, for LUMO the percentage contribution of acceptors in PCMR and PCMD1–D9 have been found as 61.8, 64.9, 64.3, 64.7, 65.4, 68.1, 65.9, 65.9, 70.2 and 68.0%, respectively. Likewise, for π-spacer, the percentage contribution is examined as 34.9, 35.5, 35.1, 34.5, 31.7, 34.0, 33.9, 29.6 and 31.8%, respectively in PCMD1–D9. In the same manner, in HOMO the donor contributions are studied as 80.3, 16.2, 15.6, 16.4, 16.8, 18.8, 16.8, 17.1, 19.2 and 18.8%, for PCMR and PCMD1–D9 chromophores, respectively. On the other hand, for acceptor 19.7, 5.3, 5.9, 5.7, 5.6, 5.2, 5.5, 5.6, 5.2 and 5.2% contributions are investigated in HOMO for PCMR and PCMD1–D9, correspondingly. HOMO percentage contribution for designed molecules PCMD1–D9 In the same way, for HOMO, π-spacer contributed as 78.5, 78.5, 77.9, 77.5, 76.0, 77.6, 77.3, 75.7, and 75.9% respectively in PCMD1–D9 chromophores. The percentages of electronic cloud distribution molecular orbitals is same as illustrated in FMOs surfaces. The overall contribution pattern has shown a significant electronic charge variation in molecular systems, which depicts that a considerable amount of charge transfer takes place from the donor to acceptor region via the π-spacer.

### Absorption spectrum

**Figure 5:**
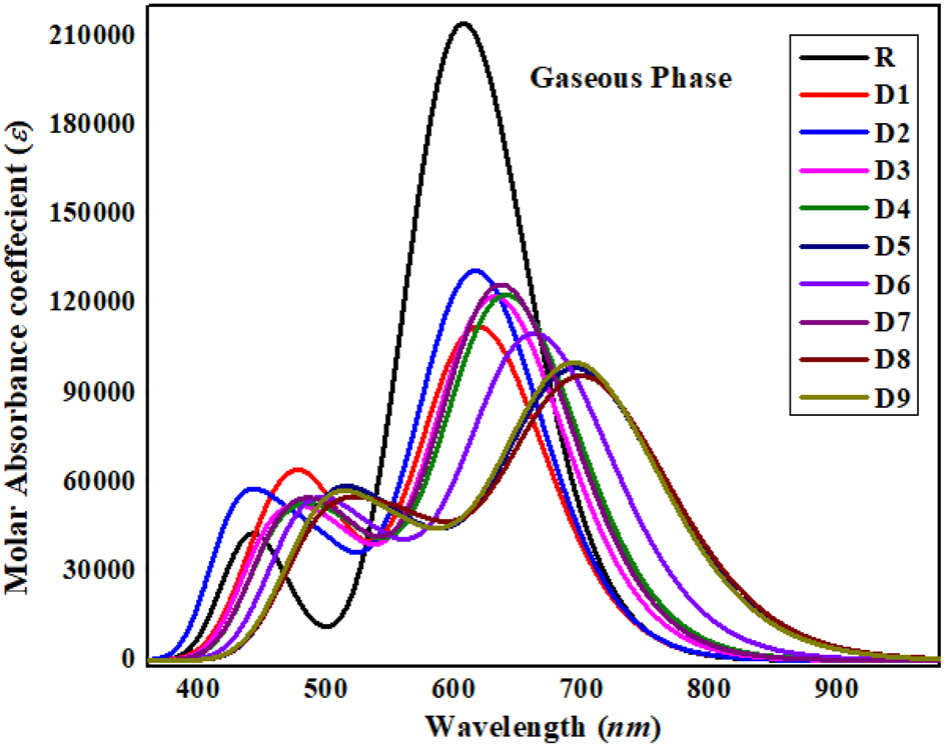
PCMR and PCMD1–D9 computed ultraviolet–visible spectrum in gas medium illustrated by Origin Pro 8.5.

The ultraviolet visible computation in gaseous form for PCMR and PCMD1–D9 were performed at M06/6-311G(d,p) to apprehend oscillator strength as well as excitation energy with relation to important electronic transitions^46,47^. Franck–Condon principle relates spectral highest absorption maxima (*λ*_*max*_) to vertical excitation. The information about oscillator strength (*f*_os_), absorption maxima (*λ*_*max*_), excitation energy (*E*), along with molecular orbital (MO) contribution in transition are given in Table S12 (rest of data is given in Table S11). The spectral representation of absorption maxima (*λ*_*max*_) in ultraviolet visible region for PCMR and PCMD1–D9 is presented in Fig. 5.

From the major findings presented in Table S12, the absorption maxima (*λ*_*max*_) value for reference (PCMR) i.e., 606.899 nm is lowest among all the compounds with transition energy of 2.043 eV, 2.956 oscillator strength and molecular orbital contribution from HOMO to LUMO of 95%, however, from HOMO-1 to LUMO-1 it is 3%. Hence, it is observed that absorption maxima are significantly impacted by highly electron rich donor and electron withdrawing acceptor end capped groups as well as molecular configuration (reference (A–π–A)$\to$ derivatives (D–π–A)) creating strong push pull mechanism. The least *λ*_*max*_ value of reference compound resulted from two electron withdrawing acceptor groups on either side of the π-linker. However, the highest *λ*_*max*_ value of 701.541 nm is exhibited by PCMD8 among all the chromophores because of high electronegative nitro functional groups present in DMP acceptor group, results in 1.767 eV energy of transition and oscillator strength of 1.299. The molecular orbital contribution exhibited by PCMD8 compound is 96% from HOMO to LUMO and 3% HOMO-1 to LUMO. The second highest *λ*_*max*_ of 696.849 nm is present in PCMD5 due to the presence of sulfonic acid groups in DMP with 1.779 eV of transition energy, 1.779 *f*_os_ and MO contribution of 95 and 3% from HOMO to LUMO and HOMO-1 to LUMO, respectively. However, the third highest *λ*_*max*_ of 695.442 nm is shown by PCMD9 because of cyano groups presence in DMP with transition energy and *f*_os_ values of 1.783 eV and 1.362, respectively. However, the molecular orbital contributions in PCMD9 corresponds to 95% from HOMO to LUMO and 3% from HOMO-1 to LUMO. Owing to the presence of trifluoromethyl groups in PCMD6, the *λ*_*max*_ reduces to 663.580 nm with E 1.868 eV, 1.504 *f*_os_ and MO contributions of 95 and 3% from HOMO to LUMO and HOMO-1 to LUMO, respectively. Substituting trifluoromethyl groups by chloro groups result in *λ*_*max*_ of 641.238 nm in PCMD4 with E 1.934 eV, 1.687 *f*_os_ and MO contributions of 95 and 3% from HOMO to LUMO and HOMO-1 to LUMO, respectively. The overall decreasing trend for PCMD1–D9 as; PCMD8 > PCMD5 > PCMD9 > PCMD6 > PCMD4 >  > PCMD3 > PCMD1 > PCMD2 with chief molecular orbital contribution is from HOMO to LUMO of 94–96%. The compounds with lower *E*_*g*_ showed wider absorption spectrum as the decreasing trend of *λ*_*max*_ is almost similar with the increasing trend of energy gap. All fabricated molecules showed bathochromic shift (Fig. 5) with reduced band gap than that of reference molecule, particularly, PCMD8. Hence, it is anticipated that these chromophores will be significantly utilized for NLO materials.

### Natural bond orbitals (NBO)

To study charge density migration and hyper conjugation resulting from non-covalent interactions between acceptor and donor groups, natural bond orbitals investigation is employed. Equation (12) was applied to calculate energy of stabilization.

12 $${E}^{\left(2\right)}=\Delta {E}_{ij}={q}_{i}\frac{{\left({F}_{i,j}\right)}^{2}}{\left({E}_{j}-{E}_{i}\right)}.$$

Here, *q*_*i*_ corresponds to donor orbital occupancy, *E*_i_ and *E*_j_ denotes off-diagonal NBO Fock or Kohn–Sham medium elements, whereas the diagonal is characterized by *F*(*i.j*)^48,49^. σ → σ*, π → π*, LP → σ* and LP → π* orbital overlapping lead to hyper conjugation. The π-conjugation present in the computed derivatives having D–π–A configuration lead to π → π* transitions, hence result in efficient nonlinear optical substances. Weak σ → σ* transitions are also present because of poor donor and acceptor interactions. Table 2 presents major transition values of PCMR and PCMD1–D9, however detailed data is present in Tables S13–S22.

**Table 2:** Different transition types along with their values for PCMR and PCMD1–D9.

Compounds	Donor(*i*)	Type	Acceptor(*j*)	Type	*E*(2) [kcal/mol]	*E*(*j*) − *E*(*i*) [a.u.]	F(*i,j*) [a.u.]
PCMR	C18–C19	π	C22–C23	π*	31.57	0.31	0.089
	C61–C62	π	C61–C62	π*	0.65	0.31	0.013
	C22–H24	σ	C18–S20	σ*	10.27	0.71	0.076
	C37–N38	σ	C33–C36	σ*	0.51	1.65	0.026
	C68	LP(1)	C64–C66	π*	72.81	0.17	0.111
	O35	LP(2)	C29–C34	σ*	21.25	0.76	0.115
PCMD1	C18–C19	π	C22–C23	π*	32.39	0.31	0.09
	C34–O35	π	C28–C29	π*	4.16	0.42	0.042
	C22–H24	σ	C18–S20	σ*	10.36	0.71	0.077
	C65–C66	σ	C6–C15	σ*	0.5	1.24	0.022
	N82	LP(1)	C83–C85	π*	35.44	0.31	0.096
	O35	LP(2)	C29–C34	σ*	21.3	0.76	0.115
PCMD2	C18–C19	π	C22–C23	π*	31.82	0.31	0.089
	C107–C108	π	C107–C108	π*	0.67	0.29	0.013
	C22–H24	σ	C18–S20	σ*	10.42	0.71	0.077
	C62–C64	σ	C62–S63	σ*	0.5	0.91	0.019
	N96	LP(1)	C107–C108	π*	35.42	0.31	0.096
	O35	LP(2)	C29–C34	σ*	20.67	0.77	0.114
PCMD3	C18–C19	π	C22–C23	π*	32.24	0.31	0.09
	C80–C83	π	C80–C83	π*	0.51	0.32	0.012
	C22–H24	σ	C18–S20	σ*	10.43	0.71	0.077
	C61–C62	σ	C6–C59	σ*	0.5	1.24	0.022
	S20	LP(2)	C12–C13	π*	29.54	0.24	0.079
	O35	LP(2)	C29–C34	σ*	20.8	0.76	0.114
PCMD4	C18–C19	π	C22–C23	π*	32.6	0.31	0.09
	C105–C106	π	C105–C106	π*	0.67	0.29	0.013
	C22–H24	σ	C18–S20	σ*	10.48	0.71	0.077
	C61–C62	σ	C6–C59	σ*	0.5	1.24	0.022
	S20	LP(2)	C12–C13	π*	29.56	0.24	0.079
	O35	LP(2)	C23–C34	σ*	18.57	0.76	0.107
PCMD5	C18–C19	π	C22–C23	π*	34.13	0.31	0.092
	C39–N40	π	C37–N38	π*	0.8	0.47	0.017
	C12–C13	σ	C13–C19	σ*	4.92	1.3	0.072
	C62–C64	σ	C62–S63	σ*	0.5	0.92	0.019
	C3	LP(1)	C9–C57	π*	69.77	0.15	0.11
	O119	LP(3)	S118–O122	σ*	29.05	0.45	0.103
PCMD6	C18–C19	π	C22–C23	π*	33.46	0.31	0.091
	C95–C96	π	C95–C96	π*	0.67	0.29	0.013
	C3–C4	σ	C4–C5	σ*	4.99	1.33	0.073
	C62–C64	σ	C62–S63	σ*	0.5	0.92	0.019
	N94	LP(1)	C95–C96	π*	35.34	0.31	0.096
	O35	LP(2)	C29–C34	σ*	21.11	0.76	0.114
PCMD7	C18–C19	π	C22–C23	π*	32.84	0.31	0.091
	C95–C96	π	C95–C96	π*	0.67	0.29	0.013
	C22–H24	σ	C18–S20	σ*	10.54	0.71	0.077
	C61–C62	σ	C6–C59	σ*	0.5	1.24	0.022
	O120	LP(2)	C118–O119	π*	47.48	0.39	0.122
	O116	LP(2)	C115–O117	σ*	33.38	0.67	0.135
PCMD8	C18–C19	π	C22–C23	π*	34.31	0.31	0.092
	C39–N40	π	C37–N38	π*	0.81	0.47	0.017
	C3–C4	σ	C4–C5	σ*	4.99	1.33	0.073
	C37–N38	σ	C33–C36	σ*	0.5	1.65	0.022
	O117	LP(3)	N115–O116	π*	194.76	0.17	0.162
	O35	LP(2)	C29–C34	σ*	21.35	0.75	0.115
PCMD9	C18–C19	π	C22–C23	π*	34.06	0.31	0.092
	C37–N38	π	C39–N40	π*	0.79	0.47	0.017
	C62–C64	σ	C66–C84	σ*	4.99	1.22	0.07
	C61–C62	σ	C6–C59	σ*	0.5	1.24	0.022
	N94	LP(1)	C105–C106	π*	35.4	0.31	0.096
	O35	LP(2)	C29–C34	σ*	21.24	0.76	0.115

Molecular orbitals for PCMR and PCMD1–D9 involve in consistent π → π* transitions are π(C18–C19) → π*(C22–C23) corresponds to stabilization energies of 31.57, 32.39, 31.82, 32.24, 32.6, 34.13, 33.46, 32.84, 34.31 and 34.06 kcal/mol, respectively. The lowest π → π* transitions exhibited by PCMR and PCMD1–D9 corresponds to π(C61–C62) → π*(C61–C62), π(C34–O35) → π*(C28–C29), π(C107–C108) → π*(C107–C108), π(C80–C83) → π*(C80–C83), π(C105–C106) → π*(C105–C106), π(C39–N40) → π*(C37–N38), π(C95–C96) → π*(C95–C96), π(C95–C96) → π*(C95–C96), π(C39–N40) → π*(C37–N38) and π(C37–N38) → π*(C39–N40) at 0.65, 4.16, 0.67, 0.51, 0.67, 0.8, 0.67, 0.67, 0.81 and 0.79 kcal/mol stabilization energy, respectively.

Likewise, orbitals involved in σ → σ* transitions in PCMR and PCMD1–D9 are σ(C22–H24) → σ*(C18–S20), σ(C22–H24) → σ*(C18–S20), σ(C22–H24) → σ*(C18–S20), σ(C22–H24) → σ*(C18–S20), σ(C22–H24) → σ*(C18–S20), σ(C12–C13) → σ*(C13–C19), σ(C3–C4) → σ*(C4–C5), σ(C22–H24) → σ*(C18–S20), σ(C3–C4) → σ*(C4–C5) and σ(C62–C64) → σ*(C66–C84) at highest energy of stabilization of 10.27, 10.36, 10.42, 10.43, 10.48, 4.92, 4.99, 10.54, 4.99 and 4.99 kcal/mol, respectively. Whereas, the lowest stabilization energy for σ → σ* transitions involve σ(C37–N38) → σ*(C33–C36), σ(C65–C66) → σ*(C6–C15), σ(C62–C64) → σ*(C62–S63), σ(C61–C62) → σ*(C6–C59), σ(C61–C62) → σ*(C6–C59), σ(C62–C64) → σ*(C62–S63), σ(C62–C64) → σ*(C62–S63), σ(C61–C62) → σ*(C6–C59), σ(C37–N38) → σ*(C33–C36) and σ(C61–C62) → σ*(C6–C59) at 0.51 kcal/mol in PCMR and 0.5 kcal/mol in PCMD1–D9 chromophores.

LP → π* and LP → σ* transitions are also observed conforming to resonance. LP → π* transitions involve LP(1)(C68) → π*(C64–C66), LP(1)(N82) → π*(C83–C85), LP(1)(N96) → π*(C107–C108), LP(2)(S20) → π*(C12–C13), LP(2)(S20) → π*(C12–C13), LP(1)(C3) → π*(C9–C57), LP(1)(N94) → π*(C95–C96), LP(2)(O120) → π*(C118–O119), LP(3)(O117) → π*(N115–O116) and LP(1)(N94) → π*(C105–C106) orbitals having 72.81, 35.44, 35.42, 29.54, 29.56, 69.77, 35.34, 47.48, 194.76 and 35.4 kcal/mol in PCMR and PCMD1–D9 chromophores, correspondingly. All the same, LP(1)(O35) → σ*(C29–C34), LP(1)(O35) → σ*(C29–C34), LP(1)(O35) → σ*(C29–C34), LP(1)(O35) → σ*(C23–C34), LP(3)(O119) → σ*(S118–O122), LP(1)(O35) → σ*(C29–C34), LP(1)(O116) → σ*(C115–O117), LP(1)(O35) → σ*(C29–C34) and LP(1)(O35) → σ*(C29–C34) at 21.25, 21.3, 20.67, 20.8, 18.57, 29.05, 21.11, 33.38, 21.35 and 21.24 kcal/mol in PCMR and PCMD1–D9 chromophores, correspondingly. Hence, this analysis reveals that their stabilization is significantly influenced by extended hyperconjugation and strong intramolecular charge transfer. These factors underscore the importance of their charge transfer properties, which are pivotal for their nonlinear optical (NLO) characteristics.

### Global reactivity descriptors

Global reactivity parameters (GRPs) encompass multiple descriptors i.e. global softness (*σ*), chemical potential (*μ*)^32^, ionization potential (*IP*)^33^, electronegativity (*X*)^34^, global hardness (*η*)^35^, electron affinity (*EA*) and global electrophilicity index (*ω*)^36^ that impart knowledge about molecular stability and its reactivity. Ionization potential (*IP*) is the principal parameter which defines energy needed to eliminate electron out of MO. From Table 3, highest energy of ionization potential of 5.94 eV is demonstrated by PCMD9 manifesting efficient electron contribution by donor group towards the acceptor part. While the least *IP* value is present in PCMD1 i.e. 5.64 eV. The trend for *IP* value is; PCMD9 > PCMD4 = PCMD7 > PCMD8 > PCMD5 > PCMD3 = PCMR > PCMD2 > PCMD1. Conversely, energy liberates on electronic insertion in valence shell is termed as electron affinity (*EA*). The highest *EA* is exhibited by PCMD7 at 3.65 eV while 3.35 eV is the least energy released upon electronic addition in PCMD1. Knowledge about polarization of electronic cloud is collected from global softness (*σ*) and global hardness (*η*) values. High polarization of electronic cloud is demonstrated by softer molecules while least is associated with harder compounds. The highest *η* value is present in PCMD9 at 1.22 eV that reduces to 1.02 eV in PCMD7. The values of global softness (*σ*) are lesser than their respective global hardness (*η*) and global electrophilicity index (*ω*) values. PCMD7 manifests highest softness at 4.88 eV^−1^ with 0.436 eV^−1^ being the least softness among all compounds present in PCMD1. Hence all the fabricated chromophores exhibited the higher value of softness which supported the greater charge transference in them than reference chromophore which in result would express good NLO response. The highest value of global electrophilicity index (*ω*) is possessed by PCMD4 at 10.47 eV. While the lowest of 8.86 eV is present in PCMR. Chemical potential (*μ*) is inversely related to Electronegativity (*X*) value. Where, chemical potential describes affinity of electron removal while electronegativity reveals affinity to accept electrons. PCMD9 with highest electronegativity and lowest chemical potential at 4.72 and − 4.72 eV, correspondingly, manifests highest electron transmission in turn possess highest nonlinearity.

**Table 3:** Quantum chemical descriptors values of PCMR and PCMD1–D9 in eV.

Compounds	*IP*	*EA*	*X*	*η*	*μ*	*ω*	*σ*
PCMR	5.66	3.36	4.51	1.15	− 4.51	8.86	0.436
PCMD1	5.64	3.35	4.50	1.14	− 4.50	8.90	0.440
PCMD2	5.65	3.40	4.53	1.12	− 4.53	9.11	0.445
PCMD3	5.66	3.45	4.55	1.11	− 4.55	9.36	0.452
PCMD4	5.70	3.62	4.66	1.04	− 4.66	10.47	0.482
PCMD5	5.68	3.53	4.60	1.08	− 4.60	9.82	0.464
PCMD6	5.66	3.46	4.56	1.10	− 4.56	9.45	0.455
PCMD7	5.70	3.65	4.67	1.02	− 4.67	10.7	0.488
PCMD8	5.69	3.62	4.66	1.04	− 4.66	10.5	0.482
PCMD9	5.94	3.51	4.72	1.22	− 4.72	9.16	0.411

*σ* is in eV^−1^.

### Transition density matrix

Transition density matrices (TDMs) is employed for visualizing and investigating optical nature of each electronic transition in a molecular system during excited state charge dispersion and electron–hole mobility^50^. Attaining transition density is the principal cause of TDMs inquiry. Off diagonal elements of transition density matrix manifest various basis function couplings during electronic transition. These varied basis functions (correspondent to diverse atomic centers) demonstrate density of transition which is the estimation of excitations involving charge transmission. Whereas, local excitations corresponds to transition density demonstrated by equivalent basis functions (correspondent to same atomic centers)^51^.

**Figure 6:**
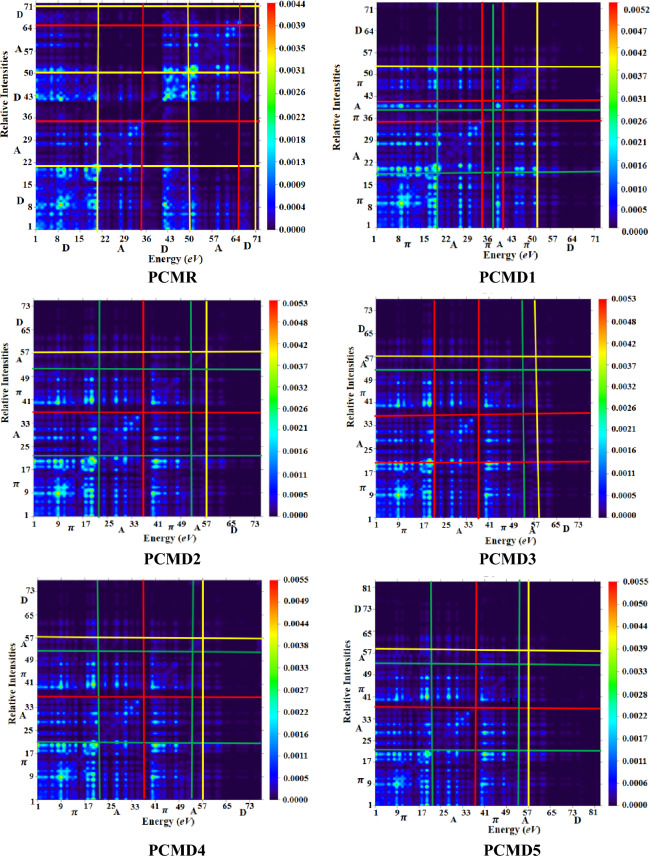

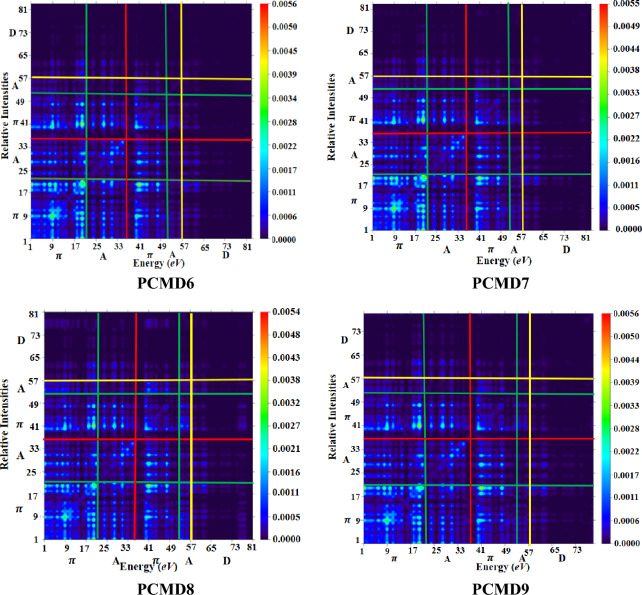
Pictorial representation of TDMs heat maps for PCMR and PCMD1–D9 illustrated using Multiwfn 3.7 program (http://sobereva.com/multiwfn/).

The current inquiry of PCMR and PCMD1–D9 has been executed at M06/6-311G(d,p) via Multiwfn 3.7 (Fig. 6). The formulated compounds (PCMD1–D9) have been categorized into D, π-bridge and A, however the reference is distributed into two acceptors and a π-bridge. In reference compound, the density is mainly localized over donor with minor transmission over acceptor groups. The bright points in the TDMs graphs manifests charge localization on donor group with very minute disposition on acceptor indicating less charge transmission towards acceptor in the case of reference compound. However, in the case of derivatives the major dispersion of charge density is over π-bridge and acceptor as indicated by bright points with almost no density localization over donor group. This shows efficient charge density transmission from donor to π-bridge and then towards acceptor in the derivatives. Therefore, transitions in derivatives are charge transmission excitations accompanied by considerable charge consistency in off-diagonal along with diagonal elements.

### Nonlinear optical activity

NLO is an eminent discipline of current investigations due to its importance in managing primary tasks of optical memory, telecommunications, signal processing, frequency shifting, optical interconnections, optical logic, optical switching and optical modulation^52–54^. Hyperpolarizability and ICT describes the correlation between nonlinearity and molecular structure. Molecular high polarizability, dipole moment as well as hyperpolarizability corresponds to enhanced/large NLO behavior. The electric field strength that deforms electronic dissemination throughout the compound is termed as linear polarizability (*α*). While atomic and molecular nonlinearity subject to extensive nonlinear optical phenomenon is hyperpolarizability (*β*, *γ*, etc.). The first order hyperpolarizability (*β*_*tot*_), second order hyperpolarizability (*γ*_*tot*_), linear polarizability (<*α*>) and dipole moment (*µ*_*tot*_) values including tensors for PCMR and PCMD1–D9 are presented in Tables S23–S26 and Table 4.

**Table 4:** The dipole moment (*µ*_*tot*_), linear polarizability (<*α*>), first order hyperpolarizability (*β*_*tot*_) and second order hyperpolarizability (*γ*_*tot*_) (in *esu.*) values of PCMR and PCMD1–D9.

Compounds	*µ*_*tot*_ (× 10^–17^)	< *α* > (× 10^–22^)	*β*_*tot*_ (× 10^–27^)	*γ*_*tot*_ (× 10^–32^)
PCMR	0.308	2.499	0.099	3.958
PCMD1	1.128	2.310	2.750	3.517
PCMD2	0.981	2.495	3.022	4.038
PCMD3	1.115	2.506	3.223	4.355
PCMD4	1.171	2.608	3.486	4.849
PCMD5	1.995	2.712	4.572	6.495
PCMD6	1.425	2.588	3.904	5.276
PCMD7	1.188	2.670	3.614	4.955
PCMD8	1.846	2.686	4.747	6.867
PCMD9	1.846	2.695	4.606	6.622

The dipole polarizability (*µ*) is greatly affected by difference of electronegativity that could be categorically the product of charge magnitude and distance among them, where high electronegativity corresponds to large *µ*^55^. Besides, molecular *µ*, polarity is crucial in improving molecular nonlinearity. The *µ*_*tot*_ characterize average dipole moment, whereas, tensors contributes to *µ*_*tot*_ along x, y and z-orientations are *µ*_x_, *µ*_y_ and *µ*_z_^56^. The highest *µ*_*tot*_ values are detected in all the derivatives (PCMD1–D9) in 0.981–1.995 × 10^–17^ esu range contrary to reference (PCMR) at 0.308 × 10^–17^ *esu*. The descending order of *µ*_*tot*_ is; PCMD5 > PCMD8 = PCMD9 > PCMD6 > PCMD7 > PCMD4 > PCMD1 > PCMD3 > PCMD2 > PCMR. The findings indicate major contribution by x-axis in the overall *µ*_*tot*_ (Table S22). The PCMD5 chromophore having highest *µ*_*tot*_ of 1.995 × 10^–17^ esu with *µ*_x_ = 1.953 × 10^–17^ esu, *µ*_y_ = 3.734 × 10^–18^ esu and *µ*_z_ = 1.561 × 10^–18^ esu contribution by tensors possess highest polarizability. Specifically, for the comparison with para Nitroaniline (*p*NA)^57^, a standard molecule for investigating the NLO properties, these compounds exhibit 0.06, 0.23, 0.20, 0.22, 0.24, 0.40, 0.29, 0.24, and 0.37 times greater *µ* values. Similarly, when compared to CPTR1^58^, similar analog to our compounds, 0.8, 3.0, 2.6, 3.0, 3.2, 5.4, 3.8, 3.2, and 5.0 the times greater values are found in our fabricated chromophores.

Table S22 indicates linear polarizability (<*α*>) values of PCMR and PCMD1–D9 along with polarizability contributing tensors and their values. The <*α*> value of 2.712 × 10^–22^ esu is the highest polarizability value among all the chromophores exhibited by PCMD5. However, the least <*α*> value of 2.310 × 10^–22^ esu corresponds to PCMD1. The <*α*> values for the rest of the compounds PCMR, PCMD2-D4, PCMD6-D9 are 2.499, 2.495, 2.506, 2.608, 2.588, 2.670, 2.686 and 2.695 × 10^–22^ esu, respectively. The investigation extends to the realm of linear polarizability, where a comparison is drawn between our computed compounds (PCMR and PCMD1–D9) and the standard pNA^57^ and CPTR1^58^. Notably, the linear polarizability values of pNA^57^ and CPTR1^58^ stand at 1.178 × 10^–23^ and 1.370 × 10^–22^ esu, respectively. In contrast, our designed derivatives exhibit intriguing linear polarizability values in relation to *p*NA and CPTR1. Correspondingly, the comprehensive analysis of their contributing tensors indicates major contribution by *α*_*xx*_ towards the overall <*α*> in all the compounds. The formulated chromophores have shown comparable <*α*> values with given descending order; PCMD5 > PCMD9 > PCMD8 > PCMD7 > PCMD4 > PCMD6 > PCMD3 > PCMR > PCMD2 > PCMD1.

Compound’s nonlinearity is calculated by first hyper-polarizability (*β*_*tot*_) value which increases in strongly push–pull configured molecular systems because of extended conjugation. The nine tensors i.e., *β*_xxx_, *β*_xxy_, *β*_xyy_, *β*_yyy_, *β*_xxz_, *β*_yyz_, *β*_xzz_, *β*_yzz_, *β*_zzz_ are used to determine hyperpolarizability values. Table S24 contains comprehensive findings of hyperpolarizability, whereas the average hyperpolarizability (*β*_*tot*_) values are tabulated in Table 4. The *β*_*tot*_ highest of 4.747 × 10^–27^ esu is exhibited by PCMD8, whereas the least hyperpolarizability is present in PCMR at 0.099 × 10^–27^ esu. The same trend for second hyperpolarizability values has been observed, where the highest *γ*_*tot*_ of 6.867 × 10^–32^ esu is exhibited by PCMD8, but the least *γ*_*tot*_ is present in PCMD1 at 3.517 × 10^–32^ esu. Where the major contribution towards *β*_*tot*_ is exhibited by *β*_*xxx*_ tensor and *γ*_*tot*_ in is by *γ*_*x*_ tensor. Comparative analysis with *p*NA and CPTR1 our compounds illustrated higher values for *β* and *γ*_*tot*_^50^_*.*_ In conclusion, the changing configuration from A-D-A to D–π–A and variant acceptor groups indicates substantial ICT besides bathochromic shift which in turn enhances the optical nonlinearity in all the designed chromophores. The high values of hyperpolarizability and polarizability manifest designed compounds as potential candidate for technologically advanced optical devices.

## Conclusion

In current report, we devised novel octacyclic naphthalene-based PCMD1–D9 nonlinear organic compounds by substituting one end acceptor moiety in PCMR with donor (9-phenyl-9H-carbazole) in derivatives and altering acceptor group in each successive derivative. Quantum chemical calculation were applied to investigate the NLO behavior of fabricated chromophores. The NBO study revealed a hyper conjugative interaction played significant role in stabilizing the molecule. An efficient charge transference from donor to acceptor through spacer has been studied by FMO findings which were further also supported by DOS and TDM analysis. A lower band gap (2.048 eV*)* with greater red shift (701.541 nm) is examined in PCMD8 than that of other molecules. From all the devised NLO compounds, PCMD8 demonstrated relatively high first hyperpolarizability and second hyperpolarizability of 4.747 × 10^–27^ esu and 6.867 × 10^–32^ esu, respectively. Nevertheless, PCMD5 manifested high dipole moment and linear polarizability value of 1.995 × 10^–17^ esu and 2.712 × 10^–22^ esu, respectively. So, PCMD8 and PCMD5 possessed high optoelectronic (linear and nonlinear) behavior resulted from the induction of nitro and sulfonic acid groups in the acceptor moiety. However, all the formulated compounds have shown higher nonlinearity on comparison with *p*NA as a standard molecule from the literature. Subsequently, PCMD8 chromophores is approved to be proficient NLO candidates for technologically advanced nonlinear optical devices.

### Supplementary Information

Supplementary Information.

## Data Availability

All data generated and analyzed during this study are included in this published article and its Supplementary Information files.
