# Supplementary material for: Promising impact of push–pull configuration into designed octacyclic naphthalene-based organic scaffolds for nonlinear optical amplitudes: a quantum chemical approach
Source: Sci Rep. 2023 Nov 16;13:20104. doi: 10.1038/s41598-023-44327-9 (PMC10654730; doi:10.1038/s41598-023-44327-9)
Supplement: Supplementary file 1 — Supplementary Information. [file 41598_2023_44327_MOESM1_ESM.docx]

**Promising Impact of Push-Pull Configuration into Designed Octacyclic Naphthalene-Based Organic Scaffolds for Nonlinear Optical Amplitudes: A Quantum Chemical Approach**

Muhammad Khalid,^1,2^ Iqra Shafiq,^1,2^ Muhammad Adnan Asghar*^3^, Ataualpa Albert Carmo Braga,^4^ Saad M. Alshehri,^5^ Muhammad Haroon^,6^ , Muhammed Lamin Sanyang,*^7^

^1^Institute of Chemistry, Khwaja Fareed University of Engineering & Information Technology, Rahim Yar Khan, 64200, Pakistan

^2^Centre for Theoretical and Computational Research, Khwaja Fareed University of Engineering & Information Technology, Rahim Yar Khan, 64200, Pakistan

^3^Department of Chemistry, Division of Science and Technology, University of Education Lahore, Pakistan

^4^Departamento de Química Fundamental, Instituto de Química, Universidade de São Paulo, Av. Prof. Lineu Prestes, 748, São Paulo, 05508-000, Brazil

^5^Department of Chemistry, College of Science, King Saud University, Saudi Arabia

^6^ Department of Chemistry and Biochemistry, Miami University, 651 E. High Street, Oxford, Ohio 45056, United States

^7^Directorate of Research and Consultancy, University of The Gambia, Kanifing Campus, MDI Road, P.O Box 3530, The Gambia

*Corresponding author's E-mail addresses:

Dr. Muhammad Adnan Asghar (adnan.muhammad@ue.edu.pk)

Dr. Muhammed Lamin Sanyang (m.sanyang@utg.edu.gm)

**Table S1:** Cartesian Coordinates of **PCMR** compound.

| **Atoms** | **X-axis** | **Y-axis** | **Z-axis** |
| --- | --- | --- | --- |
| C | 1.234875 | 1.400931 | 0.313254 |
| C | -0.00455 | 0.716157 | 0.310453 |
| C | 0.004528 | -0.71632 | 0.310155 |
| C | 1.233812 | -1.42759 | 0.283101 |
| C | 2.406552 | -0.73887 | 0.257201 |
| C | 2.407326 | 0.685713 | 0.27401 |
| H | 1.213982 | -2.51348 | 0.254828 |
| C | -2.40657 | 0.738722 | 0.257553 |
| C | -2.40734 | -0.68587 | 0.273776 |
| C | -3.78164 | -1.10633 | 0.216929 |
| C | -4.61161 | -0.00931 | 0.175339 |
| C | -5.95817 | -0.38864 | 0.107141 |
| C | -6.12528 | -1.78286 | 0.097215 |
| S | -4.59542 | -2.63669 | 0.162318 |
| C | -1.23383 | 1.42744 | 0.283732 |
| C | 3.828401 | -1.27764 | 0.204061 |
| C | -3.82843 | 1.277503 | 0.20463 |
| C | -8.33587 | -1.09153 | -0.01469 |
| C | -7.45268 | -2.17368 | 0.031169 |
| S | -7.46432 | 0.452546 | 0.025727 |
| H | -7.80684 | -3.19833 | 0.016561 |
| C | -9.7245 | -1.33014 | -0.07579 |
| C | -10.8354 | -0.52678 | -0.14253 |
| H | -9.91442 | -2.39973 | -0.07417 |
| C | -14.0715 | 2.806139 | -0.60381 |
| C | -14.9146 | 1.695087 | -0.61 |
| C | -14.4335 | 0.407445 | -0.48558 |
| C | -13.0581 | 0.238507 | -0.35313 |
| C | -12.2309 | 1.36253 | -0.35024 |
| C | -12.7089 | 2.651537 | -0.47171 |
| H | -15.1498 | -0.40399 | -0.50052 |
| H | -12.0467 | 3.510898 | -0.46627 |
| C | -12.2197 | -0.96825 | -0.20374 |
| C | -10.8265 | 0.942588 | -0.20985 |
| O | -9.86863 | 1.686964 | -0.1678 |
| C | -12.7145 | -2.24832 | -0.12196 |
| C | -11.9127 | -3.40829 | 0.056413 |
| N | -11.295 | -4.3742 | 0.203114 |
| C | -14.1014 | -2.54977 | -0.19546 |
| N | -15.2196 | -2.83548 | -0.25185 |
| C | -4.06867 | 2.099151 | -1.06133 |
| H | -3.82925 | 1.517448 | -1.95725 |
| H | -5.1175 | 2.410885 | -1.12305 |
| H | -3.44636 | 3.00054 | -1.05768 |
| C | -4.15789 | 2.097649 | 1.451861 |
| H | -3.97988 | 1.515576 | 2.36183 |
| H | -3.53741 | 2.999405 | 1.490973 |
| H | -5.20873 | 2.408766 | 1.442272 |
| C | 4.068629 | -2.09875 | -1.06226 |
| H | 5.117442 | -2.41049 | -1.12411 |
| H | 3.446291 | -3.00012 | -1.05899 |
| H | 3.82922 | -1.51665 | -1.95792 |
| C | 4.157784 | -2.09834 | 1.450947 |
| H | 3.979868 | -1.51662 | 2.36116 |
| H | 3.537175 | -3.00003 | 1.489701 |
| H | 5.208572 | -2.40964 | 1.441236 |
| F | -14.6107 | 4.009162 | -0.7291 |
| F | -16.2176 | 1.900943 | -0.74064 |
| C | -1.23489 | -1.4011 | 0.312697 |
| H | -1.21401 | 2.513333 | 0.255928 |
| C | 3.781621 | 1.106183 | 0.217294 |
| C | 4.611603 | 0.009178 | 0.175297 |
| C | 5.958158 | 0.388553 | 0.107126 |
| C | 6.125242 | 1.782786 | 0.097645 |
| S | 4.595374 | 2.636576 | 0.163124 |
| C | 7.452622 | 2.173659 | 0.031634 |
| S | 7.464327 | -0.45256 | 0.025327 |
| C | 8.335838 | 1.091538 | -0.01466 |
| H | 7.80675 | 3.198323 | 0.017321 |
| C | 9.724452 | 1.330212 | -0.0758 |
| C | 10.83541 | 0.526911 | -0.14277 |
| H | 9.914326 | 2.399811 | -0.0739 |
| C | 14.07165 | -2.80597 | -0.60332 |
| C | 14.91471 | -1.69491 | -0.60934 |
| C | 14.43355 | -0.40727 | -0.48511 |
| C | 13.05815 | -0.23833 | -0.35302 |
| C | 12.231 | -1.36237 | -0.35027 |
| C | 12.70902 | -2.65138 | -0.47156 |
| H | 15.14987 | 0.404169 | -0.4999 |
| H | 12.04685 | -3.51075 | -0.46623 |
| C | 12.21966 | 0.968423 | -0.20395 |
| C | 10.82657 | -0.94245 | -0.21017 |
| O | 9.868661 | -1.68685 | -0.16834 |
| C | 12.71443 | 2.248541 | -0.12249 |
| C | 14.10129 | 2.550034 | -0.19593 |
| N | 15.21953 | 2.835773 | -0.25228 |
| C | 11.91253 | 3.408538 | 0.055375 |
| N | 11.29474 | 4.374474 | 0.201639 |
| O | 1.270277 | 2.765622 | 0.322378 |
| O | -1.27031 | -2.7658 | 0.321237 |
| C | 1.299482 | 3.327273 | 1.63115 |
| H | 2.170361 | 2.96639 | 2.192199 |
| H | 1.368888 | 4.40861 | 1.512401 |
| H | 0.388169 | 3.079087 | 2.188221 |
| F | 16.21773 | -1.90076 | -0.73962 |
| F | 14.61087 | -4.00899 | -0.72841 |
| C | -1.29927 | -3.32807 | 1.629742 |
| H | -2.17004 | -2.96746 | 2.191126 |
| H | -1.36869 | -4.40935 | 1.510474 |
| H | -0.38786 | -3.08015 | 2.186776 |

**Table S2**: Cartesian coordinates of **PCMD1** compound.

| **Atoms** | **X-axis** | **Y-axis** | **Z-axis** |
| --- | --- | --- | --- |
| C | -0.80415 | -0.9863 | -0.45331 |
| C | 0.466243 | -0.37061 | -0.33904 |
| C | 0.524102 | 1.045309 | -0.12744 |
| C | -0.67261 | 1.801547 | -0.00894 |
| C | -1.8756 | 1.173197 | -0.09888 |
| C | -1.94375 | -0.2326 | -0.32452 |
| H | -0.60273 | 2.86981 | 0.179107 |
| C | 2.86405 | -0.51679 | -0.27561 |
| C | 2.9315 | 0.893728 | -0.0837 |
| C | 4.323248 | 1.236668 | 0.029271 |
| C | 5.101139 | 0.107242 | -0.09211 |
| C | 6.462889 | 0.40771 | 0.017615 |
| C | 6.699082 | 1.776927 | 0.225159 |
| S | 5.210936 | 2.702938 | 0.295095 |
| C | -3.33981 | -0.58467 | -0.3487 |
| C | 1.659883 | -1.13654 | -0.40839 |
| C | 4.259142 | -1.12422 | -0.29726 |
| C | 8.873516 | 0.970008 | 0.20906 |
| C | 8.042946 | 2.089632 | 0.330018 |
| S | 7.925284 | -0.50882 | -0.04241 |
| H | 8.448254 | 3.082872 | 0.487898 |
| C | 10.27018 | 1.128411 | 0.288568 |
| C | 11.34244 | 0.270992 | 0.222771 |
| H | 10.51516 | 2.176515 | 0.437015 |
| C | 14.43931 | -3.21427 | -0.01372 |
| C | 15.33073 | -2.15426 | 0.146828 |
| C | 14.90206 | -0.84831 | 0.270402 |
| C | 13.53173 | -0.60848 | 0.230669 |
| C | 12.65489 | -1.68217 | 0.068301 |
| C | 13.08044 | -2.98913 | -0.0555 |
| H | 15.653 | -0.0782 | 0.392108 |
| H | 12.38291 | -3.81056 | -0.18157 |
| C | 12.74392 | 0.637754 | 0.326252 |
| C | 11.26679 | -1.18688 | 0.051648 |
| O | 10.27445 | -1.87432 | -0.07798 |
| C | 13.29491 | 1.889622 | 0.477344 |
| C | 12.54348 | 3.093614 | 0.548611 |
| N | 11.969 | 4.095002 | 0.608798 |
| C | 14.69408 | 2.116182 | 0.574237 |
| N | 15.82416 | 2.343345 | 0.657768 |
| C | 4.563209 | -1.78306 | -1.64217 |
| H | 4.4175 | -1.07706 | -2.46613 |
| H | 5.600889 | -2.13558 | -1.6695 |
| H | 3.906231 | -2.64429 | -1.80316 |
| C | 4.454032 | -2.1189 | 0.846471 |
| H | 4.228445 | -1.65473 | 1.811943 |
| H | 3.796326 | -2.98484 | 0.716617 |
| H | 5.489544 | -2.47758 | 0.869899 |
| F | 14.92897 | -4.43984 | -0.12642 |
| F | 16.628 | -2.42734 | 0.179264 |
| C | 1.793179 | 1.661728 | -0.02106 |
| H | 1.584136 | -2.21257 | -0.5415 |
| O | 1.895119 | 3.009609 | 0.174164 |
| O | -0.89815 | -2.33541 | -0.66336 |
| C | 1.935853 | 3.761921 | -1.03425 |
| H | 2.786932 | 3.456973 | -1.65569 |
| H | 1.01045 | 3.637023 | -1.60903 |
| H | 2.049965 | 4.809274 | -0.75405 |
| C | -0.94418 | -2.69142 | -2.03984 |
| H | -1.80085 | -2.22069 | -2.53846 |
| H | -0.02341 | -2.394 | -2.55665 |
| H | -1.05088 | -3.77552 | -2.08746 |
| C | -3.27315 | 1.766932 | 0.013093 |
| C | -4.11699 | 0.529447 | -0.15765 |
| C | -5.48587 | 0.203175 | -0.14283 |
| C | -5.72421 | -1.15601 | -0.31917 |
| S | -4.24117 | -2.06263 | -0.5062 |
| S | -6.97537 | 1.069466 | 0.052417 |
| C | -7.09029 | -1.50649 | -0.31 |
| H | -7.47123 | -2.50984 | -0.45826 |
| C | -7.90059 | -0.41864 | -0.12268 |
| C | -9.35678 | -0.38808 | -0.04277 |
| C | -10.0845 | 0.75984 | -0.37246 |
| C | -10.0657 | -1.5214 | 0.371696 |
| C | -11.4639 | 0.78255 | -0.27826 |
| H | -9.56482 | 1.651486 | -0.71131 |
| C | -11.4463 | -1.51224 | 0.43983 |
| H | -9.52564 | -2.42002 | 0.653474 |
| C | -12.1554 | -0.35618 | 0.123603 |
| H | -12.0133 | 1.688855 | -0.51375 |
| H | -11.9853 | -2.40447 | 0.743488 |
| N | -13.5648 | -0.336 | 0.219519 |
| C | -14.4453 | 0.043702 | -0.78873 |
| C | -14.1759 | 0.410534 | -2.10273 |
| C | -15.7644 | -0.03001 | -0.29753 |
| C | -15.2531 | 0.720126 | -2.91692 |
| H | -13.1587 | 0.44815 | -2.47961 |
| C | -16.8301 | 0.286817 | -1.13651 |
| C | -16.5677 | 0.664179 | -2.44224 |
| H | -15.0705 | 1.010218 | -3.9471 |
| H | -17.8517 | 0.233914 | -0.77036 |
| H | -17.3873 | 0.915177 | -3.10795 |
| C | -14.3063 | -0.65062 | 1.354592 |
| C | -15.6756 | -0.47112 | 1.071968 |
| C | -13.8676 | -1.04785 | 2.612706 |
| C | -16.6212 | -0.71065 | 2.06621 |
| C | -14.8279 | -1.27996 | 3.583988 |
| H | -12.8109 | -1.16718 | 2.830231 |
| C | -16.1915 | -1.11798 | 3.317329 |
| H | -17.6797 | -0.57572 | 1.861095 |
| H | -14.5123 | -1.59204 | 4.574832 |
| H | -16.9161 | -1.31017 | 4.102003 |
| C | -3.52801 | 2.780999 | -1.10196 |
| H | -4.55378 | 3.164019 | -1.05443 |
| H | -2.84661 | 3.63328 | -1.00542 |
| H | -3.37525 | 2.326142 | -2.08611 |
| C | -3.49936 | 2.403932 | 1.383454 |
| H | -4.53208 | 2.759491 | 1.476879 |
| H | -3.30959 | 1.683246 | 2.185405 |
| H | -2.83156 | 3.260844 | 1.523177 |

**Table S3:** Cartesian Coordinates of **PCMD2** compound.

| **Atoms** | **X-axis** | **Y-axis** | **Z-axis** |
| --- | --- | --- | --- |
| C | 1.114233 | 0.894093 | 0.463959 |
| C | -0.14806 | 0.26102 | 0.356199 |
| C | -0.18864 | -1.1602 | 0.180084 |
| C | 1.017574 | -1.90581 | 0.093412 |
| C | 2.212743 | -1.26182 | 0.178696 |
| C | 2.263167 | 0.150287 | 0.365423 |
| H | 0.961683 | -2.97929 | -0.06714 |
| C | -2.54645 | 0.379312 | 0.268472 |
| C | -2.59731 | -1.03615 | 0.110432 |
| C | -3.98458 | -1.39629 | -0.00655 |
| C | -4.77484 | -0.27251 | 0.080137 |
| C | -6.13288 | -0.58864 | -0.03438 |
| C | -6.35411 | -1.9646 | -0.20966 |
| S | -4.85616 | -2.87761 | -0.24398 |
| C | -1.35039 | 1.015288 | 0.396336 |
| C | 3.61809 | -1.8421 | 0.096446 |
| C | -3.94765 | 0.972433 | 0.261566 |
| C | -8.53635 | -1.17726 | -0.23227 |
| C | -7.69444 | -2.29177 | -0.31947 |
| S | -7.60358 | 0.3157 | -0.00687 |
| H | -8.0898 | -3.29158 | -0.46045 |
| C | -9.9306 | -1.35129 | -0.32996 |
| C | -11.0139 | -0.50526 | -0.28953 |
| H | -10.1606 | -2.40463 | -0.46267 |
| C | -14.1682 | 2.965632 | 0.080555 |
| C | -15.0686 | 1.871214 | -0.09244 |
| C | -14.5735 | 0.554354 | -0.26216 |
| C | -13.2187 | 0.334209 | -0.25716 |
| C | -12.3396 | 1.433575 | -0.07784 |
| C | -12.7772 | 2.711664 | 0.085186 |
| H | -15.2967 | -0.24251 | -0.38988 |
| H | -12.0679 | 3.524818 | 0.218167 |
| C | -12.4107 | -0.89001 | -0.40863 |
| C | -10.9514 | 0.952015 | -0.09527 |
| O | -9.95612 | 1.639356 | 0.032223 |
| C | -12.9348 | -2.14562 | -0.6349 |
| C | -12.1584 | -3.32267 | -0.80469 |
| N | -11.5615 | -4.30271 | -0.94912 |
| C | -14.3282 | -2.39973 | -0.73527 |
| N | -15.4524 | -2.65435 | -0.8242 |
| C | -4.14187 | 1.935482 | -0.90926 |
| H | -3.90234 | 1.449452 | -1.86053 |
| H | -5.18088 | 2.282175 | -0.95129 |
| H | -3.49469 | 2.811587 | -0.79559 |
| C | -4.27165 | 1.663184 | 1.585663 |
| H | -4.12542 | 0.981436 | 2.429681 |
| H | -3.62629 | 2.536194 | 1.729294 |
| H | -5.31359 | 2.003793 | 1.594069 |
| C | 3.866188 | -2.5162 | -1.25218 |
| H | 4.906853 | -2.85265 | -1.32676 |
| H | 3.216455 | -3.39006 | -1.37013 |
| H | 3.668662 | -1.82403 | -2.07704 |
| C | 3.874588 | -2.81993 | 1.243187 |
| H | 3.70793 | -2.33862 | 2.212402 |
| H | 3.203853 | -3.68263 | 1.16586 |
| H | 4.905061 | -3.19216 | 1.215909 |
| C | -1.45024 | -1.79283 | 0.07644 |
| H | -1.28696 | 2.09493 | 0.503622 |
| C | 3.654556 | 0.519891 | 0.386972 |
| C | 4.446113 | -0.59021 | 0.23582 |
| C | 5.810687 | -0.24735 | 0.212426 |
| C | 6.031588 | 1.119741 | 0.346003 |
| S | 4.536405 | 2.013848 | 0.493416 |
| C | 7.391702 | 1.489106 | 0.301723 |
| S | 7.31089 | -1.10054 | 0.04369 |
| C | 8.215885 | 0.407374 | 0.138456 |
| H | 7.755931 | 2.507961 | 0.356849 |
| O | 1.191393 | 2.249428 | 0.637444 |
| O | -1.53584 | -3.14594 | -0.08916 |
| C | 1.228133 | 2.642697 | 2.004025 |
| H | 2.087827 | 2.194816 | 2.51826 |
| H | 1.322793 | 3.728788 | 2.022986 |
| H | 0.308565 | 2.348784 | 2.525025 |
| C | -1.57548 | -3.87252 | 1.134827 |
| H | -2.43408 | -3.56478 | 1.744384 |
| H | -1.6752 | -4.92696 | 0.876508 |
| H | -0.65538 | -3.72437 | 1.712605 |
| C | -16.4583 | 2.128461 | -0.09287 |
| H | -17.142 | 1.294618 | -0.22479 |
| C | -14.6908 | 4.266297 | 0.244973 |
| H | -13.998 | 5.092762 | 0.375832 |
| C | -16.9344 | 3.401856 | 0.069397 |
| H | -18.0038 | 3.58608 | 0.067021 |
| C | -16.0433 | 4.480441 | 0.2396 |
| H | -16.4359 | 5.484071 | 0.366784 |
| C | 9.670908 | 0.397723 | 0.043802 |
| C | 10.35625 | -0.64261 | -0.5916 |
| C | 10.42251 | 1.447471 | 0.585116 |
| C | 11.73512 | -0.6316 | -0.69567 |
| H | 9.801634 | -1.4605 | -1.0425 |
| C | 11.80097 | 1.460263 | 0.488404 |
| H | 9.920665 | 2.250762 | 1.115699 |
| C | 12.46752 | 0.421216 | -0.15636 |
| H | 12.25117 | -1.43151 | -1.21745 |
| H | 12.37394 | 2.268302 | 0.932868 |
| N | 13.87583 | 0.437202 | -0.26187 |
| C | 14.73609 | -0.57584 | 0.151884 |
| C | 16.06392 | -0.18891 | -0.12091 |
| C | 14.44306 | -1.78273 | 0.777457 |
| C | 17.11374 | -1.03644 | 0.224456 |
| C | 15.505 | -2.60715 | 1.112465 |
| H | 13.42036 | -2.0684 | 1.002101 |
| C | 16.82746 | -2.24437 | 0.836731 |
| H | 18.14146 | -0.74916 | 0.019923 |
| H | 15.30354 | -3.55541 | 1.601404 |
| H | 17.63412 | -2.91647 | 1.111328 |
| C | 14.63931 | 1.470894 | -0.79611 |
| C | 16.00187 | 1.117157 | -0.72725 |
| C | 14.22586 | 2.673214 | -1.35956 |
| C | 16.96679 | 1.994439 | -1.21584 |
| C | 15.20495 | 3.528462 | -1.83808 |
| H | 13.17415 | 2.932811 | -1.42599 |
| C | 16.56245 | 3.198812 | -1.76532 |
| H | 18.0204 | 1.733419 | -1.16832 |
| H | 14.90942 | 4.474673 | -2.28088 |
| H | 17.3023 | 3.894334 | -2.14832 |

**Table S4:** Cartesian Coordinates of **PCMD3** compound.

| **Atoms** | **X-axis** | **Y-axis** | **Z-axis** |
| --- | --- | --- | --- |
| C | 1.594892 | 0.805977 | 0.426785 |
| C | 0.346993 | 0.142885 | 0.331654 |
| C | 0.337227 | -1.28226 | 0.185907 |
| C | 1.559947 | -2.00295 | 0.11776 |
| C | 2.740459 | -1.33116 | 0.191488 |
| C | 2.759828 | 0.08532 | 0.346481 |
| H | 1.528982 | -3.08056 | -0.01918 |
| C | -2.05352 | 0.206521 | 0.239244 |
| C | -2.07336 | -1.21241 | 0.109555 |
| C | -3.45181 | -1.60495 | -0.00408 |
| C | -4.26688 | -0.49711 | 0.059082 |
| C | -5.617 | -0.84578 | -0.05258 |
| C | -5.80702 | -2.22996 | -0.20263 |
| S | -4.28875 | -3.10977 | -0.21556 |
| C | -0.87197 | 0.871153 | 0.354813 |
| C | 4.158374 | -1.88234 | 0.126063 |
| C | -3.46725 | 0.768817 | 0.218768 |
| C | -8.00643 | -1.49154 | -0.24583 |
| C | -7.1389 | -2.5886 | -0.31131 |
| S | -7.10713 | 0.025705 | -0.04346 |
| H | -7.51122 | -3.59927 | -0.43672 |
| C | -9.39527 | -1.69802 | -0.34624 |
| C | -10.4972 | -0.87429 | -0.31723 |
| H | -9.60174 | -2.75764 | -0.46733 |
| C | -13.7154 | 2.532272 | 0.065818 |
| C | -14.5961 | 1.421731 | -0.10748 |
| C | -14.0784 | 0.115301 | -0.28298 |
| C | -12.7192 | -0.0783 | -0.28253 |
| C | -11.8624 | 1.036388 | -0.10501 |
| C | -12.3212 | 2.306225 | 0.063008 |
| H | -14.7861 | -0.69499 | -0.40984 |
| H | -11.6264 | 3.131447 | 0.194499 |
| C | -11.8856 | -1.28679 | -0.43604 |
| C | -10.4641 | 0.58344 | -0.12717 |
| O | -9.48532 | 1.294287 | -0.00383 |
| C | -12.3857 | -2.55116 | -0.66469 |
| C | -11.5857 | -3.7117 | -0.8394 |
| N | -10.9685 | -4.67834 | -0.9877 |
| C | -13.7744 | -2.83056 | -0.763 |
| N | -14.8946 | -3.10294 | -0.84988 |
| C | -3.67988 | 1.705884 | -0.96995 |
| H | -3.42919 | 1.207571 | -1.91193 |
| H | -4.72575 | 2.030222 | -1.01932 |
| H | -3.0513 | 2.597367 | -0.87212 |
| C | -3.80769 | 1.477085 | 1.529481 |
| H | -3.6496 | 0.814163 | 2.386231 |
| H | -3.17973 | 2.365016 | 1.657964 |
| H | -4.85588 | 1.797817 | 1.530331 |
| C | 4.425268 | -2.57973 | -1.20716 |
| H | 5.472919 | -2.89584 | -1.27161 |
| H | 3.794415 | -3.46941 | -1.30811 |
| H | 4.215951 | -1.90976 | -2.04728 |
| C | 4.432352 | -2.82985 | 1.294106 |
| H | 4.25103 | -2.33252 | 2.252557 |
| H | 3.781922 | -3.70931 | 1.232898 |
| H | 5.471108 | -3.17899 | 1.278386 |
| C | -0.90996 | -1.94451 | 0.092933 |
| H | -0.83318 | 1.953824 | 0.439294 |
| C | 4.142542 | 0.485837 | 0.362552 |
| C | 4.958753 | -0.6099 | 0.24035 |
| C | 6.315526 | -0.23745 | 0.213676 |
| C | 6.505496 | 1.137437 | 0.314175 |
| S | 4.990334 | 2.001265 | 0.435259 |
| C | 7.857062 | 1.536161 | 0.264333 |
| S | 7.835202 | -1.06028 | 0.071253 |
| C | 8.706025 | 0.469623 | 0.13052 |
| H | 8.196358 | 2.56454 | 0.294732 |
| O | 1.642345 | 2.166181 | 0.569468 |
| O | -0.96542 | -3.30207 | -0.04628 |
| C | 1.670985 | 2.591691 | 1.926635 |
| H | 2.537246 | 2.17041 | 2.452089 |
| H | 1.747418 | 3.679347 | 1.920211 |
| H | 0.755999 | 2.29436 | 2.453729 |
| C | -0.99092 | -4.00653 | 1.19103 |
| H | -1.85734 | -3.70716 | 1.793603 |
| H | -1.06626 | -5.0675 | 0.952034 |
| H | -0.07549 | -3.82712 | 1.767314 |
| C | -15.9915 | 1.647059 | -0.10138 |
| H | -16.6867 | 0.824073 | -0.23073 |
| C | -14.2537 | 3.824985 | 0.23913 |
| H | -13.6034 | 4.683135 | 0.372932 |
| C | -16.4747 | 2.907176 | 0.068494 |
| C | -15.6024 | 4.000704 | 0.239489 |
| C | 10.16128 | 0.488992 | 0.040295 |
| C | 10.87077 | -0.55591 | -0.56033 |
| C | 10.88901 | 1.570272 | 0.551606 |
| C | 12.24957 | -0.52002 | -0.65872 |
| H | 10.33527 | -1.39891 | -0.9875 |
| C | 12.26741 | 1.608571 | 0.459879 |
| H | 10.369 | 2.378611 | 1.056201 |
| C | 12.95789 | 0.564067 | -0.1497 |
| H | 12.78501 | -1.32582 | -1.15117 |
| H | 12.82167 | 2.441075 | 0.882346 |
| N | 14.36607 | 0.603993 | -0.24867 |
| C | 15.24131 | -0.38216 | 0.197223 |
| C | 16.56362 | 0.018146 | -0.08249 |
| C | 14.96505 | -1.57462 | 0.856971 |
| C | 17.62545 | -0.80266 | 0.289511 |
| C | 16.03868 | -2.37206 | 1.218771 |
| H | 13.94599 | -1.86978 | 1.086094 |
| C | 17.35615 | -1.99666 | 0.935862 |
| H | 18.64921 | -0.50557 | 0.079186 |
| H | 15.85067 | -3.30869 | 1.734734 |
| H | 18.17245 | -2.64769 | 1.23189 |
| C | 15.11442 | 1.634193 | -0.81042 |
| C | 16.48242 | 1.305158 | -0.72631 |
| C | 14.68404 | 2.812283 | -1.41112 |
| C | 17.43482 | 2.183494 | -1.2371 |
| C | 15.651 | 3.669031 | -1.91127 |
| H | 13.62865 | 3.052811 | -1.48997 |
| C | 17.01336 | 3.364302 | -1.82351 |
| H | 18.49238 | 1.941402 | -1.17803 |
| H | 15.3417 | 4.59657 | -2.38323 |
| H | 17.74341 | 4.060188 | -2.22421 |
| F | -17.7806 | 3.14782 | 0.078263 |
| F | -16.1385 | 5.204194 | 0.400992 |

**Table S5:** Cartesian Coordinates of **PCMD4** compound.

| **Atoms** | **X-axis** | **Y-axis** | **Z-axis** |
| --- | --- | --- | --- |
| C | -2.07289 | 0.739958 | -0.42347 |
| C | -0.83768 | 0.051595 | -0.34079 |
| C | -0.85481 | -1.37489 | -0.20919 |
| C | -2.09114 | -2.07237 | -0.14437 |
| C | -3.25849 | -1.37717 | -0.20719 |
| C | -3.25132 | 0.040956 | -0.3466 |
| H | -2.08153 | -3.15171 | -0.01914 |
| C | 1.564609 | 0.065723 | -0.25816 |
| C | 1.556538 | -1.35402 | -0.13937 |
| C | 2.926305 | -1.77513 | -0.02889 |
| C | 3.764417 | -0.68392 | -0.08606 |
| C | 5.106503 | -1.06211 | 0.02763 |
| C | 5.265535 | -2.45133 | 0.174311 |
| S | 3.728957 | -3.29829 | 0.179135 |
| C | 0.39616 | 0.755101 | -0.36261 |
| C | -4.6862 | -1.90213 | -0.14384 |
| C | 2.989866 | 0.598918 | -0.23909 |
| C | 7.480539 | -1.76241 | 0.231571 |
| C | 6.587507 | -2.83995 | 0.290338 |
| S | 6.616484 | -0.22487 | 0.025517 |
| H | 6.935893 | -3.85894 | 0.417187 |
| C | 8.862063 | -2.00218 | 0.345449 |
| C | 9.984561 | -1.20488 | 0.324382 |
| H | 9.040869 | -3.06591 | 0.473355 |
| C | 13.28079 | 2.125805 | -0.03576 |
| C | 14.13229 | 1.00137 | 0.168207 |
| C | 13.58619 | -0.29405 | 0.346874 |
| C | 12.22424 | -0.45816 | 0.320219 |
| C | 11.39429 | 0.673819 | 0.116738 |
| C | 11.88095 | 1.931837 | -0.05656 |
| H | 14.27442 | -1.11692 | 0.496471 |
| H | 11.20652 | 2.770159 | -0.20905 |
| C | 11.36107 | -1.64772 | 0.465181 |
| C | 9.985855 | 0.25051 | 0.121225 |
| O | 9.025446 | 0.981929 | -0.02361 |
| C | 11.83051 | -2.922 | 0.70271 |
| C | 11.00298 | -4.06506 | 0.863085 |
| N | 10.36251 | -5.01812 | 0.999947 |
| C | 13.21131 | -3.23127 | 0.822367 |
| N | 14.32403 | -3.52696 | 0.926398 |
| C | 3.224478 | 1.527643 | 0.951977 |
| H | 2.967288 | 1.031059 | 1.893128 |
| H | 4.276411 | 1.831919 | 0.99925 |
| H | 2.612853 | 2.431471 | 0.859422 |
| C | 3.339247 | 1.304692 | -1.54894 |
| H | 3.168131 | 0.646566 | -2.40689 |
| H | 2.725333 | 2.202925 | -1.67372 |
| H | 4.392228 | 1.609083 | -1.55219 |
| C | -4.96317 | -2.60649 | 1.183746 |
| H | -6.01639 | -2.90342 | 1.248011 |
| H | -4.34886 | -3.50869 | 1.275047 |
| H | -4.73935 | -1.9482 | 2.029335 |
| C | -4.98052 | -2.83368 | -1.31975 |
| H | -4.79203 | -2.33124 | -2.27414 |
| H | -4.34665 | -3.72582 | -1.26825 |
| H | -6.02555 | -3.16359 | -1.3048 |
| C | 0.37876 | -2.06286 | -0.12583 |
| H | 0.380904 | 1.838994 | -0.43657 |
| C | -4.62637 | 0.467821 | -0.35461 |
| C | -5.46269 | -0.61383 | -0.24393 |
| C | -6.81214 | -0.21662 | -0.21171 |
| C | -6.97686 | 1.162898 | -0.29557 |
| S | -5.44577 | 1.999605 | -0.40842 |
| C | -8.32077 | 1.585854 | -0.23924 |
| S | -8.3466 | -1.0128 | -0.07822 |
| C | -9.18921 | 0.533532 | -0.11763 |
| H | -8.64029 | 2.620788 | -0.25709 |
| O | -2.09482 | 2.102367 | -0.55035 |
| O | 0.406439 | -3.42235 | 0.001894 |
| C | -2.12122 | 2.544724 | -1.9022 |
| H | -2.99508 | 2.142868 | -2.4302 |
| H | -2.18147 | 3.633232 | -1.88224 |
| H | -1.21249 | 2.24022 | -2.43595 |
| C | 0.420141 | -4.11697 | -1.24125 |
| H | 1.293497 | -3.83015 | -1.8399 |
| H | 0.473717 | -5.18113 | -1.01084 |
| H | -0.49048 | -3.91451 | -1.81756 |
| C | 15.5278 | 1.203043 | 0.187859 |
| H | 16.18863 | 0.356412 | 0.342909 |
| C | 13.85433 | 3.399155 | -0.21341 |
| H | 13.21069 | 4.258667 | -0.37002 |
| C | 16.06086 | 2.450835 | 0.013333 |
| C | 15.21198 | 3.565579 | -0.19117 |
| C | -10.6439 | 0.576977 | -0.02635 |
| C | -11.3712 | -0.46873 | 0.551396 |
| C | -11.3535 | 1.6809 | -0.51441 |
| C | -12.7494 | -0.41323 | 0.648042 |
| H | -10.8502 | -1.32943 | 0.960869 |
| C | -12.7313 | 1.739365 | -0.4236 |
| H | -10.8204 | 2.491541 | -1.00122 |
| C | -13.4395 | 0.692612 | 0.161289 |
| H | -13.2988 | -1.22144 | 1.120872 |
| H | -13.2717 | 2.589588 | -0.82849 |
| N | -14.8473 | 0.750646 | 0.256081 |
| C | -15.7337 | -0.21258 | -0.21661 |
| C | -17.0516 | 0.19642 | 0.071021 |
| C | -15.4698 | -1.39115 | -0.90556 |
| C | -18.1221 | -0.6028 | -0.32245 |
| C | -16.5519 | -2.1669 | -1.28855 |
| H | -14.4535 | -1.69208 | -1.13973 |
| C | -17.8654 | -1.7834 | -0.99792 |
| H | -19.1428 | -0.29973 | -0.10584 |
| H | -16.3739 | -3.09242 | -1.82758 |
| H | -18.6887 | -2.4174 | -1.31109 |
| C | -15.5838 | 1.774806 | 0.843675 |
| C | -16.9558 | 1.465325 | 0.748257 |
| C | -15.1398 | 2.929833 | 1.478074 |
| C | -17.8979 | 2.341182 | 1.282055 |
| C | -16.0968 | 3.784102 | 2.001255 |
| H | -14.0816 | 3.154462 | 1.565771 |
| C | -17.4627 | 3.499249 | 1.902684 |
| H | -18.9584 | 2.114602 | 1.214667 |
| H | -15.7767 | 4.693547 | 2.50043 |
| H | -18.1847 | 4.192467 | 2.322129 |
| Cl | 17.78347 | 2.648027 | 0.046121 |
| Cl | 15.87551 | 5.151756 | -0.41311 |

**Table S6:** Cartesian Coordinates of **PCMD5** compound.

| **Atoms** | **X-axis** | **Y-axis** | **Z-axis** |
| --- | --- | --- | --- |
| C | -3.38482 | -0.5929 | -0.18152 |
| C | -2.17251 | 0.139379 | -0.18118 |
| C | -2.23264 | 1.565907 | -0.0587 |
| C | -3.48877 | 2.223206 | 0.036127 |
| C | -4.63382 | 1.489715 | 0.015937 |
| C | -4.58379 | 0.069977 | -0.09442 |
| H | -3.51284 | 3.307461 | 0.101174 |
| C | 0.226454 | 0.218007 | -0.31672 |
| C | 0.175113 | 1.634862 | -0.18349 |
| C | 1.528963 | 2.111371 | -0.22747 |
| C | 2.40384 | 1.053853 | -0.36264 |
| C | 3.731528 | 1.492256 | -0.38531 |
| C | 3.839081 | 2.893108 | -0.27894 |
| S | 2.274178 | 3.673648 | -0.15387 |
| C | -0.91937 | -0.51618 | -0.31307 |
| C | -6.07595 | 1.97082 | 0.100392 |
| C | 1.666056 | -0.25915 | -0.44747 |
| C | 6.080067 | 2.307191 | -0.38883 |
| C | 5.142295 | 3.345157 | -0.28712 |
| S | 5.277523 | 0.726274 | -0.48714 |
| H | 5.449952 | 4.382614 | -0.21994 |
| C | 7.448052 | 2.615702 | -0.39085 |
| C | 8.607285 | 1.867769 | -0.42455 |
| H | 7.584407 | 3.691549 | -0.32561 |
| C | 12.03309 | -1.31727 | -0.04329 |
| C | 12.82408 | -0.13998 | 0.033064 |
| C | 12.23006 | 1.139323 | -0.07488 |
| C | 10.8713 | 1.228296 | -0.24536 |
| C | 10.09748 | 0.044298 | -0.30187 |
| C | 10.63603 | -1.20271 | -0.21306 |
| H | 12.87453 | 2.006567 | -0.00882 |
| H | 10.00523 | -2.08567 | -0.26252 |
| C | 9.960112 | 2.386177 | -0.37844 |
| C | 8.671379 | 0.403227 | -0.43373 |
| O | 7.751887 | -0.38648 | -0.51712 |
| C | 10.38237 | 3.693255 | -0.47588 |
| C | 9.511752 | 4.799113 | -0.66879 |
| N | 8.834996 | 5.722521 | -0.82974 |
| C | 11.749 | 4.076213 | -0.42602 |
| N | 12.84907 | 4.428836 | -0.38999 |
| C | 1.897003 | -0.94027 | -1.79654 |
| H | 1.631572 | -0.27283 | -2.62257 |
| H | 2.946502 | -1.23351 | -1.91159 |
| H | 1.284641 | -1.84504 | -1.87344 |
| C | 2.051774 | -1.19104 | 0.700333 |
| H | 1.883711 | -0.71066 | 1.669699 |
| H | 1.457469 | -2.11034 | 0.662232 |
| H | 3.109736 | -1.46918 | 0.631549 |
| C | -6.42326 | 2.893507 | -1.06747 |
| H | -7.47856 | 3.187162 | -1.03017 |
| H | -5.81977 | 3.806925 | -1.02685 |
| H | -6.23678 | 2.399327 | -2.02644 |
| C | -6.33914 | 2.669061 | 1.434698 |
| H | -6.09764 | 2.010214 | 2.275038 |
| H | -5.72744 | 3.573974 | 1.517753 |
| H | -7.39066 | 2.965634 | 1.519439 |
| C | -1.02258 | 2.297344 | -0.04726 |
| H | -0.8995 | -1.59631 | -0.42777 |
| C | -5.94546 | -0.39691 | -0.10455 |
| C | -6.81474 | 0.658953 | 0.006459 |
| C | -8.15308 | 0.222259 | -0.0045 |
| C | -8.27409 | -1.1598 | -0.12406 |
| S | -6.71824 | -1.94705 | -0.2417 |
| C | -9.60579 | -1.6221 | -0.14197 |
| S | -9.71628 | 0.967556 | 0.101761 |
| C | -10.5086 | -0.59856 | -0.02967 |
| H | -9.89786 | -2.65902 | -0.25679 |
| O | -3.36492 | -1.95637 | -0.29693 |
| O | -1.03495 | 3.656898 | 0.075187 |
| C | -3.31159 | -2.63425 | 0.952765 |
| H | -4.16994 | -2.36804 | 1.582248 |
| H | -3.34048 | -3.70301 | 0.738415 |
| H | -2.38687 | -2.39731 | 1.493254 |
| C | -1.0033 | 4.112725 | 1.424572 |
| H | -0.12191 | 3.72276 | 1.948243 |
| H | -0.95246 | 5.201063 | 1.391557 |
| H | -1.90517 | 3.805344 | 1.966897 |
| C | 14.21776 | -0.27023 | 0.228948 |
| H | 14.83043 | 0.623516 | 0.295251 |
| C | 12.66573 | -2.57337 | 0.070252 |
| H | 12.06214 | -3.47431 | 0.026063 |
| C | 14.81465 | -1.4942 | 0.340235 |
| C | 14.01818 | -2.67332 | 0.24953 |
| C | -11.9633 | -0.69214 | 0.005795 |
| C | -12.778 | 0.371609 | -0.3943 |
| C | -12.5831 | -1.86751 | 0.445823 |
| C | -14.1567 | 0.264344 | -0.36841 |
| H | -12.3275 | 1.288387 | -0.76431 |
| C | -13.9601 | -1.97675 | 0.48094 |
| H | -11.9761 | -2.69597 | 0.797959 |
| C | -14.7583 | -0.9112 | 0.071074 |
| H | -14.7752 | 1.087773 | -0.71181 |
| H | -14.4269 | -2.88378 | 0.852604 |
| N | -16.1653 | -1.02452 | 0.102832 |
| C | -17.0421 | -0.14178 | 0.728006 |
| C | -18.363 | -0.59184 | 0.529906 |
| C | -16.7659 | 0.994118 | 1.481402 |
| C | -19.4252 | 0.120882 | 1.079999 |
| C | -17.8396 | 1.684942 | 2.019184 |
| H | -15.7465 | 1.326753 | 1.649091 |
| C | -19.1573 | 1.260195 | 1.818888 |
| H | -20.4473 | -0.21766 | 0.933507 |
| H | -17.6513 | 2.575348 | 2.611114 |
| H | -19.9736 | 1.828135 | 2.253468 |
| C | -16.9125 | -2.03675 | -0.49389 |
| C | -18.2804 | -1.80073 | -0.24991 |
| C | -16.4857 | -3.11814 | -1.25691 |
| C | -19.2338 | -2.67617 | -0.76379 |
| C | -17.4534 | -3.97414 | -1.75721 |
| H | -15.4327 | -3.2856 | -1.45937 |
| C | -18.8141 | -3.76213 | -1.51222 |
| H | -20.2912 | -2.50453 | -0.58206 |
| H | -17.1461 | -4.82707 | -2.3545 |
| H | -19.5445 | -4.45455 | -1.9182 |
| S | 14.63959 | -4.34124 | 0.399149 |
| O | 15.70526 | -4.35949 | 1.376824 |
| O | 13.52012 | -5.23569 | 0.535167 |
| S | 16.5963 | -1.46185 | 0.554358 |
| O | 17.17757 | -2.38821 | -0.38465 |
| O | 17.0141 | -0.08644 | 0.593038 |
| O | 15.35249 | -4.61605 | -1.00065 |
| O | 16.79074 | -1.99802 | 2.039201 |
| H | 14.71485 | -4.96607 | -1.64196 |
| H | 16.57508 | -2.95622 | 2.056778 |

**Table S7:** Cartesian Coordinates of **PCMD6** compound.

| **Atoms** | **X-axis** | **Y-axis** | **Z-axis** |
| --- | --- | --- | --- |
| C | 2.968113 | 0.577038 | 0.200049 |
| C | 1.748219 | -0.1375 | 0.117368 |
| C | 1.795901 | -1.55886 | -0.0577 |
| C | 3.046521 | -2.22305 | -0.17663 |
| C | 4.198482 | -1.50215 | -0.11692 |
| C | 4.160737 | -0.09087 | 0.076537 |
| H | 3.05857 | -3.29782 | -0.33613 |
| C | -0.65499 | -0.18021 | 0.095978 |
| C | -0.61632 | -1.59602 | -0.05774 |
| C | -1.97777 | -2.04864 | -0.13278 |
| C | -2.84089 | -0.97988 | -0.02536 |
| C | -4.17544 | -1.3924 | -0.0895 |
| C | -4.30328 | -2.78489 | -0.2497 |
| S | -2.74656 | -3.58935 | -0.33174 |
| C | 0.498974 | 0.534764 | 0.187554 |
| C | 5.637224 | -1.98933 | -0.22953 |
| C | -2.09242 | 0.318153 | 0.1332 |
| C | -6.53643 | -2.15842 | -0.19065 |
| C | -5.61624 | -3.21008 | -0.30391 |
| S | -5.70695 | -0.59773 | -0.01697 |
| H | -5.94161 | -4.23856 | -0.41426 |
| C | -7.91178 | -2.44254 | -0.19636 |
| C | -9.05605 | -1.67794 | -0.11091 |
| H | -8.06551 | -3.51343 | -0.29335 |
| C | -12.4308 | 1.588056 | -0.01247 |
| C | -13.2526 | 0.430749 | -0.03893 |
| C | -12.6803 | -0.86447 | -0.07334 |
| C | -11.3143 | -0.98976 | -0.0776 |
| C | -10.5114 | 0.177049 | -0.04163 |
| C | -11.0263 | 1.435907 | -0.01121 |
| H | -13.3503 | -1.71469 | -0.09721 |
| H | -10.3714 | 2.302559 | 0.013009 |
| C | -10.4218 | -2.16861 | -0.10899 |
| C | -9.0901 | -0.21208 | -0.04594 |
| O | -8.14857 | 0.555594 | -0.00961 |
| C | -10.8645 | -3.47346 | -0.11235 |
| C | -10.0112 | -4.6086 | -0.1019 |
| N | -9.35 | -5.55702 | -0.09033 |
| C | -12.2395 | -3.82882 | -0.11331 |
| N | -13.3466 | -4.16117 | -0.11523 |
| C | -2.38297 | 1.271001 | -1.02597 |
| H | -2.14891 | 0.801831 | -1.98699 |
| H | -3.44002 | 1.560448 | -1.03191 |
| H | -1.78293 | 2.182378 | -0.93119 |
| C | -2.4158 | 0.981577 | 1.471699 |
| H | -2.20584 | 0.303735 | 2.305391 |
| H | -1.81573 | 1.888198 | 1.60291 |
| H | -3.47378 | 1.264594 | 1.515248 |
| C | 5.895789 | -2.62317 | -1.59642 |
| H | 6.945324 | -2.92288 | -1.69515 |
| H | 5.277398 | -3.51801 | -1.72503 |
| H | 5.659834 | -1.92179 | -2.40318 |
| C | 5.975252 | -2.9709 | 0.892281 |
| H | 5.793981 | -2.52092 | 1.873851 |
| H | 5.361501 | -3.87442 | 0.807578 |
| H | 7.027267 | -3.27399 | 0.841093 |
| C | 0.577729 | -2.2749 | -0.12432 |
| H | 0.488938 | 1.616309 | 0.290326 |
| C | 5.525499 | 0.366115 | 0.089304 |
| C | 6.386851 | -0.68902 | -0.07558 |
| C | 7.728178 | -0.25933 | -0.06497 |
| C | 7.856858 | 1.116201 | 0.108863 |
| S | 6.306553 | 1.909989 | 0.245046 |
| C | 9.190149 | 1.573449 | 0.124304 |
| S | 9.288386 | -1.00746 | -0.20264 |
| C | 10.08744 | 0.551436 | -0.03301 |
| H | 9.486546 | 2.611096 | 0.220754 |
| O | 2.961343 | 1.933944 | 0.378212 |
| O | 0.579616 | -3.63089 | -0.28455 |
| C | 2.974031 | 2.326666 | 1.745698 |
| H | 3.850058 | 1.917174 | 2.263868 |
| H | 3.019935 | 3.415842 | 1.765884 |
| H | 2.066792 | 1.991077 | 2.262903 |
| C | 0.617991 | -4.35565 | 0.941085 |
| H | -0.2457 | -4.10664 | 1.569913 |
| H | 0.585506 | -5.41486 | 0.685611 |
| H | 1.538547 | -4.14313 | 1.497592 |
| C | -14.6533 | 0.600909 | -0.02846 |
| H | -15.279 | -0.28442 | -0.04111 |
| C | -13.0442 | 2.857133 | 0.009613 |
| H | -12.4086 | 3.735468 | 0.021756 |
| C | -15.2328 | 1.840699 | 0.003171 |
| C | -14.4054 | 3.001543 | 0.009065 |
| C | 11.54272 | 0.644805 | -0.04908 |
| C | 12.33485 | -0.26505 | -0.75594 |
| C | 12.18368 | 1.672223 | 0.652828 |
| C | 13.713 | -0.14664 | -0.77365 |
| H | 11.86499 | -1.05882 | -1.32984 |
| C | 13.56022 | 1.789183 | 0.645463 |
| H | 11.59354 | 2.367147 | 1.242275 |
| C | 14.33582 | 0.881274 | -0.0715 |
| H | 14.31442 | -0.84108 | -1.35208 |
| H | 14.04543 | 2.572151 | 1.219987 |
| N | 15.74298 | 0.999409 | -0.07893 |
| C | 16.64617 | -7.2E-05 | 0.272012 |
| C | 17.95672 | 0.50652 | 0.16047 |
| C | 16.40197 | -1.29705 | 0.710091 |
| C | 19.04026 | -0.30999 | 0.475098 |
| C | 17.4965 | -2.08914 | 1.016154 |
| H | 15.39094 | -1.67702 | 0.815319 |
| C | 18.80389 | -1.60685 | 0.897463 |
| H | 20.05514 | 0.069376 | 0.39329 |
| H | 17.33284 | -3.1063 | 1.358764 |
| H | 19.63775 | -2.25603 | 1.144411 |
| C | 16.46236 | 2.142408 | -0.41584 |
| C | 17.83943 | 1.874065 | -0.27974 |
| C | 16.00138 | 3.375364 | -0.86403 |
| C | 18.76807 | 2.86782 | -0.57946 |
| C | 16.9452 | 4.346402 | -1.15709 |
| H | 14.94061 | 3.571428 | -0.98456 |
| C | 18.31485 | 4.101702 | -1.01316 |
| H | 19.83228 | 2.672928 | -0.47909 |
| H | 16.61145 | 5.317942 | -1.50856 |
| H | 19.02599 | 4.88658 | -1.24985 |
| C | -14.951 | 4.40535 | 0.071466 |
| C | -16.7388 | 1.899319 | -0.02922 |
| F | -15.3651 | 4.719626 | 1.301472 |
| F | -14.0209 | 5.304308 | -0.25972 |
| F | -15.9778 | 4.588881 | -0.75947 |
| F | -17.2316 | 2.794696 | 0.827698 |
| F | -17.2788 | 0.721367 | 0.291437 |
| F | -17.1939 | 2.211146 | -1.24548 |

**Table S8:** Cartesian Coordinates of **PCMD7** compound.

| **Atoms** | **X-axis** | **Y-axis** | **Z-axis** |
| --- | --- | --- | --- |
| C | -2.89052 | 0.66829 | -0.49532 |
| C | -1.67115 | -0.04999 | -0.42699 |
| C | -1.72209 | -1.47409 | -0.27797 |
| C | -2.97353 | -2.13949 | -0.1829 |
| C | -4.12392 | -1.41625 | -0.23343 |
| C | -4.08482 | -0.00022 | -0.39013 |
| H | -2.9885 | -3.21725 | -0.04578 |
| C | 0.732955 | -0.09998 | -0.38998 |
| C | 0.688911 | -1.51665 | -0.2516 |
| C | 2.047084 | -1.97326 | -0.15242 |
| C | 2.917057 | -0.90824 | -0.23737 |
| C | 4.247758 | -1.32658 | -0.1137 |
| C | 4.360053 | -2.71754 | 0.065155 |
| S | 2.799491 | -3.51524 | 0.088767 |
| C | -0.419 | 0.619452 | -0.48079 |
| C | -5.5616 | -1.90645 | -0.13871 |
| C | 2.173695 | 0.393243 | -0.4072 |
| C | 6.596348 | -2.10698 | 0.125249 |
| C | 5.665246 | -3.15031 | 0.19965 |
| S | 5.789859 | -0.54514 | -0.12337 |
| H | 5.975428 | -4.17795 | 0.352967 |
| C | 7.964942 | -2.39698 | 0.264909 |
| C | 9.119059 | -1.64641 | 0.250502 |
| H | 8.098506 | -3.46365 | 0.419634 |
| C | 12.55944 | 1.548327 | 0.005531 |
| C | 13.35204 | 0.393775 | 0.263486 |
| C | 12.74728 | -0.87605 | 0.421282 |
| C | 11.38269 | -0.98655 | 0.326884 |
| C | 10.60911 | 0.174218 | 0.07645 |
| C | 11.15589 | 1.410987 | -0.08231 |
| H | 13.39421 | -1.72294 | 0.612648 |
| H | 10.52493 | 2.275041 | -0.2725 |
| C | 10.47072 | -2.14275 | 0.437957 |
| C | 9.184579 | -0.1959 | 0.025724 |
| O | 8.258482 | 0.567018 | -0.16965 |
| C | 10.88374 | -3.4352 | 0.680916 |
| C | 10.01023 | -4.5493 | 0.794543 |
| N | 9.333082 | -5.48132 | 0.89438 |
| C | 12.24637 | -3.79996 | 0.847313 |
| N | 13.34145 | -4.14227 | 0.988642 |
| C | 2.454669 | 1.343063 | 0.756523 |
| H | 2.201851 | 0.875514 | 1.7136 |
| H | 3.514633 | 1.621119 | 0.778582 |
| H | 1.865197 | 2.260432 | 0.653545 |
| C | 2.507687 | 1.060201 | -1.74175 |
| H | 2.318147 | 0.379526 | -2.57799 |
| H | 1.895183 | 1.957335 | -1.88104 |
| H | 3.560362 | 1.363043 | -1.77436 |
| C | -5.83217 | -2.58363 | 1.204389 |
| H | -6.89075 | -2.85481 | 1.290719 |
| H | -5.23736 | -3.49833 | 1.299855 |
| H | -5.57901 | -1.91755 | 2.035506 |
| C | -5.89799 | -2.84852 | -1.29465 |
| H | -5.71567 | -2.36483 | -2.25985 |
| H | -5.28341 | -3.7539 | -1.2406 |
| H | -6.94964 | -3.15465 | -1.25582 |
| C | -0.50665 | -2.19357 | -0.20764 |
| H | -0.40488 | 1.702388 | -0.56729 |
| C | -5.4502 | 0.45887 | -0.38166 |
| C | -6.30874 | -0.60204 | -0.24468 |
| C | -7.64813 | -0.17524 | -0.19818 |
| C | -7.78505 | 1.206433 | -0.29772 |
| S | -6.23737 | 2.007532 | -0.44415 |
| C | -9.1193 | 1.657679 | -0.2275 |
| S | -9.1959 | -0.93778 | -0.03193 |
| C | -10.007 | 0.624837 | -0.0794 |
| H | -9.41892 | 2.698345 | -0.25499 |
| O | -2.88132 | 2.029422 | -0.63711 |
| O | -0.51477 | -3.55148 | -0.06172 |
| C | -2.91606 | 2.458355 | -1.99311 |
| H | -3.80484 | 2.069649 | -2.50571 |
| H | -2.95316 | 3.54804 | -1.98384 |
| H | -2.02122 | 2.129261 | -2.53558 |
| C | -0.53043 | -4.26252 | -1.29563 |
| H | 0.345597 | -4.00811 | -1.90487 |
| H | -0.50472 | -5.32463 | -1.05154 |
| H | -1.43983 | -4.04198 | -1.8673 |
| C | 14.75491 | 0.539229 | 0.345806 |
| H | 15.36538 | -0.34272 | 0.513475 |
| C | 13.19887 | 2.797905 | -0.14052 |
| H | 12.59305 | 3.683534 | -0.30491 |
| C | 15.35531 | 1.759807 | 0.185856 |
| C | 14.56085 | 2.915085 | -0.04562 |
| C | -11.4594 | 0.695757 | 0.029301 |
| C | -12.1967 | -0.33177 | 0.626707 |
| C | -12.1569 | 1.805017 | -0.46386 |
| C | -13.573 | -0.25565 | 0.733509 |
| H | -11.6846 | -1.19501 | 1.041977 |
| C | -13.5333 | 1.883948 | -0.36318 |
| H | -11.6164 | 2.6027 | -0.96378 |
| C | -14.2516 | 0.85307 | 0.237273 |
| H | -14.1298 | -1.05056 | 1.220041 |
| H | -14.0648 | 2.737191 | -0.77343 |
| N | -15.6585 | 0.926746 | 0.335656 |
| C | -16.5543 | -0.03576 | -0.12082 |
| C | -17.8679 | 0.388126 | 0.164512 |
| C | -16.3021 | -1.22593 | -0.7941 |
| C | -18.9461 | -0.40758 | -0.21513 |
| C | -17.3915 | -1.99794 | -1.16354 |
| H | -15.2892 | -1.53878 | -1.02696 |
| C | -18.701 | -1.59957 | -0.87476 |
| H | -19.9636 | -0.09299 | -0.00037 |
| H | -17.2227 | -2.93235 | -1.69001 |
| H | -19.5304 | -2.23095 | -1.17681 |
| C | -16.3848 | 1.96565 | 0.909837 |
| C | -17.7595 | 1.665757 | 0.823112 |
| C | -15.9299 | 3.126968 | 1.52477 |
| C | -18.6932 | 2.556627 | 1.346644 |
| C | -16.8788 | 3.996523 | 2.03777 |
| H | -14.8698 | 3.345059 | 1.6056 |
| C | -18.2471 | 3.720664 | 1.948081 |
| H | -19.7556 | 2.337084 | 1.285917 |
| H | -16.5501 | 4.911217 | 2.521521 |
| H | -18.9624 | 4.425755 | 2.359147 |
| C | 16.84021 | 1.832203 | 0.082089 |
| O | 17.42209 | 2.483298 | -0.74373 |
| O | 17.43434 | 1.037451 | 0.970771 |
| C | 15.17934 | 4.269496 | -0.00572 |
| O | 16.0817 | 4.572011 | 0.728868 |
| O | 14.56753 | 5.116213 | -0.83295 |
| C | 15.03875 | 6.46459 | -0.79332 |
| H | 14.42824 | 7.016575 | -1.50466 |
| H | 14.92504 | 6.88016 | 0.210336 |
| H | 16.09116 | 6.508246 | -1.08164 |
| C | 18.85974 | 0.978077 | 0.888177 |
| H | 19.17817 | 0.308223 | 1.684008 |
| H | 19.17105 | 0.589112 | -0.0839 |
| H | 19.29051 | 1.971409 | 1.03104 |

**Table S9:** Cartesian Coordinates of **PCMD8** compound.

| **Atoms** | **X-axis** | **Y-axis** | **Z-axis** |
| --- | --- | --- | --- |
| C | 2.42169 | 0.718366 | 0.46348 |
| C | 1.19263 | 0.018234 | 0.387555 |
| C | 1.223324 | -1.40737 | 0.245925 |
| C | 2.465641 | -2.0922 | 0.165031 |
| C | 3.626175 | -1.38596 | 0.222236 |
| C | 3.606514 | 0.031318 | 0.37163 |
| H | 2.465753 | -3.17067 | 0.032639 |
| C | -1.21095 | 0.007058 | 0.328746 |
| C | -1.18788 | -1.41116 | 0.19862 |
| C | -2.5515 | -1.84725 | 0.092752 |
| C | -3.40526 | -0.76685 | 0.166081 |
| C | -4.74036 | -1.16402 | 0.043162 |
| C | -4.87609 | -2.55601 | -0.12467 |
| S | -3.32748 | -3.37905 | -0.13812 |
| C | -0.04877 | 0.708268 | 0.426526 |
| C | 5.057614 | -1.89713 | 0.142246 |
| C | -2.64308 | 0.52381 | 0.330195 |
| C | -7.10165 | -1.90469 | -0.18815 |
| C | -6.18671 | -2.9665 | -0.25523 |
| S | -6.26634 | -0.35453 | 0.047068 |
| H | -6.51536 | -3.98967 | -0.39974 |
| C | -8.47228 | -2.17 | -0.32011 |
| C | -9.61434 | -1.39437 | -0.30266 |
| H | -8.6289 | -3.23481 | -0.46597 |
| C | -12.9815 | 1.865682 | 0.005583 |
| C | -13.804 | 0.726387 | -0.2295 |
| C | -13.2314 | -0.55476 | -0.40824 |
| C | -11.8671 | -0.69055 | -0.34331 |
| C | -11.0675 | 0.452884 | -0.10737 |
| C | -11.5809 | 1.701418 | 0.066353 |
| H | -13.8989 | -1.38802 | -0.58727 |
| H | -10.9276 | 2.550357 | 0.246228 |
| C | -10.975 | -1.86448 | -0.47377 |
| C | -9.64685 | 0.054374 | -0.08186 |
| O | -8.70945 | 0.806608 | 0.095974 |
| C | -11.4194 | -3.14504 | -0.72117 |
| C | -10.5692 | -4.27464 | -0.85909 |
| N | -9.90999 | -5.21701 | -0.97812 |
| C | -12.7918 | -3.47828 | -0.87146 |
| N | -13.897 | -3.79145 | -1.00029 |
| C | -2.8996 | 1.466588 | -0.84502 |
| H | -2.64608 | 0.986301 | -1.79556 |
| H | -3.95491 | 1.760696 | -0.87838 |
| H | -2.29681 | 2.375543 | -0.74541 |
| C | -2.97896 | 1.208606 | 1.655047 |
| H | -2.80464 | 0.534728 | 2.500005 |
| H | -2.35664 | 2.099581 | 1.789722 |
| H | -4.02814 | 1.524448 | 1.675332 |
| C | 5.329268 | -2.5869 | -1.19418 |
| H | 6.384408 | -2.87403 | -1.26996 |
| H | 4.721985 | -3.49349 | -1.28858 |
| H | 5.092654 | -1.92276 | -2.03168 |
| C | 5.370765 | -2.83632 | 1.30711 |
| H | 5.186792 | -2.34396 | 2.267599 |
| H | 4.744208 | -3.73346 | 1.253383 |
| H | 6.41835 | -3.15709 | 1.279569 |
| C | -0.00253 | -2.1079 | 0.168985 |
| H | -0.04497 | 1.791658 | 0.50797 |
| C | 4.977601 | 0.470867 | 0.371522 |
| C | 5.82251 | -0.60271 | 0.246524 |
| C | 7.167934 | -0.19422 | 0.207436 |
| C | 7.32217 | 1.186082 | 0.300465 |
| S | 5.784924 | 2.009077 | 0.430893 |
| C | 8.662451 | 1.619814 | 0.237485 |
| S | 8.707145 | -0.97748 | 0.056723 |
| C | 9.537776 | 0.574888 | 0.10147 |
| H | 8.974682 | 2.656847 | 0.261003 |
| O | 2.431666 | 2.079898 | 0.5989 |
| O | -0.01656 | -3.46593 | 0.030873 |
| C | 2.464442 | 2.514513 | 1.953302 |
| H | 3.34483 | 2.115922 | 2.472751 |
| H | 2.516617 | 3.603497 | 1.939269 |
| H | 1.561833 | 2.200176 | 2.491662 |
| C | -0.01491 | -4.17148 | 1.268434 |
| H | -0.88783 | -3.89953 | 1.874502 |
| H | -0.05786 | -5.234 | 1.02884 |
| H | 0.896921 | -3.96342 | 1.840652 |
| C | -15.2047 | 0.900621 | -0.2996 |
| H | -15.8498 | 0.056045 | -0.5181 |
| C | -13.5874 | 3.126688 | 0.193568 |
| H | -12.9789 | 3.996607 | 0.416941 |
| C | -15.7571 | 2.133446 | -0.12852 |
| C | -14.9419 | 3.256007 | 0.133306 |
| C | 10.99158 | 0.628149 | 0.001158 |
| C | 11.72055 | -0.40893 | -0.58985 |
| C | 11.6985 | 1.731135 | 0.494976 |
| C | 13.098 | -0.34696 | -0.69187 |
| H | 11.20125 | -1.26799 | -1.00487 |
| C | 13.07583 | 1.795901 | 0.399085 |
| H | 11.16417 | 2.535396 | 0.990949 |
| C | 13.78582 | 0.756771 | -0.19711 |
| H | 13.64848 | -1.14857 | -1.1745 |
| H | 13.61437 | 2.644708 | 0.809321 |
| N | 15.19321 | 0.818839 | -0.29425 |
| C | 16.08183 | -0.14878 | 0.165504 |
| C | 17.39861 | 0.265538 | -0.11937 |
| C | 15.82099 | -1.33561 | 0.841355 |
| C | 18.47104 | -0.53637 | 0.263364 |
| C | 16.90486 | -2.11395 | 1.213869 |
| H | 14.80572 | -1.64105 | 1.073919 |
| C | 18.2173 | -1.72506 | 0.92565 |
| H | 19.4909 | -0.22903 | 0.048914 |
| H | 16.72919 | -3.0459 | 1.742462 |
| H | 19.04211 | -2.36122 | 1.230258 |
| C | 15.92736 | 1.851342 | -0.8702 |
| C | 17.29982 | 1.542353 | -0.78106 |
| C | 15.48107 | 3.01409 | -1.48873 |
| C | 18.24014 | 2.425812 | -1.30528 |
| C | 16.43636 | 3.876141 | -2.00232 |
| H | 14.42252 | 3.238951 | -1.57161 |
| C | 17.80267 | 3.591437 | -1.90998 |
| H | 19.30095 | 2.199307 | -1.24263 |
| H | 16.11441 | 4.791817 | -2.48876 |
| H | 18.52323 | 4.290823 | -2.32161 |
| N | -15.5156 | 4.558465 | 0.509242 |
| O | -14.921 | 5.550274 | 0.153386 |
| O | -16.5157 | 4.536816 | 1.191379 |
| N | -17.1997 | 2.267266 | -0.3905 |
| O | -17.5507 | 3.240893 | -1.01855 |
| O | -17.9182 | 1.37335 | -0.00612 |

**Table 10:** Cartesian Coordinates of **PCMD9** compound.

| **Atoms** | **X-axis** | **Y-axis** | **Z-axis** |
| --- | --- | --- | --- |
| C | -1.84912 | 0.772432 | -0.42823 |
| C | -0.60922 | 0.093301 | -0.3432 |
| C | -0.61634 | -1.33369 | -0.21419 |
| C | -1.84756 | -2.04075 | -0.15374 |
| C | -3.01974 | -1.35418 | -0.21818 |
| C | -3.02257 | 0.064383 | -0.35522 |
| H | -1.82998 | -3.12014 | -0.02989 |
| C | 1.792313 | 0.126864 | -0.25232 |
| C | 1.79439 | -1.29335 | -0.13715 |
| C | 3.165822 | -1.70483 | -0.0239 |
| C | 3.996839 | -0.60631 | -0.07554 |
| C | 5.339745 | -0.9751 | 0.03909 |
| C | 5.508521 | -2.36557 | 0.180847 |
| S | 3.977442 | -3.22324 | 0.179935 |
| C | 0.618858 | 0.806971 | -0.35955 |
| C | -4.44368 | -1.88955 | -0.15848 |
| C | 3.213154 | 0.671154 | -0.22651 |
| C | 7.718241 | -1.65947 | 0.242829 |
| C | 6.830697 | -2.74513 | 0.296377 |
| S | 6.842695 | -0.12647 | 0.042601 |
| H | 7.186414 | -3.76208 | 0.419062 |
| C | 9.097631 | -1.89004 | 0.354361 |
| C | 10.21735 | -1.08276 | 0.335184 |
| H | 9.285828 | -2.95286 | 0.476231 |
| C | 13.48962 | 2.2678 | -0.0352 |
| C | 14.34964 | 1.147203 | 0.157732 |
| C | 13.8139 | -0.15175 | 0.338233 |
| C | 12.45267 | -0.32209 | 0.322176 |
| C | 11.6159 | 0.804336 | 0.128322 |
| C | 12.09135 | 2.066707 | -0.04468 |
| H | 14.50822 | -0.97083 | 0.479469 |
| H | 11.41017 | 2.900607 | -0.18955 |
| C | 11.59394 | -1.51836 | 0.469855 |
| C | 10.20682 | 0.37032 | 0.138936 |
| O | 9.245827 | 1.101303 | 0.001755 |
| C | 12.0757 | -2.78783 | 0.705346 |
| C | 11.25677 | -3.93658 | 0.871466 |
| N | 10.62252 | -4.89293 | 1.012729 |
| C | 13.45954 | -3.08607 | 0.81823 |
| N | 14.57545 | -3.37105 | 0.916491 |
| C | 3.436478 | 1.59683 | 0.969151 |
| H | 3.180182 | 1.094576 | 1.90751 |
| H | 4.485721 | 1.909555 | 1.02095 |
| H | 2.817857 | 2.495948 | 0.877939 |
| C | 3.561937 | 1.384649 | -1.53229 |
| H | 3.398145 | 0.729157 | -2.39363 |
| H | 2.942193 | 2.279087 | -1.6551 |
| H | 4.612749 | 1.69644 | -1.53068 |
| C | -4.71792 | -2.60006 | 1.166356 |
| H | -5.76937 | -2.90369 | 1.227962 |
| H | -4.09821 | -3.49878 | 1.255498 |
| H | -4.49951 | -1.94327 | 2.014507 |
| C | -4.72947 | -2.81936 | -1.33794 |
| H | -4.54283 | -2.31262 | -2.29042 |
| H | -4.08961 | -3.70729 | -1.28807 |
| H | -5.77223 | -3.15638 | -1.3258 |
| C | 0.621875 | -2.01215 | -0.12899 |
| H | 0.594035 | 1.890829 | -0.43156 |
| C | -4.40023 | 0.481392 | -0.36435 |
| C | -5.22903 | -0.60655 | -0.25612 |
| C | -6.58117 | -0.21911 | -0.22342 |
| C | -6.75544 | 1.159369 | -0.30464 |
| S | -5.23059 | 2.007402 | -0.41546 |
| C | -8.10224 | 1.57277 | -0.24709 |
| S | -8.10988 | -1.02641 | -0.09082 |
| C | -8.96314 | 0.514052 | -0.12699 |
| H | -8.42919 | 2.605415 | -0.26267 |
| O | -1.88073 | 2.134645 | -0.55274 |
| O | 0.661078 | -3.37102 | -0.00444 |
| C | -1.90847 | 2.579148 | -1.90401 |
| H | -2.77928 | 2.172704 | -2.43349 |
| H | -1.97549 | 3.667197 | -1.8822 |
| H | -0.99719 | 2.281147 | -2.43708 |
| C | 0.678766 | -4.06385 | -1.24895 |
| H | 1.549142 | -3.76896 | -1.84794 |
| H | 0.741186 | -5.12775 | -1.01982 |
| H | -0.23414 | -3.8678 | -1.82364 |
| C | 15.74531 | 1.352667 | 0.163798 |
| H | 16.40718 | 0.505306 | 0.310662 |
| C | 14.05101 | 3.547078 | -0.21633 |
| H | 13.3957 | 4.399357 | -0.3641 |
| C | 16.27553 | 2.60643 | -0.01472 |
| C | 15.41199 | 3.725712 | -0.20924 |
| C | -10.418 | 0.547572 | -0.03418 |
| C | -11.1376 | -0.50233 | 0.54549 |
| C | -11.1352 | 1.646566 | -0.52209 |
| C | -12.516 | -0.45513 | 0.644943 |
| H | -10.6106 | -1.35943 | 0.954707 |
| C | -12.5132 | 1.696649 | -0.42862 |
| H | -10.608 | 2.459979 | -1.01062 |
| C | -13.2137 | 0.646289 | 0.15903 |
| H | -13.0594 | -1.26618 | 1.119715 |
| H | -13.0595 | 2.543102 | -0.83341 |
| N | -14.6215 | 0.696688 | 0.258084 |
| C | -15.5044 | -0.27184 | -0.21044 |
| C | -16.8234 | 0.131073 | 0.080461 |
| C | -15.2368 | -1.4501 | -0.89852 |
| C | -17.8912 | -0.67375 | -0.30893 |
| C | -16.3162 | -2.23156 | -1.27736 |
| H | -14.2198 | -1.74646 | -1.13533 |
| C | -17.6307 | -1.85397 | -0.98362 |
| H | -18.9127 | -0.3752 | -0.08986 |
| H | -16.1354 | -3.15693 | -1.81567 |
| H | -18.4518 | -2.49232 | -1.29367 |
| C | -15.3615 | 1.718062 | 0.846335 |
| C | -16.7321 | 1.401464 | 0.755495 |
| C | -14.9215 | 2.87635 | 1.477597 |
| C | -17.6771 | 2.273409 | 1.290543 |
| C | -15.8813 | 3.726663 | 2.002048 |
| H | -13.8642 | 3.106554 | 1.561909 |
| C | -17.246 | 3.434744 | 1.907913 |
| H | -18.7366 | 2.041263 | 1.226622 |
| H | -15.5643 | 4.638587 | 2.498718 |
| H | -17.9703 | 4.125016 | 2.328271 |
| C | 17.68951 | 2.789808 | -0.0059 |
| N | 18.83297 | 2.940802 | 0.000858 |
| C | 15.96155 | 5.027668 | -0.39634 |
| N | 16.4073 | 6.08059 | -0.54791 |

**Table S11**: Wavelength, excitation energy and oscillator strength of investigated compound (**PCMR and PCMD1-PCMD9**) at M06/6-311G (d,p).

| **Compounds** | **DFT**  ***λ* (*nm*)** | **E(*eV*)** | ***f*** | **MO contributions** |
| --- | --- | --- | --- | --- |
| **PCMR** | 644.907 | 2.043 | 2.956 | H→L (95%), H-1→L+1 (4%) |
|  | 526.715 | 2.361 | 0.003 | H→L+1 (97%), |
|  | 484.745 | 2.669 | 0.002 | H-1→L (89%), H→L+3 (6%) |
|  | 423.991 | 2.722 | 0.165 | H→L+2 (85%), H-1→L+3 (9%) |
|  | 421.096 | 2.807 | 0.003 | H→L+3 (78%), H-1→L (8%), H-1→L+2 (9%) |
|  | 400.450 | 2.830 | 0.447 | H-2→L (55%), H-1→L+1 (38%), H→L (2%) |
| **PCMD1** | 619.174 | 2.002 | 1.542 | H→L (94%), H-1→L (3%) |
|  | 505.662 | 2.452 | 0.352 | H-1→L (86%), H-3→L (8%), H→L (4%) |
|  | 479.570 | 2.585 | 0.327 | H→L+1 (83%), H-3→L+1 (5%), H-1→L+1 (8%) |
|  | 457.655 | 2.709 | 0.398 | H-3→L (78%), H-1→L (10%), H-4→L (8%), H→L+2 (3%) |
|  | 435.350 | 2.848 | 0.000 | H-2→L (100%), |
|  | 429.796 | 2.885 | 0.089 | H-4→L (84%), H-3→L (8%) |
| **PCMD2** | 616.648 | 2.011 | 1.802 | H→L (95%), H-1→L (3%) |
|  | 499.449 | 2.482 | 0.429 | H-2→L (11%), H-1→L (82%), H→L (4%) |
|  | 456.073 | 2.719 | 0.341 | H-2→L (68%), H-1→L (13%), H-4→L (9%), H→L+1 (5%), H→L+2 (2%) |
|  | 440.798 | 2.813 | 0.073 | H-5→L (16%), H-2→L (10%), H→L+1 (59%), H-2→L+1 (4%), H-1→L+1 (6%) |
|  | 426.235 | 2.909 | 0.175 | H-5→L (36%), H-4→L (38%), H→L+1 (16%), H→L+2 (2%) |
|  | 425.037 | 2.917 | 0.306 | H-5→L (36%), H-4→L (43%), H-2→L (6%), H→L+2 (8%) |
| **PCMD3** | 632.052 | 1.962 | 1.679 | H→L (95%), H-1→L (3%) |
|  | 513.155 | 2.416 | 0.443 | H-1→L (85%), H-3→L (9%), H→L (4%) |
|  | 465.420 | 2.664 | 0.435 | H-3→L (74%), H-1→L (11%), H-4→L (7%), H→L+1 (3%), H→L+2 (2%) |
|  | 449.557 | 2.758 | 0.128 | H→L+1 (74%), H-6→L (4%), H-3→L (6%), H-3→L+1 (5%), H-1→L+1 (7%) |
|  | 438.336 | 2.829 | 0.000 | H-2→L (100%), |
|  | 434.039 | 2.857 | 0.067 | H-4→L (85%), H-3→L (7%) |
| **PCMD4** | 641.238 | 1.934 | 1.687 | H→L (95%), H-1→L (3%) |
|  | 520.588 | 2.382 | 0.460 | H-1→L (85%), H-3→L (9%), H→L (4%) |
|  | 471.079 | 2.632 | 0.457 | H-3→L (75%), H-1→L (10%), H-4→L (7%), H→L+1 (4%), H→L+2 (2%) |
|  | 455.168 | 2.724 | 0.096 | H→L+1 (75%), H-6→L (4%), H-3→L (6%), H-3→L+1 (5%), H-1→L+1 (7%) |
|  | 445.007 | 2.786 | 0.000 | H-2→L (100%), |
|  | 438.351 | 2.828 | 0.070 | H-4→L (86%), H-3→L (6%) |
| **PCMD5** | 696.849 | 1.779 | 1.338 | H→L (95%), H-1→L (3%) |
|  | 568.940 | 2.179 | 0.436 | H-1→L (91%), H-3→L (5%), H→L (4%) |
|  | 514.689 | 2.409 | 0.247 | H→L+1 (84%), H-3→L+1 (4%), H-1→L+1 (7%) |
|  | 499.933 | 2.480 | 0.425 | H-3→L (85%), H-4→L (4%), H-1→L (5%), H→L+1 (3%) |
|  | 490.693 | 2.527 | 0.000 | H-2→L (100%), |
|  | 464.723 | 2.668 | 0.102 | H-4→L (90%), H-3→L (5%) |
| **PCMD6** | 663.580 | 1.868 | 1.504 | H→L (95%), H-1→L (3%) |
|  | 541.058 | 2.292 | 0.414 | H-1→L (89%), H-3→L (6%), H→L (4%) |
|  | 485.543 | 2.554 | 0.580 | H-3→L (71%), H→L+1 (12%), H-4→L (5%), H-1→L (7%) |
|  | 477.447 | 2.597 | 0.014 | H-3→L (14%), H→L+1 (73%), H-3→L+1 (4%), H-1→L+1 (6%) |
|  | 465.508 | 2.663 | 0.000 | H-2→L (100%), |
|  | 451.013 | 2.749 | 0.085 | H-4→L (89%), H-3→L (5%) |
| **PCMD7** | 637.021 | 1.946 | 1.734 | H→L (95%), H-1→L (3%) |
|  | 517.007 | 2.398 | 0.467 | H-1→L (85%), H-3→L (9%), H→L (4%) |
|  | 468.870 | 2.644 | 0.501 | H-3→L (65%), H-1→L (10%), H→L+1 (12%), H-4→L (7%), H→L+2 (3%) |
|  | 460.374 | 2.693 | 0.040 | H-3→L (15%), H→L+1 (69%), H-6→L (2%), H-3→L+1 (4%), H-1→L+1 (7%) |
|  | 441.300 | 2.810 | 0.000 | H-2→L (100%), |
|  | 436.054 | 2.843 | 0.077 | H-4→L (85%), H-3→L (7%) |
| **PCMD8** | 701.541 | 1.767 | 1.299 | H→L (96%), H-1→L (3%) |
|  | 573.015 | 2.164 | 0.442 | H-1→L (91%), H-3→L (4%), H→L (4%) |
|  | 542.549 | 2.285 | 0.118 | H→L+1 (89%), H-3→L+1 (3%), H-1→L+1 (6%) |
|  | 502.995 | 2.465 | 0.532 | H-3→L (87%), H-4→L (4%), H-1→L (5%) |
|  | 494.549 | 2.507 | 0.000 | H-2→L (100%), |
|  | 466.418 | 2.658 | 0.127 | H-4→L (89%), H-3→L (5%) |
| **PCMD9** | 695.442 | 1.783 | 1.362 | H→L (95%), H-1→L (3%) |
|  | 567.715 | 2.184 | 0.443 | H-1→L (90%), H-3→L (5%), H→L (4%) |
|  | 512.180 | 2.421 | 0.208 | H→L+1 (83%), H-3→L (3%), H-3→L+1 (4%), H-1→L+1 (7%) |
|  | 499.651 | 2.481 | 0.433 | H-3→L (84%), H-4→L (4%), H-1→L (5%), H→L+1 (4%) |
|  | 489.724 | 2.532 | 0.000 | H-2→L (100%), |
|  | 463.801 | 2.673 | 0.114 | H-4→L (90%), H-3→L (5%) |

**Table S12:** The major findings of ultraviolet-visible absorption wavelengths, excitation energy, molecular orbital contributions and oscillator strengths values for **PCMR** and **PCMD1**-**D9**.

| **Compounds** | **DFT**  ***λ*_max_ (*nm*)** | **E (*eV*)** | ***f*_os_** | **MO contributions** |
| --- | --- | --- | --- | --- |
| **PCMR** | 606.899 | 2.043 | 2.956 | H→L (95%), H-1→L+1 (4%) |
| **PCMD1** | 619.174 | 2.002 | 1.542 | H→L (94%), H-1→L (3%) |
| **PCMD2** | 616.648 | 2.011 | 1.802 | H→L (95%), H-1→L (3%) |
| **PCMD3** | 632.052 | 1.962 | 1.679 | H→L (95%), H-1→L (3%) |
| **PCMD4** | 641.238 | 1.934 | 1.687 | H→L (95%), H-1→L (3%) |
| **PCMD5** | 696.849 | 1.779 | 1.338 | H→L (95%), H-1→L (3%) |
| **PCMD6** | 663.580 | 1.868 | 1.504 | H→L (95%), H-1→L (3%) |
| **PCMD7** | 637.021 | 1.946 | 1.734 | H→L (95%), H-1→L (3%) |
| **PCMD8** | 701.541 | 1.767 | 1.299 | H→L (96%), H-1→L (3%) |
| **PCMD9** | 695.442 | 1.783 | 1.362 | H→L (95%), H-1→L (3%) |

**Table S13:** Natural bond orbitals representative values for **PCMR**.

| **Donor(*i*)** | **Type** | **Acceptor(*j*)** | **Type** | ***E*(2)**  **[*kcal/mol*]** | ***E*(*j*)-*E*(*i*)**  **[*a.u.*]** | **F(*i,j*)**  **[*a.u.*]** |
| --- | --- | --- | --- | --- | --- | --- |
| π | C18-C19 | π* | C22-C23 | 31.57 | 0.31 | 0.089 |
| π | C22-C23 | π* | C33-C36 | 26.78 | 0.3 | 0.08 |
| π | C12-C13 | π* | C18-C19 | 24.99 | 0.29 | 0.078 |
| π | C28-C29 | π* | C33-C36 | 21.83 | 0.29 | 0.073 |
| π | C76-C77 | π* | C82-O83 | 20.99 | 0.3 | 0.075 |
| π | C2-C3 | π* | C1-C6 | 19.83 | 0.29 | 0.07 |
| π | C61-C62 | π* | C1-C6 | 18.87 | 0.33 | 0.072 |
| π | C10-C11 | π* | C12-C13 | 18.44 | 0.28 | 0.069 |
| π | C12-C13 | π* | C10-C11 | 17.52 | 0.32 | 0.067 |
| π | C4-C5 | π* | C1-C6 | 16.98 | 0.31 | 0.067 |
| π | C18-C19 | π* | C12-C13 | 15.17 | 0.28 | 0.061 |
| π | C2-C3 | π* | C8-C15 | 14.97 | 0.31 | 0.064 |
| π | C1-C6 | π* | C61-C62 | 14.68 | 0.3 | 0.06 |
| π | C22-C23 | π* | C18-C19 | 11.41 | 0.29 | 0.053 |
| π | C33-C36 | π* | C28-C29 | 8.55 | 0.33 | 0.05 |
| π | C82-O83 | π* | C76-C77 | 4.18 | 0.42 | 0.042 |
| π | C82-O83 | π* | C70-C71 | 3.68 | 0.43 | 0.038 |
| π | C70-C71 | π* | C70-C71 | 2.68 | 0.31 | 0.026 |
| π | C1-C6 | σ* | O89-C91 | 1.72 | 0.57 | 0.03 |
| π | C61-C62 | π* | C61-C62 | 0.65 | 0.31 | 0.013 |
| σ | C22-H24 | σ* | C18-S20 | 10.27 | 0.71 | 0.076 |
| σ | C18-C19 | σ* | C13-S14 | 8.06 | 0.92 | 0.077 |
| σ | C5-C6 | σ* | C61-S65 | 7.39 | 0.87 | 0.072 |
| σ | C84-C85 | σ* | C81-C84 | 6.13 | 1.33 | 0.081 |
| σ | C33-C36 | σ* | C36-C39 | 6.06 | 1.27 | 0.078 |
| σ | C9-C59 | σ* | C3-C59 | 5.14 | 1.29 | 0.073 |
| σ | C4-C5 | σ* | C5-C16 | 4.58 | 1.16 | 0.065 |
| σ | C9-C10 | σ* | C8-C9 | 3.42 | 1.24 | 0.058 |
| σ | C29-C34 | σ* | C34-O35 | 2.11 | 1.26 | 0.046 |
| σ | C77-C82 | σ* | C82-O83 | 2.11 | 1.26 | 0.046 |
| σ | C17-C45 | σ* | C10-C11 | 1.01 | 1.18 | 0.031 |
| σ | C37-N38 | σ* | C33-C36 | 0.51 | 1.65 | 0.026 |
| LP(1) | O90 | π* | C9-C59 | 6.3 | 0.62 | 0.06 |
| LP(1) | S20 | σ* | C12-C13 | 3.29 | 1.18 | 0.056 |
| LP(1) | S67 | σ* | C66-C68 | 2.25 | 1.21 | 0.047 |
| LP(1) | O90 | σ* | C9-C59 | 1.32 | 1.19 | 0.036 |
| LP(1) | S14 | σ* | C13-C19 | 0.7 | 1.24 | 0.026 |
| LP(1) | S67 | σ* | C68-C70 | 0.51 | 1.22 | 0.022 |
| LP(1) | C68 | π* | C64-C66 | 72.81 | 0.17 | 0.111 |
| LP(1) | C68 | π* | C70-C71 | 65.97 | 0.18 | 0.116 |
| LP(1) | N86 | σ* | C84-C85 | 12.7 | 1.04 | 0.103 |
| LP(1) | N38 | σ* | C36-C37 | 12.66 | 1.04 | 0.103 |
| LP(2) | S20 | π* | C12-C13 | 29.36 | 0.25 | 0.079 |
| LP(2) | S65 | π* | C61-C62 | 26.31 | 0.28 | 0.077 |
| LP(2) | S20 | π* | C18-C19 | 22.01 | 0.26 | 0.068 |
| LP(2) | O35 | σ* | C29-C34 | 21.25 | 0.76 | 0.115 |
| LP(2) | O35 | σ* | C23-C34 | 18.8 | 0.76 | 0.108 |
| LP(2) | O89 | σ* | C1-C2 | 8.55 | 0.89 | 0.078 |
| LP(2) | F95 | σ* | C73-C74 | 7.61 | 0.97 | 0.077 |
| LP(2) | O89 | σ* | C1-C6 | 6.86 | 0.94 | 0.072 |
| LP(2) | O89 | σ* | C91-H94 | 6.58 | 0.7 | 0.061 |
| LP(2) | F57 | σ* | C25-C30 | 6.33 | 1.01 | 0.072 |
| LP(2) | F58 | σ* | C26-C27 | 6.15 | 1.01 | 0.07 |
| LP(2) | O89 | σ* | C91-H92 | 5.35 | 0.7 | 0.055 |
| LP(2) | O35 | σ* | C12-S20 | 2.32 | 0.52 | 0.032 |
| LP(3) | F96 | π* | C73-C78 | 20.58 | 0.46 | 0.093 |
| LP(3) | F95 | π* | C74-C75 | 20.54 | 0.46 | 0.093 |

**Table S14:** Natural bond orbitals representative values for **PCMD1**.

| **Donor(*i*)** | **Type** | **Acceptor(*j*)** | **Type** | ***E*(2)**  **[*kcal/mol*]** | ***E*(*j*)-*E*(*i*)**  **[*a.u.*]** | **F(*i,j*)**  **[*a.u.*]** |
| --- | --- | --- | --- | --- | --- | --- |
| π | C18-C19 | π* | C22-C23 | 32.39 | 0.31 | 0.09 |
| π | C22-C23 | π* | C33-C36 | 27.47 | 0.3 | 0.081 |
| π | C12-C13 | π* | C18-C19 | 25.52 | 0.29 | 0.079 |
| π | C22-C23 | π* | C34-O35 | 24.21 | 0.31 | 0.079 |
| π | C72-C73 | π* | C75-C79 | 23.83 | 0.29 | 0.074 |
| π | C88-C89 | π* | C84-C86 | 22.79 | 0.3 | 0.074 |
| π | C75-C79 | π* | C74-C77 | 21.25 | 0.31 | 0.073 |
| π | C2-C3 | π* | C9-C51 | 20.87 | 0.29 | 0.071 |
| π | C93-C94 | π* | C83-C85 | 19.22 | 0.29 | 0.068 |
| π | C1-C6 | π* | C2-C3 | 18.71 | 0.3 | 0.071 |
| π | C4-C5 | π* | C2-C3 | 17.66 | 0.3 | 0.069 |
| π | C4-C5 | π* | C1-C6 | 16.99 | 0.32 | 0.067 |
| π | C2-C3 | π* | C4-C5 | 15.28 | 0.31 | 0.064 |
| π | C2-C3 | π* | C8-C16 | 14.98 | 0.3 | 0.064 |
| π | C1-C6 | π* | C15-C64 | 12.86 | 0.31 | 0.057 |
| π | C69-C71 | π* | C72-C73 | 11.68 | 0.32 | 0.058 |
| π | C33-C36 | π* | C28-C29 | 8.53 | 0.33 | 0.05 |
| π | C34-O35 | π* | C28-C29 | 4.16 | 0.42 | 0.042 |
| σ | C22-H24 | σ* | C18-S20 | 10.36 | 0.71 | 0.077 |
| σ | C37-N38 | σ* | C36-C37 | 8.01 | 1.57 | 0.101 |
| σ | C12-C13 | σ* | C11-C17 | 7.35 | 1.16 | 0.083 |
| σ | C65-C66 | σ* | C64-C65 | 6.56 | 1.29 | 0.082 |
| σ | C18-C22 | σ* | C22-C23 | 6.49 | 1.35 | 0.083 |
| σ | C11-C12 | σ* | C11-C17 | 6.08 | 1.17 | 0.075 |
| σ | C85-C88 | σ* | C85-C94 | 5.85 | 1.25 | 0.076 |
| σ | C4-H7 | σ* | C5-C6 | 5.68 | 1.06 | 0.07 |
| σ | C26-C27 | σ* | C28-C33 | 5.51 | 1.22 | 0.073 |
| σ | C19-H21 | σ* | C18-S20 | 5.44 | 0.73 | 0.056 |
| σ | C11-C17 | σ* | C11-C12 | 5.39 | 1.2 | 0.072 |
| σ | C22-C23 | σ* | C23-C33 | 5.37 | 1.24 | 0.073 |
| σ | C8-C9 | σ* | C9-C51 | 5.32 | 1.27 | 0.074 |
| σ | C1-C6 | σ* | C1-C2 | 5.29 | 1.28 | 0.074 |
| σ | C83-C85 | σ* | C83-C84 | 5.24 | 1.26 | 0.073 |
| σ | C27-C28 | σ* | C28-C29 | 5.2 | 1.3 | 0.073 |
| σ | C66-S67 | σ* | C6-C15 | 5.16 | 1.2 | 0.07 |
| σ | C94-C96 | σ* | C93-C94 | 5.09 | 1.26 | 0.072 |
| σ | N82-C93 | σ* | C83-C84 | 4.13 | 1.38 | 0.067 |
| σ | C13-C19 | σ* | C11-C12 | 3.15 | 1.32 | 0.058 |
| σ | C23-C33 | σ* | C28-C33 | 2.02 | 1.15 | 0.043 |
| σ | C64-C65 | σ* | C65-S68 | 1.02 | 0.93 | 0.027 |
| σ | C51-O53 | σ* | C55-H58 | 0.52 | 1.28 | 0.023 |
| σ | C65-C66 | σ* | C6-C15 | 0.5 | 1.24 | 0.022 |
| LP(1) | N82 | π* | C83-C85 | 35.44 | 0.31 | 0.096 |
| LP(1) | N40 | σ* | C36-C39 | 12.7 | 1.04 | 0.103 |
| LP(1) | N82 | π* | C75-C79 | 11.56 | 0.3 | 0.053 |
| LP(1) | O54 | π* | C1-C6 | 5.94 | 0.62 | 0.059 |
| LP(1) | F49 | σ* | C25-C26 | 1.01 | 1.59 | 0.036 |
| LP(1) | S20 | σ* | C18-C22 | 0.51 | 1.22 | 0.022 |
| LP(2) | S20 | π* | C12-C13 | 29.33 | 0.25 | 0.079 |
| LP(2) | S14 | π* | C10-C11 | 26.32 | 0.28 | 0.078 |
| LP(2) | S68 | π* | C65-C66 | 24.95 | 0.26 | 0.075 |
| LP(2) | S68 | π* | C69-C71 | 23.84 | 0.28 | 0.073 |
| LP(2) | S14 | π* | C12-C13 | 22.69 | 0.25 | 0.071 |
| LP(2) | O35 | σ* | C29-C34 | 21.3 | 0.76 | 0.115 |
| LP(2) | O35 | σ* | C23-C34 | 18.74 | 0.76 | 0.108 |
| LP(2) | O54 | σ* | C1-C2 | 8.47 | 0.89 | 0.078 |
| LP(2) | F49 | σ* | C25-C26 | 7.67 | 0.98 | 0.078 |
| LP(2) | O54 | σ* | C59-H61 | 6.71 | 0.7 | 0.062 |
| LP(2) | F49 | σ* | C25-C30 | 6.33 | 1.01 | 0.072 |
| LP(2) | O54 | σ* | C59-H60 | 5.4 | 0.7 | 0.055 |
| LP(2) | O35 | σ* | C12-S20 | 2.28 | 0.52 | 0.031 |
| LP(3) | F49 | π* | C25-C30 | 20.51 | 0.46 | 0.093 |
| LP(3) | F50 | π* | C26-C27 | 20.44 | 0.46 | 0.093 |

**Table S15:** Natural bond orbitals representative values for **PCMD2**.

| **Donor(*i*)** | **Type** | **Acceptor(*j*)** | **Type** | ***E*(2)**  **[*kcal/mol*]** | ***E*(*j*)-*E*(*i*)**  **[*a.u.*]** | **F(*i,j*)**  **[*a.u.*]** |
| --- | --- | --- | --- | --- | --- | --- |
| π | C18-C19 | π* | C22-C23 | 31.82 | 0.31 | 0.089 |
| π | C22-C23 | π* | C33-C36 | 27.3 | 0.3 | 0.081 |
| π | C12-C13 | π* | C18-C19 | 25.36 | 0.29 | 0.079 |
| π | C86-C87 | π* | C89-C93 | 23.85 | 0.29 | 0.074 |
| π | C100-C103 | π* | C99-C101 | 22.8 | 0.3 | 0.074 |
| π | C89-C93 | π* | C86-C87 | 21.57 | 0.3 | 0.073 |
| π | C29-C30 | π* | C34-O35 | 20.85 | 0.3 | 0.072 |
| π | C27-C28 | π* | C33-C36 | 19.87 | 0.29 | 0.069 |
| π | C1-C6 | π* | C2-C3 | 18.69 | 0.31 | 0.071 |
| π | C61-C62 | π* | C59-C60 | 18.35 | 0.32 | 0.071 |
| π | C12-C13 | π* | C10-C11 | 17.48 | 0.32 | 0.067 |
| π | C61-C62 | π* | C64-C66 | 16.83 | 0.32 | 0.067 |
| π | C18-C19 | π* | C12-C13 | 15.25 | 0.28 | 0.061 |
| π | C2-C3 | π* | C8-C15 | 14.97 | 0.3 | 0.064 |
| π | C64-C66 | π* | C86-C87 | 12.26 | 0.32 | 0.059 |
| π | C22-C23 | π* | C18-C19 | 11.77 | 0.29 | 0.053 |
| π | C33-C36 | π* | C27-C28 | 9.06 | 0.34 | 0.05 |
| π | C33-C36 | π* | C22-C23 | 8.08 | 0.33 | 0.047 |
| π | C34-O35 | π* | C29-C30 | 4.13 | 0.44 | 0.041 |
| π | C34-O35 | π* | C22-C23 | 3.67 | 0.43 | 0.038 |
| π | C33-C36 | π* | C33-C36 | 2.13 | 0.32 | 0.024 |
| π | C89-C93 | σ* | N96-C97 | 1.76 | 0.71 | 0.034 |
| π | C107-C108 | π* | C107-C108 | 0.67 | 0.29 | 0.013 |
| σ | C22-H24 | σ* | C18-S20 | 10.42 | 0.71 | 0.077 |
| σ | C36-C37 | σ* | C37-N38 | 8.12 | 1.61 | 0.103 |
| σ | C61-C62 | σ* | C16-C60 | 7.17 | 1.16 | 0.082 |
| σ | C22-H24 | σ* | C23-C34 | 6.78 | 0.99 | 0.074 |
| σ | S65-C66 | σ* | C60-C61 | 5.09 | 1.23 | 0.071 |
| σ | C97-C98 | σ* | C98-C100 | 4.35 | 1.27 | 0.067 |
| σ | C80-C84 | σ* | C82-C84 | 3.16 | 1.29 | 0.057 |
| σ | C64-H67 | σ* | C61-C62 | 2.69 | 1.08 | 0.048 |
| σ | C86-C87 | σ* | C87-H90 | 1.27 | 1.12 | 0.034 |
| σ | C62-C64 | σ* | C62-S63 | 0.5 | 0.91 | 0.019 |
| LP(1) | N96 | π* | C107-C108 | 35.42 | 0.31 | 0.096 |
| LP(1) | N40 | σ* | C36-C39 | 12.64 | 1.04 | 0.103 |
| LP(1) | N96 | π* | C89-C93 | 11.94 | 0.3 | 0.054 |
| LP(1) | O69 | π* | C9-C57 | 6.33 | 0.62 | 0.061 |
| LP(1) | O68 | π* | C1-C6 | 5.9 | 0.62 | 0.059 |
| LP(1) | N96 | σ* | C89-C93 | 4.16 | 0.85 | 0.058 |
| LP(1) | S20 | σ* | C12-C13 | 3.33 | 1.18 | 0.056 |
| LP(1) | S20 | σ* | C18-C19 | 2.23 | 1.2 | 0.046 |
| LP(1) | O68 | σ* | C70-H71 | 1.15 | 0.94 | 0.03 |
| LP(1) | S20 | σ* | C18-C22 | 0.52 | 1.22 | 0.022 |
| LP(2) | S20 | π* | C12-C13 | 29.46 | 0.24 | 0.079 |
| LP(2) | S14 | π* | C10-C11 | 26.21 | 0.28 | 0.077 |
| LP(2) | S65 | π* | C61-C62 | 24.93 | 0.26 | 0.075 |
| LP(2) | S65 | π* | C64-C66 | 23.79 | 0.28 | 0.073 |
| LP(2) | S14 | π* | C12-C13 | 22.61 | 0.25 | 0.071 |
| LP(2) | S20 | π* | C18-C19 | 21.82 | 0.26 | 0.068 |
| LP(2) | O35 | σ* | C29-C34 | 20.67 | 0.77 | 0.114 |
| LP(2) | O35 | σ* | C23-C34 | 18.65 | 0.75 | 0.107 |
| LP(2) | O68 | σ* | C1-C2 | 8.53 | 0.89 | 0.078 |
| LP(2) | O69 | σ* | C3-C57 | 7.52 | 0.89 | 0.074 |
| LP(2) | O69 | σ* | C74-H75 | 6.16 | 0.7 | 0.059 |
| LP(2) | O68 | σ* | C70-H71 | 5.27 | 0.7 | 0.055 |
| LP(2) | O35 | σ* | C12-S20 | 2.45 | 0.52 | 0.032 |

**Table S16:** Natural bond orbitals representative values for **PITPCMD3**.

| **Donor(*i*)** | **Type** | **Acceptor(*j*)** | **Type** | ***E*(2)**  **[*kcal/mol*]** | ***E*(*j*)-*E*(*i*)**  **[*a.u.*]** | **F(*i,j*)**  **[*a.u.*]** |
| --- | --- | --- | --- | --- | --- | --- |
| π | C18-C19 | π* | C22-C23 | 32.24 | 0.31 | 0.09 |
| π | C22-C23 | π* | C33-C36 | 27.53 | 0.3 | 0.081 |
| π | C12-C13 | π* | C18-C19 | 25.6 | 0.29 | 0.079 |
| π | C22-C23 | π* | C34-O35 | 24.2 | 0.31 | 0.079 |
| π | C98-C101 | π* | C97-C99 | 22.79 | 0.3 | 0.074 |
| π | C105-C106 | π* | C108-C111 | 22.45 | 0.3 | 0.075 |
| π | C86-C89 | π* | C87-C91 | 21.94 | 0.3 | 0.073 |
| π | C86-C89 | π* | C84-C85 | 21.04 | 0.3 | 0.072 |
| π | C2-C3 | π* | C9-C57 | 20.85 | 0.29 | 0.071 |
| π | C59-C60 | π* | C1-C6 | 19.8 | 0.33 | 0.074 |
| π | C95-C96 | π* | C97-C99 | 19.67 | 0.29 | 0.069 |
| π | C105-C106 | π* | C107-C109 | 19.65 | 0.29 | 0.069 |
| π | C105-C106 | π* | C95-C96 | 19.22 | 0.29 | 0.068 |
| π | C10-C11 | π* | C12-C13 | 19.01 | 0.28 | 0.07 |
| π | C107-C109 | π* | C108-C111 | 18.66 | 0.3 | 0.068 |
| π | C59-C60 | π* | C1-C6 | 19.8 | 0.33 | 0.074 |
| π | C95-C96 | π* | C97-C99 | 19.67 | 0.29 | 0.069 |
| π | C2-C3 | π* | C1-C6 | 18.6 | 0.3 | 0.068 |
| π | C9-C57 | π* | C8-C15 | 18.03 | 0.33 | 0.07 |
| π | C25-C26 | π* | C80-C83 | 17.89 | 0.28 | 0.066 |
| π | C80-C83 | π* | C25-C26 | 17.37 | 0.32 | 0.07 |
| π | C25-C26 | π* | C29-C30 | 17.14 | 0.3 | 0.068 |
| π | C4-C5 | π* | C1-C6 | 17 | 0.32 | 0.067 |
| π | C78-C82 | π* | C25-C26 | 16.97 | 0.32 | 0.07 |
| π | C61-C62 | π* | C64-C66 | 16.84 | 0.32 | 0.067 |
| π | C8-C15 | π* | C9-C57 | 16.7 | 0.31 | 0.066 |
| π | C2-C3 | π* | C4-C5 | 15.27 | 0.31 | 0.064 |
| π | C84-C85 | π* | C64-C66 | 14.79 | 0.29 | 0.059 |
| π | C1-C6 | π* | C59-C60 | 12.88 | 0.31 | 0.057 |
| π | C34-O35 | π* | C29-C30 | 4.16 | 0.44 | 0.041 |
| π | C34-O35 | π* | C22-C23 | 3.66 | 0.43 | 0.038 |
| π | C22-C23 | π* | C22-C23 | 2.81 | 0.31 | 0.026 |
| π | C37-N38 | σ* | C22-H24 | 1.72 | 0.77 | 0.033 |
| π | C1-C6 | σ* | C70-H72 | 0.53 | 0.65 | 0.018 |
| π | C80-C83 | π* | C80-C83 | 0.51 | 0.32 | 0.012 |
| σ | C22-H24 | σ* | C18-S20 | 10.43 | 0.71 | 0.077 |
| σ | C59-C60 | σ* | C61-S65 | 8.47 | 0.92 | 0.079 |
| σ | C37-N38 | σ* | C36-C37 | 7.99 | 1.57 | 0.101 |
| σ | C64-C66 | σ* | C62-S63 | 7.39 | 0.94 | 0.074 |
| σ | C12-C13 | σ* | C11-C17 | 7.35 | 1.16 | 0.083 |
| σ | C61-C62 | σ* | C16-C60 | 7.17 | 1.16 | 0.082 |
| σ | C22-H24 | σ* | C23-C34 | 6.74 | 0.99 | 0.073 |
| σ | C61-C62 | σ* | C60-C61 | 6.55 | 1.29 | 0.082 |
| σ | C18-C22 | σ* | C22-C23 | 6.47 | 1.34 | 0.083 |
| σ | C107-C109 | σ* | N94-C105 | 6.46 | 1.14 | 0.077 |
| σ | C9-C57 | σ* | C9-C10 | 6.43 | 1.25 | 0.08 |
| σ | C1-C6 | σ* | C6-C59 | 6.39 | 1.25 | 0.08 |
| σ | C6-C59 | σ* | C1-C6 | 6.33 | 1.3 | 0.081 |
| σ | C1-C6 | σ* | C5-C6 | 6.24 | 1.28 | 0.08 |
| σ | C33-C36 | σ* | C36-C37 | 6.2 | 1.27 | 0.079 |
| σ | C36-C37 | σ* | C33-C36 | 6.17 | 1.33 | 0.081 |
| σ | C96-C98 | σ* | C96-C106 | 5.85 | 1.25 | 0.076 |
| σ | C8-C17 | σ* | C11-C12 | 5.79 | 1.19 | 0.074 |
| σ | C3-C57 | σ* | C9-C57 | 5.52 | 1.31 | 0.076 |
| σ | C27-C28 | σ* | C28-C29 | 5.42 | 1.28 | 0.075 |
| σ | C11-C17 | σ* | C11-C12 | 5.39 | 1.2 | 0.072 |
| σ | C1-C2 | σ* | C2-C3 | 4.01 | 1.24 | 0.063 |
| σ | C18-S20 | σ* | C19-H21 | 3.28 | 1.06 | 0.053 |
| σ | C8-C17 | σ* | C8-C9 | 2.13 | 1.16 | 0.044 |
| σ | C84-C86 | σ* | C86-H90 | 1.21 | 1.12 | 0.033 |
| σ | C89-C91 | σ* | N94-C105 | 0.52 | 1.16 | 0.022 |
| σ | C61-C62 | σ* | C6-C59 | 0.5 | 1.24 | 0.022 |
| LP(1) | N94 | π* | C87-C91 | 12.03 | 0.3 | 0.054 |
| LP(1) | S20 | σ* | C12-C13 | 3.34 | 1.18 | 0.056 |
| LP(1) | N94 | σ* | C87-C91 | 4.16 | 0.85 | 0.058 |
| LP(1) | S20 | σ* | C18-C19 | 2.22 | 1.2 | 0.046 |
| LP(1) | O68 | σ* | C70-H71 | 1.21 | 0.94 | 0.03 |
| LP(1) | O69 | σ* | C9-C57 | 0.52 | 1.19 | 0.022 |
| LP(2) | S20 | π* | C12-C13 | 29.54 | 0.24 | 0.079 |
| LP(2) | S65 | π* | C64-C66 | 23.78 | 0.28 | 0.073 |
| LP(2) | S14 | π* | C12-C13 | 22.58 | 0.25 | 0.07 |
| LP(2) | O35 | σ* | C29-C34 | 20.8 | 0.76 | 0.114 |
| LP(2) | O68 | σ* | C1-C2 | 8.57 | 0.89 | 0.079 |
| LP(2) | O69 | σ* | C9-C57 | 7.72 | 0.94 | 0.077 |
| LP(2) | F116 | σ* | C80-C83 | 6.02 | 1.04 | 0.071 |
| LP(2) | O68 | σ* | C70-H71 | 5.18 | 0.7 | 0.054 |
| LP(2) | O35 | σ* | C12-S20 | 2.46 | 0.52 | 0.032 |
| LP(3) | F116 | π* | C80-C83 | 21.21 | 0.47 | 0.094 |
| LP(3) | F115 | π* | C78-C82 | 21.1 | 0.47 | 0.094 |

**Table S17:** Natural bond orbitals representative values for **PCMD4**.

| **Donor(*i*)** | **Type** | **Acceptor(*j*)** | **Type** | ***E*(2)**  **[*kcal/mol*]** | ***E*(*j*)-*E*(*i*)**  **[*a.u.*]** | **F(*i,j*)**  **[*a.u.*]** |
| --- | --- | --- | --- | --- | --- | --- |
| π | C18-C19 | π* | C22-C23 | 32.6 | 0.31 | 0.09 |
| π | C22-C23 | π* | C33-C36 | 27.76 | 0.3 | 0.081 |
| π | C12-C13 | π* | C18-C19 | 25.83 | 0.29 | 0.079 |
| π | C22-C23 | π* | C34-O35 | 24.41 | 0.31 | 0.079 |
| π | C84-C85 | π* | C87-C91 | 23.86 | 0.29 | 0.074 |
| π | C107-C109 | π* | C105-C106 | 22.35 | 0.3 | 0.076 |
| π | C87-C91 | π* | C86-C89 | 21.21 | 0.31 | 0.073 |
| π | C29-C30 | π* | C34-O35 | 20.16 | 0.31 | 0.071 |
| π | C27-C28 | π* | C33-C36 | 19.03 | 0.3 | 0.067 |
| π | C61-C62 | π* | C59-C60 | 18.39 | 0.32 | 0.071 |
| π | C27-C28 | π* | C25-C26 | 17.33 | 0.3 | 0.067 |
| π | C80-C83 | π* | C25-C26 | 16.02 | 0.32 | 0.068 |
| π | C59-C60 | π* | C61-C62 | 15.24 | 0.29 | 0.064 |
| π | C84-C85 | π* | C64-C66 | 14.9 | 0.29 | 0.059 |
| π | C64-C66 | π* | C84-C85 | 12.37 | 0.32 | 0.059 |
| π | C22-C23 | π* | C18-C19 | 11.79 | 0.29 | 0.053 |
| π | C33-C36 | π* | C27-C28 | 9.25 | 0.34 | 0.05 |
| π | C33-C36 | π* | C22-C23 | 8.11 | 0.33 | 0.047 |
| π | C34-O35 | π* | C29-C30 | 4.19 | 0.44 | 0.041 |
| π | C34-O35 | π* | C22-C23 | 3.65 | 0.43 | 0.038 |
| π | C33-C36 | π* | C33-C36 | 2.05 | 0.32 | 0.023 |
| π | C105-C106 | π* | C105-C106 | 0.67 | 0.29 | 0.013 |
| σ | C22-H24 | σ* | C18-S20 | 10.48 | 0.71 | 0.077 |
| σ | C39-N40 | σ* | C36-C39 | 8.08 | 1.57 | 0.101 |
| σ | C61-C62 | σ* | C16-C60 | 7.17 | 1.16 | 0.082 |
| σ | C33-C36 | σ* | C36-C39 | 6.07 | 1.27 | 0.078 |
| σ | C108-C111 | σ* | C96-C106 | 5.11 | 1.25 | 0.071 |
| σ | C30-H32 | σ* | C25-C26 | 4.52 | 1.07 | 0.062 |
| σ | C5-C6 | σ* | C4-H7 | 3.21 | 1.08 | 0.053 |
| σ | C29-C34 | σ* | C28-C29 | 2.47 | 1.21 | 0.049 |
| σ | C2-C15 | σ* | C15-H58 | 1.14 | 1.1 | 0.032 |
| σ | C61-C62 | σ* | C6-C59 | 0.5 | 1.24 | 0.022 |
| LP(1) | N38 | σ* | C36-C37 | 12.65 | 1.04 | 0.103 |
| LP(1) | N38 | σ* | C36-C37 | 12.65 | 1.04 | 0.103 |
| LP(1) | N94 | π* | C87-C91 | 11.91 | 0.3 | 0.054 |
| LP(1) | N94 | σ* | C87-C91 | 4.19 | 0.85 | 0.058 |
| LP(1) | S20 | σ* | C12-C13 | 3.33 | 1.18 | 0.056 |
| LP(1) | O68 | σ* | C70-H71 | 1.24 | 0.94 | 0.031 |
| LP(1) | O68 | σ* | C70-H71 | 1.24 | 0.94 | 0.031 |
| LP(1) | O69 | σ* | C9-C57 | 0.64 | 1.19 | 0.025 |
| LP(1) | S20 | σ* | C18-C22 | 0.52 | 1.22 | 0.023 |
| LP(2) | S20 | π* | C12-C13 | 29.56 | 0.24 | 0.079 |
| LP(2) | S14 | π* | C10-C11 | 26.44 | 0.28 | 0.078 |
| LP(2) | S63 | π* | C59-C60 | 24.26 | 0.29 | 0.075 |
| LP(2) | S65 | π* | C64-C66 | 23.78 | 0.28 | 0.073 |
| LP(2) | S14 | π* | C12-C13 | 22.61 | 0.25 | 0.07 |
| LP(2) | S20 | π* | C18-C19 | 21.65 | 0.26 | 0.068 |
| LP(2) | O35 | σ* | C23-C34 | 18.57 | 0.76 | 0.107 |
| LP(2) | O69 | σ* | C3-C57 | 7.72 | 0.89 | 0.075 |
| LP(2) | O69 | σ* | C9-C57 | 7.59 | 0.94 | 0.076 |
| LP(2) | O69 | σ* | C74-H75 | 6.05 | 0.7 | 0.059 |
| LP(2) | Cl116 | σ* | C82-C83 | 5.53 | 0.85 | 0.061 |
| LP(2) | O68 | σ* | C70-H71 | 5.12 | 0.7 | 0.054 |
| LP(2) | Cl115 | σ* | C78-C82 | 4.27 | 0.92 | 0.056 |
| LP(2) | Cl115 | σ* | C26-C78 | 0.55 | 0.89 | 0.02 |
| LP(3) | Cl116 | π* | C80-C83 | 15.01 | 0.35 | 0.068 |
| LP(3) | Cl115 | π* | C78-C82 | 14.92 | 0.35 | 0.068 |

**Table S18:** Natural bond orbitals representative values for **PCMD5**.

| **Donor(*i*)** | **Type** | **Acceptor(*j*)** | **Type** | ***E*(2)**  **[*kcal/mol*]** | ***E*(*j*)-*E*(*i*)**  **[*a.u.*]** | **F(*i,j*)**  **[*a.u.*]** |
| --- | --- | --- | --- | --- | --- | --- |
| C12-C13 | σ | C13-C19 | σ* | 4.92 | 1.3 | 0.072 |
| C27-C28 | σ | C28-C33 | σ* | 4.87 | 1.21 | 0.069 |
| C89-H93 | σ | C84-C86 | σ* | 4.27 | 1.09 | 0.061 |
| C2-C15 | σ | C2-C3 | σ* | 4.04 | 1.23 | 0.063 |
| C3-C4 | σ | C3-C57 | σ* | 3.98 | 1.24 | 0.063 |
| C2-C3 | σ | C57-O69 | σ* | 3.79 | 1.04 | 0.056 |
| C25-C80 | σ | C25-C30 | σ* | 3.63 | 1.27 | 0.061 |
| C61-S65 | σ | C66-C84 | σ* | 3.26 | 1.2 | 0.056 |
| C25-C80 | σ | C26-C27 | σ* | 3.01 | 1.27 | 0.055 |
| N94-C95 | σ | C91-N94 | σ* | 2.81 | 1.2 | 0.052 |
| C25-C26 | σ | C80-H81 | σ* | 2.01 | 1.1 | 0.042 |
| C26-C78 | σ | C27-C28 | σ* | 1.99 | 1.32 | 0.046 |
| C1-C6 | σ | O68-C70 | σ* | 1.1 | 1.03 | 0.03 |
| C59-S63 | σ | C59-C60 | σ* | 0.99 | 1.26 | 0.032 |
| C10-C11 | σ | C17-C41 | σ* | 0.81 | 1.11 | 0.027 |
| O68-C70 | σ | C1-C2 | σ* | 0.79 | 1.37 | 0.03 |
| C1-C6 | σ | C59-C60 | σ* | 0.64 | 1.31 | 0.026 |
| C59-C60 | σ | C59-S63 | σ* | 0.57 | 0.92 | 0.02 |
| C1-O68 | σ | C70-H72 | σ* | 0.54 | 1.28 | 0.024 |
| C62-C64 | σ | C62-S63 | σ* | 0.5 | 0.92 | 0.019 |
| C28-C33 | σ | C22-C23 | σ* | 4.24 | 1.27 | 0.066 |
| S65-C66 | σ | C64-H67 | σ* | 4.02 | 1.06 | 0.059 |
| C1- C2 | σ | C2-C3 | σ* | 4 | 1.24 | 0.063 |
| C18-C19 | π | C22-C23 | π* | 34.13 | 0.31 | 0.092 |
| C22-C23 | π | C33-C36 | π* | 28.51 | 0.3 | 0.082 |
| C12-C13 | π | C18-C19 | π* | 26.68 | 0.29 | 0.08 |
| C84-C85 | π | C87-C91 | π* | 23.7 | 0.29 | 0.074 |
| C86-C89 | π | C87-C91 | π* | 21.96 | 0.3 | 0.073 |
| C84-C85 | π | C86-C89 | π* | 20.78 | 0.3 | 0.071 |
| C59-C60 | π | C1-C6 | π* | 19.97 | 0.33 | 0.075 |
| C80-C83 | π | C78-C82 | π* | 16.82 | 0.33 | 0.067 |
| C9-C57 | π | C10-C11 | π* | 15.71 | 0.3 | 0.062 |
| C1-C6 | π | C59-C60 | π* | 12.83 | 0.31 | 0.057 |
| C33-C36 | π | C22-C23 | π* | 8.09 | 0.33 | 0.047 |
| C34-O35 | π | C29-C30 | π* | 4.25 | 0.44 | 0.041 |
| C22-C23 | π | C22-C23 | π* | 3.02 | 0.31 | 0.027 |
| C33-C36 | π | C33-C36 | π* | 1.87 | 0.32 | 0.022 |
| C10-C11 | π | C10-C11 | π* | 0.89 | 0.31 | 0.015 |
| C39-N40 | π | C37-N38 | π* | 0.8 | 0.47 | 0.017 |
| C3 | LP(1) | C9-C57 | π* | 69.77 | 0.15 | 0.11 |
| C2 | LP(1) | C1-C6 | π* | 62.23 | 0.16 | 0.107 |
| N40 | LP(1) | C36-C39 | σ* | 12.7 | 1.04 | 0.103 |
| N94 | LP(1) | C89-C91 | σ* | 4.17 | 0.85 | 0.058 |
| O35 | LP(1) | C23-C34 | σ* | 3.51 | 1.18 | 0.058 |
| S20 | LP(1) | C12-C13 | σ* | 3.26 | 1.18 | 0.055 |
| S63 | LP(1) | C59-C60 | σ* | 2.8 | 1.24 | 0.053 |
| O68 | LP(1) | C70-H72 | σ* | 2.72 | 0.96 | 0.046 |
| O35 | LP(1) | C29-C34 | σ* | 1.61 | 1.17 | 0.039 |
| S65 | LP(2) | C61-C62 | π* | 24.87 | 0.26 | 0.075 |
| S14 | LP(2) | C12-C13 | π* | 22.74 | 0.25 | 0.07 |
| O121 | LP(2) | C80-C83 | π* | 1.1 | 0.39 | 0.02 |
| O35 | LP(2) | C29-C34 | σ* | 21.37 | 0.75 | 0.115 |
| O35 | LP(2) | C23-C34 | σ* | 18.58 | 0.76 | 0.108 |
| O68 | LP(2) | C1-C2 | σ* | 8.44 | 0.89 | 0.078 |
| O68 | LP(2) | C70-H73 | σ* | 6.72 | 0.7 | 0.062 |
| O69 | LP(2) | C74-H75 | σ* | 5.8 | 0.7 | 0.057 |
| O35 | LP(2) | C12-S20 | σ* | 2.3 | 0.52 | 0.031 |
| S20 | LP(2) | C18-C19 | π* | 21.42 | 0.26 | 0.067 |
| O122 | LP(2) | C78-C82 | π* | 1.1 | 0.38 | 0.019 |
| O119 | LP(3) | C78-C82 | π* | 0.93 | 0.33 | 0.016 |
| O119 | LP(3) | S118-O120 | σ* | 11.52 | 0.63 | 0.078 |
| O120 | LP(3) | S118-O119 | σ* | 3.72 | 0.63 | 0.044 |
| O119 | LP(3) | S118-O122 | σ* | 29.05 | 0.45 | 0.103 |
| O117 | LP(3) | S115-O121 | σ* | 27.4 | 0.44 | 0.1 |
| O116 | LP(3) | S115-O121 | σ* | 22.79 | 0.45 | 0.092 |

**Table S19:** Natural bond orbitals representative values for **PITPCMD6**.

| **Donor(*i*)** | **Type** | **Acceptor(*j*)** | **Type** | ***E*(2)**  **[*kcal/mol*]** | ***E*(*j*)-*E*(*i*)**  **[*a.u.*]** | **F(*i,j*)**  **[*a.u.*]** |
| --- | --- | --- | --- | --- | --- | --- |
| C3-C4 | σ | C4-C5 | σ* | 4.99 | 1.33 | 0.073 |
| C28-C29 | σ | C27-C28 | σ* | 4.81 | 1.29 | 0.071 |
| C87-H92 | σ | C89-C91 | σ* | 4.67 | 1.1 | 0.064 |
| C1-C2 | σ | C2-C3 | σ* | 4.02 | 1.24 | 0.063 |
| C3-C4 | σ | C3-C57 | σ* | 3.97 | 1.24 | 0.063 |
| C28-C29 | σ | C33-C36 | σ* | 3.74 | 1.27 | 0.062 |
| C5-C6 | σ | C4-H7 | σ* | 3.22 | 1.08 | 0.053 |
| C13-C19 | σ | C18-C19 | σ* | 3.19 | 1.28 | 0.057 |
| C60-C61 | σ | C6-C59 | σ* | 3.02 | 1.24 | 0.055 |
| C27-C28 | σ | C26-C78 | σ* | 2.92 | 1.29 | 0.055 |
| C25-C26 | σ | C78-H79 | σ* | 2 | 1.11 | 0.042 |
| C66-C84 | σ | C62-C64 | σ* | 1.99 | 1.25 | 0.044 |
| C33-C36 | σ | C28-C29 | σ* | 1.1 | 1.3 | 0.034 |
| C27-C28 | σ | C23-C33 | σ* | 0.99 | 1.24 | 0.031 |
| C59-C60 | σ | C16-C49 | σ* | 0.87 | 1.1 | 0.028 |
| O69-C74 | σ | C9-C57 | σ* | 0.79 | 1.43 | 0.03 |
| C18-S20 | σ | C13-S14 | σ* | 0.66 | 0.85 | 0.021 |
| C59-C60 | σ | C59-S63 | σ* | 0.57 | 0.92 | 0.02 |
| C6-C59 | σ | C59-S63 | σ* | 0.53 | 0.9 | 0.02 |
| C62-C64 | σ | C62-S63 | σ* | 0.5 | 0.92 | 0.019 |
| C66-C84 | σ | C84-C85 | σ* | 4.24 | 1.27 | 0.065 |
| C3-C57 | σ | C2-C3 | σ* | 4.08 | 1.24 | 0.064 |
| C18-C22 | σ | C18-C19 | σ* | 4 | 1.28 | 0.064 |
| C18-C19 | π | C22-C23 | π* | 33.46 | 0.31 | 0.091 |
| C22-C23 | π | C33-C36 | π* | 28.3 | 0.3 | 0.082 |
| C12-C13 | π | C18-C19 | π* | 26.31 | 0.29 | 0.08 |
| C84-C85 | π | C87-C91 | π* | 23.71 | 0.29 | 0.074 |
| C87-C91 | π | C84-C85 | π* | 21.64 | 0.31 | 0.073 |
| C84-C85 | π | C86-C89 | π* | 20.78 | 0.3 | 0.071 |
| C95-C96 | π | C97-C99 | π* | 19.67 | 0.29 | 0.069 |
| C25-C26 | π | C27-C28 | π* | 16.75 | 0.3 | 0.067 |
| C78-C82 | π | C25-C26 | π* | 15.85 | 0.31 | 0.066 |
| C1-C6 | π | C59-C60 | π* | 12.87 | 0.31 | 0.057 |
| C33-C36 | π | C22-C23 | π* | 8.14 | 0.33 | 0.047 |
| C34-O35 | π | C29-C30 | π* | 4.22 | 0.44 | 0.041 |
| C34-O35 | π | C22-C23 | π* | 3.64 | 0.43 | 0.038 |
| C33-C36 | π | C33-C36 | π* | 1.97 | 0.32 | 0.023 |
| C37-N38 | π | C39-N40 | π* | 0.79 | 0.47 | 0.017 |
| C95-C96 | π | C95-C96 | π* | 0.67 | 0.29 | 0.013 |
| N94 | LP(1) | C95-C96 | π* | 35.34 | 0.31 | 0.096 |
| N94 | LP(1) | C87-C91 | π* | 12.35 | 0.3 | 0.054 |
| N40 | LP(1) | C36-C39 | σ* | 12.68 | 1.04 | 0.103 |
| N94 | LP(1) | C89-C91 | σ* | 4.27 | 0.85 | 0.059 |
| N94 | LP(1) | C87-C91 | σ* | 4.07 | 0.85 | 0.057 |
| O35 | LP(1) | C23-C34 | σ* | 3.54 | 1.18 | 0.058 |
| S20 | LP(1) | C12-C13 | σ* | 3.32 | 1.18 | 0.056 |
| S14 | LP(1) | C10-C11 | σ* | 2.99 | 1.23 | 0.054 |
| O35 | LP(1) | C29-C34 | σ* | 1.58 | 1.18 | 0.039 |
| S20 | LP(2) | C12-C13 | π* | 29.58 | 0.24 | 0.079 |
| S65 | LP(2) | C61-C62 | π* | 24.8 | 0.26 | 0.075 |
| F120 | LP(2) | C78-C82 | π* | 0.52 | 0.48 | 0.015 |
| O35 | LP(2) | C29-C34 | σ* | 21.11 | 0.76 | 0.114 |
| O35 | LP(2) | C23-C34 | σ* | 18.55 | 0.76 | 0.107 |
| O69 | LP(2) | C9-C57 | σ* | 7.64 | 0.94 | 0.076 |
| O68 | LP(2) | C1-C6 | σ* | 6.97 | 0.94 | 0.073 |
| O68 | LP(2) | C70-H71 | σ* | 5.59 | 0.69 | 0.056 |
| O35 | LP(2) | C12-S20 | σ* | 2.41 | 0.52 | 0.032 |
| S14 | LP(2) | C10-C11 | π* | 26.65 | 0.28 | 0.078 |
| F119 | LP(2) | C80-C83 | π* | 0.51 | 0.48 | 0.015 |
| F121 | LP(3) | C116-F120 | σ* | 11.69 | 0.72 | 0.082 |
| F120 | LP(3) | C116-F122 | σ* | 10.69 | 0.72 | 0.082 |
| F121 | LP(3) | C116-F122 | σ* | 9.93 | 0.72 | 0.076 |

**Table S20:** Natural bond orbitals representative values for **PCMD7**.

| **Donor(*i*)** | **Type** | **Acceptor(*j*)** | **Type** | ***E*(2)**  **[*kcal/mol*]** | ***E*(*j*)-*E*(*i*)**  **[*a.u.*]** | **F(*i,j*)**  **[*a.u.*]** |
| --- | --- | --- | --- | --- | --- | --- |
| π | C18-C19 | π* | C22-C23 | 32.84 | 0.31 | 0.091 |
| π | C22-C23 | π* | C33-C36 | 27.96 | 0.3 | 0.082 |
| π | C12-C13 | π* | C18-C19 | 25.93 | 0.29 | 0.079 |
| π | C22-C23 | π* | C34-O35 | 24.4 | 0.31 | 0.079 |
| π | C84-C85 | π* | C87-C91 | 23.86 | 0.29 | 0.074 |
| π | C98-C101 | π* | C97-C99 | 22.78 | 0.3 | 0.074 |
| π | C86-C89 | π* | C87-C91 | 21.95 | 0.3 | 0.073 |
| π | C2-C3 | π* | C9-C57 | 20.96 | 0.29 | 0.071 |
| π | C29-C30 | π* | C34-O35 | 19.86 | 0.31 | 0.071 |
| π | C29-C30 | π* | C27-C28 | 18.44 | 0.31 | 0.068 |
| π | C12-C13 | π* | C10-C11 | 17.27 | 0.32 | 0.066 |
| π | C8-C15 | π* | C9-C57 | 16.74 | 0.31 | 0.066 |
| π | C64-C66 | π* | C61-C62 | 15.28 | 0.29 | 0.065 |
| π | C78-C82 | π* | C115-O116 | 14.33 | 0.33 | 0.062 |
| π | C64-C66 | π* | C84-C85 | 12.36 | 0.32 | 0.059 |
| π | C22-C23 | π* | C18-C19 | 11.77 | 0.29 | 0.053 |
| π | C33-C36 | π* | C27-C28 | 9.26 | 0.34 | 0.05 |
| π | C33-C36 | π* | C22-C23 | 8.12 | 0.33 | 0.047 |
| π | C34-O35 | π* | C29-C30 | 4.18 | 0.44 | 0.041 |
| π | C34-O35 | π* | C22-C23 | 3.67 | 0.43 | 0.038 |
| π | C33-C36 | π* | C33-C36 | 2.02 | 0.32 | 0.023 |
| π | C37-N38 | σ* | C22-H24 | 1.75 | 0.77 | 0.033 |
| π | C22-C23 | π* | C22-C23 | 2.89 | 0.31 | 0.027 |
| π | C95-C96 | π* | C95-C96 | 0.67 | 0.29 | 0.013 |
| σ | C22-H24 | σ* | C18-S20 | 10.54 | 0.71 | 0.077 |
| σ | C59-C60 | σ* | C61-S65 | 8.46 | 0.92 | 0.079 |
| σ | C61-C62 | σ* | C16-C60 | 7.16 | 1.16 | 0.082 |
| σ | C11-C12 | σ* | C11-C17 | 6.04 | 1.16 | 0.075 |
| σ | C82-C83 | σ* | C78-C82 | 5.11 | 1.31 | 0.073 |
| σ | C18-C19 | σ* | C18-C22 | 4.41 | 1.28 | 0.067 |
| σ | C61-S65 | σ* | C66-C84 | 3.25 | 1.2 | 0.056 |
| σ | C87-C91 | σ* | C85-H88 | 2.31 | 1.13 | 0.046 |
| σ | C10-S14 | σ* | C8-C9 | 1.55 | 1.24 | 0.039 |
| σ | C61-C62 | σ* | C6-C59 | 0.5 | 1.24 | 0.022 |
| LP(1) | N94 | π* | C95-C96 | 35.39 | 0.31 | 0.096 |
| LP(1) | N94 | π* | C87-C91 | 12.03 | 0.3 | 0.054 |
| LP(1) | O117 | σ* | C115-O116 | 7.27 | 1.18 | 0.083 |
| LP(1) | O69 | π* | C9-C57 | 6.42 | 0.62 | 0.061 |
| LP(1) | N94 | σ* | C89-C91 | 4.27 | 0.85 | 0.059 |
| LP(1) | S14 | σ* | C10-C11 | 3.01 | 1.23 | 0.054 |
| LP(1) | S20 | σ* | C18-C19 | 2.23 | 1.2 | 0.046 |
| LP(1) | O119 | σ* | C118-O120 | 1.54 | 1.1 | 0.038 |
| LP(1) | S20 | σ* | C18-C22 | 0.54 | 1.22 | 0.023 |
| LP(2) | O120 | π* | C118-O119 | 47.48 | 0.39 | 0.122 |
| LP(2) | O117 | π* | C115-O116 | 46.97 | 0.39 | 0.121 |
| LP(2) | O116 | σ* | C115-O117 | 33.38 | 0.67 | 0.135 |
| LP(2) | S20 | π* | C12-C13 | 29.3 | 0.24 | 0.079 |
| LP(2) | S14 | π* | C10-C11 | 26.7 | 0.28 | 0.078 |
| LP(2) | S63 | π* | C59-C60 | 24.21 | 0.29 | 0.075 |
| LP(2) | S65 | π* | C64-C66 | 23.81 | 0.28 | 0.073 |
| LP(2) | S14 | π* | C12-C13 | 22.81 | 0.25 | 0.071 |
| LP(2) | S20 | π* | C18-C19 | 21.64 | 0.26 | 0.068 |
| LP(2) | O35 | σ* | C29-C34 | 20.98 | 0.76 | 0.114 |
| LP(2) | O116 | σ* | C82-C115 | 19.19 | 0.71 | 0.107 |
| LP(2) | O35 | σ* | C23-C34 | 18.61 | 0.76 | 0.107 |
| LP(2) | O68 | σ* | C1-C2 | 8.55 | 0.89 | 0.078 |
| LP(2) | O69 | σ* | C9-C57 | 7.26 | 0.94 | 0.074 |
| LP(2) | O68 | σ* | C1-C6 | 6.43 | 0.95 | 0.07 |
| LP(2) | O68 | σ* | C70-H71 | 5.18 | 0.7 | 0.054 |
| LP(2) | O117 | σ* | C125-H128 | 4.79 | 0.72 | 0.055 |
| LP(2) | O35 | σ* | C12-S20 | 2.37 | 0.51 | 0.032 |
| LP(2) | O120 | σ* | C118-O119 | 0.63 | 0.94 | 0.023 |

**Table S21:** Natural bond orbitals representative values for **PCMD8**.

| **Donor(*i*)** | **Type** | **Acceptor(*j*)** | **Type** | ***E*(2)**  **[*kcal/mol*]** | ***E*(*j*)-*E*(*i*)**  **[*a.u.*]** | **F(*i,j*)**  **[*a.u.*]** |
| --- | --- | --- | --- | --- | --- | --- |
| C3-C4 | σ | C4-C5 | σ* | 4.99 | 1.33 | 0.073 |
| C27-C28 | σ | C28-C33 | σ* | 4.86 | 1.21 | 0.069 |
| C89-H93 | σ | C84-C86 | σ* | 4.27 | 1.09 | 0.061 |
| C3-C57 | σ | C2-C3 | σ* | 4.09 | 1.24 | 0.064 |
| C25-C26 | σ | C26-C27 | σ* | 3.99 | 1.25 | 0.063 |
| C2-C3 | σ | C57-O69 | σ* | 3.78 | 1.04 | 0.056 |
| C45-H46 | σ | C17-C41 | σ* | 3.68 | 0.89 | 0.051 |
| C10-S14 | σ | C11-C17 | σ* | 3.27 | 1.13 | 0.055 |
| C96-C106 | σ | C105-C106 | σ* | 3 | 1.22 | 0.054 |
| C26-C78 | σ | C25-C30 | σ* | 2.84 | 1.27 | 0.054 |
| C83-N115 | σ | C25-C80 | σ* | 2.01 | 1.37 | 0.047 |
| C29-C30 | σ | C28-C33 | σ* | 1.96 | 1.21 | 0.044 |
| C97-C99 | σ | C99-H103 | σ* | 1.1 | 1.13 | 0.032 |
| C59-S63 | σ | C59-C60 | σ* | 0.99 | 1.26 | 0.032 |
| C87-H92 | σ | C85-H88 | σ* | 0.81 | 0.94 | 0.025 |
| C86-H90 | σ | C89-H93 | σ* | 0.79 | 0.94 | 0.024 |
| C8-C15 | σ | C11-C17 | σ* | 0.64 | 1.17 | 0.025 |
| C8-C17 | σ | C10-S14 | σ* | 0.56 | 0.82 | 0.019 |
| C6-C59 | σ | C59-S63 | σ* | 0.53 | 0.9 | 0.02 |
| C37-N38 | σ | C33-C36 | σ* | 0.5 | 1.65 | 0.022 |
| C5-C16 | σ | C4-C5 | σ* | 4.22 | 1.26 | 0.065 |
| C9-C10 | σ | C11-C12 | σ* | 4.02 | 1.27 | 0.064 |
| C1- C2 | σ | C2-C3 | σ* | 4 | 1.24 | 0.063 |
| C18-C19 | π | C22-C23 | π* | 34.31 | 0.31 | 0.092 |
| C22-C23 | π | C33-C36 | π* | 28.93 | 0.29 | 0.082 |
| C12-C13 | π | C18-C19 | π* | 26.76 | 0.29 | 0.08 |
| C84-C85 | π | C87-C91 | π* | 23.82 | 0.29 | 0.074 |
| C86-C89 | π | C87-C91 | π* | 21.96 | 0.3 | 0.073 |
| C28-C29 | π | C33-C36 | π* | 20.93 | 0.28 | 0.072 |
| C59-C60 | π | C1-C6 | π* | 19.97 | 0.33 | 0.075 |
| C8-C15 | π | C9-C57 | π* | 16.82 | 0.31 | 0.066 |
| C9-C57 | π | C10-C11 | π* | 15.76 | 0.3 | 0.062 |
| C1-C6 | π | C59-C60 | π* | 12.79 | 0.31 | 0.057 |
| C33-C36 | π | C22-C23 | π* | 8.16 | 0.33 | 0.047 |
| C34-O35 | π | C28-C29 | π* | 3.99 | 0.42 | 0.041 |
| C22-C23 | π | C22-C23 | π* | 3.07 | 0.3 | 0.027 |
| C33-C36 | π | C33-C36 | π* | 1.9 | 0.32 | 0.022 |
| C10-C11 | π | C10-C11 | π* | 0.9 | 0.31 | 0.015 |
| C39-N40 | π | C37-N38 | π* | 0.81 | 0.47 | 0.017 |
| C25 | LP(1) | C26-C27 | π* | 69.94 | 0.14 | 0.106 |
| C25 | LP(1) | C80-C83 | π* | 63.96 | 0.14 | 0.109 |
| O69 | LP(1) | C9-C57 | π* | 6.46 | 0.62 | 0.061 |
| N40 | LP(1) | C36-C39 | σ* | 12.7 | 1.04 | 0.103 |
| N94 | LP(1) | C89-C91 | σ* | 4.27 | 0.85 | 0.059 |
| O119 | LP(1) | C82-N118 | σ* | 3.97 | 1.09 | 0.06 |
| O35 | LP(1) | C23-C34 | σ* | 3.54 | 1.18 | 0.058 |
| S63 | LP(1) | C59-C60 | σ* | 2.78 | 1.24 | 0.053 |
| S63 | LP(1) | C61-C62 | σ* | 2.71 | 1.2 | 0.051 |
| O68 | LP(1) | C1-C6 | σ* | 1.64 | 1.19 | 0.04 |
| S65 | LP(2) | C12-C13 | π* | 29.55 | 0.24 | 0.079 |
| S14 | LP(2) | C10-C11 | π* | 26.91 | 0.28 | 0.078 |
| O120 | LP(2) | N118-O119 | π* | 2.91 | 0.2 | 0.025 |
| O35 | LP(2) | C29-C34 | σ* | 21.35 | 0.75 | 0.115 |
| O116 | LP(2) | N115-O117 | σ* | 20.82 | 0.74 | 0.112 |
| O68 | LP(2) | C1-C2 | σ* | 8.6 | 0.89 | 0.079 |
| O68 | LP(2) | C70-H73 | σ* | 6.82 | 0.7 | 0.062 |
| O68 | LP(2) | C70-H71 | σ* | 5.12 | 0.7 | 0.054 |
| O35 | LP(2) | C12-S20 | σ* | 2.36 | 0.52 | 0.032 |
| S65 | LP(2) | C61-C62 | π* | 24.97 | 0.26 | 0.075 |
| O117 | LP(2) | C80-C83 | π* | 0.68 | 0.33 | 0.014 |
| O117 | LP(3) | N115-O116 | π* | 194.76 | 0.17 | 0.162 |
| O120 | LP(3) | N118-O119 | σ* | 0.99 | 0.72 | 0.027 |
| O120 | LP(3) | N118-O120 | σ* | 0.62 | 0.74 | 0.022 |

**Table S22:** Natural bond orbitals representative values for **PCMD9**.

| **Donor(*i*)** | **Type** | **Acceptor(*j*)** | **Type** | ***E*(2)**  **[*kcal/mol*]** | ***E*(*j*)-*E*(*i*)**  **[*a.u.*]** | **F(*i,j*)**  **[*a.u.*]** |
| --- | --- | --- | --- | --- | --- | --- |
| C62-C64 | σ | C66-C84 | σ* | 4.99 | 1.22 | 0.07 |
| C12-S20 | σ | C13-S14 | σ* | 4.86 | 0.88 | 0.059 |
| C36-C39 | σ | C23-C33 | σ* | 4.29 | 1.25 | 0.065 |
| C85-C87 | σ | C87-C91 | σ* | 4.05 | 1.3 | 0.065 |
| C18-C22 | σ | C18-C19 | σ* | 3.99 | 1.28 | 0.064 |
| C22-C23 | σ | C23-C34 | σ* | 3.79 | 1.21 | 0.061 |
| C23-C34 | σ | C29-C30 | σ* | 3.63 | 1.29 | 0.061 |
| C61-S65 | σ | C66-C84 | σ* | 3.25 | 1.2 | 0.056 |
| C80-C83 | σ | C25-C30 | σ* | 3.01 | 1.29 | 0.056 |
| C82-C115 | σ | C26-C78 | σ* | 2.88 | 1.29 | 0.055 |
| C26-C27 | σ | C78-C82 | σ* | 2.01 | 1.29 | 0.046 |
| C27-C28 | σ | C27-H31 | σ* | 1.95 | 1.16 | 0.042 |
| C25-C30 | σ | C30-H32 | σ* | 1.1 | 1.11 | 0.031 |
| C22-C23 | σ | C29-C34 | σ* | 0.97 | 1.21 | 0.031 |
| O68-C70 | σ | C1-C2 | σ* | 0.82 | 1.37 | 0.03 |
| C111-H114 | σ | C109-C111 | σ* | 0.68 | 1.1 | 0.024 |
| C16-C49 | σ | C49-H51 | σ* | 0.61 | 1.01 | 0.022 |
| C13-S14 | σ | C10-C11 | σ* | 0.52 | 1.24 | 0.023 |
| C10-C11 | σ | C10-S14 | σ* | 0.51 | 0.92 | 0.019 |
| C61-C62 | σ | C6-C59 | σ* | 0.5 | 1.24 | 0.022 |
| C2-C3 | σ | C3-C4 | σ* | 4.23 | 1.23 | 0.065 |
| C25-C30 | σ | C25-C26 | σ* | 4.02 | 1.25 | 0.063 |
| C99-H103 | σ | C98-C101 | σ* | 4 | 1.11 | 0.06 |
| C18-C19 | π | C22-C23 | π* | 34.06 | 0.31 | 0.092 |
| C22-C23 | π | C33-C36 | π* | 28.77 | 0.29 | 0.082 |
| C12-C13 | π | C18-C19 | π* | 26.65 | 0.29 | 0.08 |
| C84-C85 | π | C87-C91 | π* | 23.83 | 0.29 | 0.074 |
| C86-C89 | π | C87-C91 | π* | 21.95 | 0.3 | 0.073 |
| C84-C85 | π | C86-C89 | π* | 20.69 | 0.3 | 0.071 |
| C59-C60 | π | C1-C6 | π* | 19.97 | 0.33 | 0.075 |
| C61-C62 | π | C64-C66 | π* | 16.8 | 0.32 | 0.067 |
| C9-C57 | π | C10-C11 | π* | 15.64 | 0.3 | 0.062 |
| C1-C6 | π | C59-C60 | π* | 12.83 | 0.31 | 0.057 |
| C33-C36 | π | C22-C23 | π* | 8.15 | 0.33 | 0.047 |
| C34-O35 | π | C29-C30 | π* | 4.26 | 0.44 | 0.041 |
| C22-C23 | π | C22-C23 | π* | 3.04 | 0.3 | 0.027 |
| C33-C36 | π | C33-C36 | π* | 1.93 | 0.32 | 0.023 |
| C10-C11 | π | C10-C11 | π* | 0.89 | 0.31 | 0.015 |
| C37-N38 | π | C39-N40 | π* | 0.79 | 0.47 | 0.017 |
| N94 | LP(1) | C105-C106 | π* | 35.4 | 0.31 | 0.096 |
| N94 | LP(1) | C95-C96 | π* | 35.39 | 0.31 | 0.096 |
| N40 | LP(1) | C36-C39 | σ* | 12.69 | 1.04 | 0.103 |
| N94 | LP(1) | C89-C91 | σ* | 4.18 | 0.85 | 0.058 |
| O35 | LP(1) | C23-C34 | σ* | 3.56 | 1.18 | 0.058 |
| S20 | LP(1) | C12-C13 | σ* | 3.35 | 1.18 | 0.056 |
| O68 | LP(1) | C70-H72 | σ* | 2.67 | 0.95 | 0.046 |
| S20 | LP(1) | C18-C19 | σ* | 2.22 | 1.2 | 0.046 |
| O68 | LP(1) | C1-C6 | σ* | 1.69 | 1.19 | 0.04 |
| S20 | LP(2) | C12-C13 | π* | 29.7 | 0.24 | 0.08 |
| S14 | LP(2) | C12-C13 | π* | 22.54 | 0.25 | 0.07 |
| S20 | LP(2) | C18-C19 | π* | 21.38 | 0.26 | 0.067 |
| O35 | LP(2) | C29-C34 | σ* | 21.24 | 0.76 | 0.115 |
| O35 | LP(2) | C23-C34 | σ* | 18.5 | 0.76 | 0.107 |
| O68 | LP(2) | C1-C2 | σ* | 8.61 | 0.89 | 0.079 |
| O68 | LP(2) | C70-H73 | σ* | 6.81 | 0.7 | 0.062 |
| O69 | LP(2) | C74-H77 | σ* | 5.9 | 0.7 | 0.058 |
| O35 | LP(2) | C12-S20 | σ* | 2.41 | 0.52 | 0.032 |
| S14 | LP(2) | C10-C11 | π* | 26.7 | 0.28 | 0.078 |
| S65 | LP(2) | C64-C66 | π* | 23.82 | 0.28 | 0.073 |

**Table S23**: Dipole moment and polarizability with their major contributing tensors (*esu*.) of the studied compounds (**PCMR** and **PCMD1-PCMD9**).

| **Dipole Moment** | | | | |
| --- | --- | --- | --- | --- |
| **Compounds** | ***µ*_x_** | ***µ*_y_** | ***µ*_z_** | ***µ_tot_*** |
| **PCMR** | -5.000×10^-32^ | 1.300×10^-21^ | 3.705×10^-18^ | 3.075×10^-18^ |
| **PCMD1** | -1.035×10^-17^ | 2.362×10^-18^ | -3.808×10^-18^ | 1.128×10^-17^ |
| **PCMD2** | 6.673×10^-18^ | 5.533×10^-18^ | 4.600×10^-18^ | 9.814×10^-18^ |
| **PCMD3** | 6.673×10^-18^ | 5.533×10^-18^ | 4.600×10^-18^ | 9.814×10^-18^ |
| **PCMD4** | 1.024×10^-17^ | 3.504×10^-18^ | 4.465×10^-18^ | 1.171×10^-18^ |
| **PCMD5** | 1.953×10^-17^ | 3.734×10^-18^ | 1.561×10^-18^ | 1.995×10^-17^ |
| **PCMD6** | 1.385×10^-17^ | 1.660×10^-18^ | 2.876×10^-18^ | 1.425×10^-17^ |
| **PCMD7** | 1.037×10^-17^ | 3.902×10^-18^ | 4.301×10^-18^ | 1.188×10^-17^ |
| **PCMD8** | 1.795×10^-17^ | -9.070×10^-18^ | 4.194×10^-18^ | 1.846×10^-17^ |
| **PCMD9** | 1.796×10^-17^ | 1.509×10^-18^ | 1.509×10^-18^ | 1.846×10^-17^ |
| **Linear Hyperpolarizability** | | | | |
| **Compounds** | ***α_xx_*** | ***α_yy_*** | ***α_zz_*** | **<*α*>** |
| **PCMR** | 5.227×10^-22^ | 1.676×10^-22^ | 5.963×10^-23^ | 2.499×10^-22^ |
| **PCMD1** | 4.519×10^-22^ | 1.476×10^-22^ | 9.359×10^-23^ | 2.310×10^-22^ |
| **PCMD2** | 4.868×10^-22^ | 1.865×10^-22^ | 7.514×10^-23^ | 2.495×10^-22^ |
| **PCMD3** | 4.868×10^-22^ | 1.865×10^-22^ | 7.514×10^-23^ | 2.495×10^-22^ |
| **PCMD4** | 5.128×10^-22^ | 1.923×10^-22^ | 7.743×10^-22^ | 2.608×10^-22^ |
| **PCMD5** | 5.344×10^-22^ | 1.945×10^-22^ | 8.467×10^-23^ | 2.712×10^-22^ |
| **PCMD6** | 5.106×10^-22^ | 1.912×10^-22^ | 7.479×10^-23^ | 2.588×10^-22^ |
| **PCMD7** | 5.201×10^-22^ | 1.970×10^-22^ | 8.397×10^-23^ | 2.670×10^-22^ |
| **PCMD8** | 5.342×10^-22^ | 1.932×10^-22^ | 7.938×10^-23^ | 2.686×10^-22^ |
| **PCMD9** | 5.362×10^-22^ | 1.943×10^-22^ | 7.815×10^-23^ | 2.695×10^-22^ |
| **Second Hyperpolarizability** | | | | |
| **Compounds** | ***γ_x_*** | ***γ_y_*** | ***γ_z_*** | ***<γ>*** |
| **PCMR** | 3.91×10^-32^ | 3.979×10^-34^ | 3.091×10^-35^ | 3.958×10^-32^ |
| **PCMD1** | 3.496×10^-32^ | 1.423×10^-34^ | 6.584×10^-35^ | 3.517×10^-32^ |
| **PCMD2** | 4.001×10^-32^ | 3.062×10^-34^ | 6.045×10^-35^ | 4.038×10^-32^ |
| **PCMD3** | 4.001×10^-32^ | 3.062×10^-34^ | 6.045×10^-35^ | 4.038×10^-32^ |
| **PCMD4** | 4.774×10^-32^ | 6.751×10^-34^ | 7.245×10^-35^ | 4.849×10^-32^ |
| **PCMD5** | 6.368×10^-32^ | 1.227×10^-33^ | 3.286×10^-35^ | 6.495×10^-32^ |
| **PCMD6** | 5.184×10^-32^ | 9.187×10^-32^ | 1.276×10^-35^ | 5.276×10^-32^ |
| **PCMD7** | 4.858×10^-32^ | 8.934×10^-34^ | 7.528×10^-35^ | 4.955×10^-32^ |
| **PCMD8** | 6.758×10^-32^ | 9.975×10^-34^ | 9.041×10^-35^ | 6.867×10^-32^ |
| **PCMD9** | 6.536×10^-32^ | 7.777×10^-34^ | 8.453×10^-35^ | 6.622×10^-32^ |

**Table S24**: Frequency dependent Second hyperpolarizability (*esu*) of the studied compounds.

| **Parameters** | **Frequency *ω*** | **PCMR** | **PCMD1** | **PCMD2** | **PCMD3** | **PCMD4** |
| --- | --- | --- | --- | --- | --- | --- |
| *γ*(−ω,ω,0,0) | 0.000 | 9.901×10^-29^ | 2.750×10^-27^ | 3.022×10^-27^ | 3.223×10^-27^ | 3.486×10^-27^ |
|  | 1907.21*nm* | 1.047×10^-28^ | 3.084×10^-27^ | 3.022×10^-27^ | 3.691×10^-27^ | 4.043×10^-27^ |
| *γ*(−2ω,ω,ω,0) | 0.000 | 9.901×10^-29^ | 2.750×10^-27^ | 3.439×10^-27^ | 3.223×10^-27^ | 3.486×10^-27^ |
|  | 1907.21*nm* | 1.742×10^-28^ | 5.729×10^-27^ | 6.575×10^-27^ | 7.256×10^-27^ | 8.247×10^-27^ |
| **Parameters** | **Frequency *ω*** | **PCMD5** | **PCMD6** | **PCMD7** | **PCMD8** | **PCMD9** |
| *γ*(−ω,ω,0,0) | 0.000 | 4.572×10^-27^ | 3.904×10^-27^ | 3.614×10^-27^ | 4.747×10^-27^ | 4.606×10^-27^ |
|  | 1907.21*nm* | 4.572×10^-27^ | 4.540×10^-27^ | 4.199×10^-27^ | 5.653×10^-27^ | 4.606×10^-27^ |
| *γ*(−2ω,ω,ω,0) | 0.000 | 4.572×10^-27^ | 3.904×10^-27^ | 3.614×10^-27^ | 4.747×10^-27^ | 4.606×10^-27^ |
|  | 1907.21*nm* | 1.251×10^-26^ | 9.608×10^-27^ | 8.594×10^-27^ | 1.334×10^-26^ | 4.606×10^-26^ |

**Table S25**: The computed first hyperpolarizability (*β*_tot_) and major contributing tensors (*esu)* of the studied compounds.

| **Hyper**  **Pol.** | **PCMR** | **PCMD1** | **PCMD2** | **PCMD3** | **PCMD4** |
| --- | --- | --- | --- | --- | --- |
| *β_xxx_* | 4.277×10^-32^ | -2.770×10^-27^ | 3.058×10^-27^ | 3.245×10^-27^ | 3.501×10^-27^ |
| *β_xxy_* | 5.027×10^-32^ | 1.546×10^-28^ | 1.467×10^-28^ | 2.329×10^-28^ | 3.134×10^-28^ |
| *β_xyy_* | 5.196×10^-32^ | 2.136×10^-29^ | -4.459×10^-29^ | -3.590×10^-29^ | -3.598×10^-29^ |
| *β_yyy_* | -1.491×10^-33^ | 4.203×10^-30^ | 1.301×10^-29^ | 1.279×10^-29^ | 1.923×10^-29^ |
| *β_xxz_* | 1.036×10^-28^ | 9.284×10^-29^ | 1.190×10^-28^ | 1.281×10^-28^ | 1.431×10^-28^ |
| *β_yyz_* | -2.674×10^-30^ | -1.216×10^-30^ | 5.191×10^-32^ | 4.266×10^-30^ | 1.119×10^-30^ |
| *β_xzz_* | 4.046×10^-33^ | 4.984×10^-30^ | 1.921×10^-30^ | 2.061×10^-30^ | 2.504×10^-30^ |
| *β_yzz_* | -3.164×10^-34^ | -1.256×10^-31^ | -5.330×10^-31^ | -3.040×10^-31^ | -8.860×10^-32^ |
| *β_zzz_* | -2.004×10^-30^ | -5.549×10^-31^ | -1.788×10^-30^ | -1.713×10^-30^ | -1.605×10^-30^ |
| ***β_tot_*** | 9.901×10^-29^ | 2.750×10^-27^ | 3.022×10^-27^ | 3.223×10^-27^ | 3.486×10^-27^ |
| **Hyper**  **Pol.** | **PCMD5** | **PCMD6** | **PCMD7** | **PCMD8** | **PCMD9** |
| *β_xxx_* | 4.495×10^-27^ | 3.863×10^-27^ | 3.577×10^-27^ | 4.686×10^-27^ | 4.583×10^-27^ |
| *β_xxy_* | 5.067×10^-28^ | 4.230×10^-28^ | 3.986×10^-28^ | 3.666×10^-28^ | 2.801×10^-28^ |
| *β_xyy_* | 5.366×10^-29^ | 2.137×10^-29^ | 7.479×10^-30^ | 3.946×10^-29^ | 9.016×10^-30^ |
| *β_yyy_* | -1.157×10^-29^ | -4.773×10^-30^ | -7.967×10^-31^ | 2.223×10^-29^ | -1.246×10^-29^ |
| *β_xxz_* | 6.781×10^-29^ | 3.994×10^-29^ | 1.460×10^-28^ | 1.799×10^-28^ | 1.699×10^-28^ |
| *β_yyz_* | 2.602×10^-31^ | 1.522×10^-32^ | -4.430×10^-31^ | -3.261×10^-30^ | -1.832×10^-30^ |
| *β_xzz_* | -3.527×10^-30^ | -2.369×10^-30^ | 4.403×10^-30^ | 5.610×10^-30^ | 3.924×10^-30^ |
| *β_yzz_* | 4.609×10^-32^ | 1.790×10^-31^ | -2.071×10^-31^ | -4.237×10^-31^ | -2.940×10^-31^ |
| *β_zzz_* | -1.649×10^-30^ | -1.687×10^-30^ | -1.518×10^-30^ | -1.565×10^-30^ | -1.567×10^-30^ |
| ***β_tot_*** | 4.572×10^-27^ | 3.904×10^-27^ | 3.614×10^-27^ | 4.747×10^-27^ | 4.606×10^-27^ |

**Table S26**: Frequency dependent first hyperpolarizability (*esu*) of the studied compounds.

|  | **Parameters** | **Frequency *ω*** | **PCMR** | **PCMD1** | **PCMD2** | **PCMD3** | **PCMD4** |
| --- | --- | --- | --- | --- | --- | --- | --- |
| Static | β (−ω;ω,0,) | 0.000 | 3.958×10^-32^ | 3.517×10^-32^ | 4.038×10^-32^ | 4.355×10^-32^ | 4.849×10^-32^ |
|  | β (-2ω;ω,ω) | 0.000 | 3.958×10^-32^ | 3.517×10^-32^ | 4.038×10^-32^ | 4.355×10^-32^ | 4.849×10^-32^ |
| Specific | β (−ω;ω,0) | 1907.21nm | 4.643×10^-32^ | 4.297×10^-32^ | 5.000×10^-32^ | 5.462×10^-32^ | 6.187×10^-32^ |
|  | (−2ω;ω,ω) | 1907.21nm | 9.977×10^-32^ | 1.093×10^-32^ | 1.317×10^-31^ | 1.529×10^-32^ | 1.856×10^-31^ |
|  | **Parameters** | **Frequency ω** | **PCMD5** | **PCMD6** | **PCMD7** | **PCMD8** | **PCMD9** |
| Static | β (−ω;ω,0,) | 0.000 | 6.495×10^-32^ | 5.276×10^-32^ | 6.369×10^-32^ | 6.867×10-^32^ | 6.622×10^-32^ |
|  | β (-2ω;ω,ω) | 0.000 | 6.495×10^-32^ | 5.276×10^-32^ | 4.955×10^-32^ | 6.867×10^-32^ | 6.622×10^-32^ |
| Specific | β (−ω;ω,0) | 1907.21nm | 8.790×10^-32^ | 6.858×10^-32^ | 6.858×10^-32^ | 9.384×10^-32^ | 8.956×10^-32^ |
|  | β(−2ω;ω,ω) | 1907.21nm | 3.668×10^-31^ | 2.298×10^-31^ | 1.952×10^-32^ | 4.242×10^-31^ | 3.759×10^-32^ |

| **Compounds** | **Structure** |
| --- | --- |
| **PCMR** |  |
| **PCMD1** |  |
| **PCMD2** |  |
| **PCMD3** |  |
| **PCMD4** |  |
| **PCMD5** |  |
| **PCMD6** |  |
| **PCMD7** |  |
| **PCMD8** |  |
| **PCMD9** |  |

**Figure S1:** ChemDraw structures of the designed compounds **R** and **PCMD1**-**PCMD9** along with their IUPAC name.

| **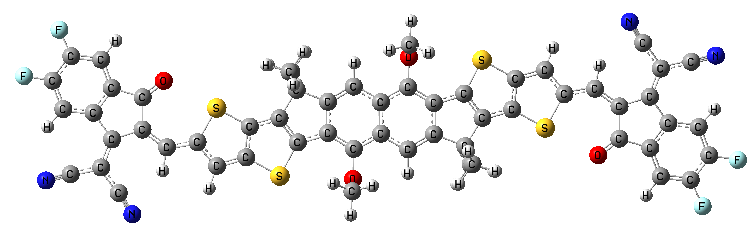** | **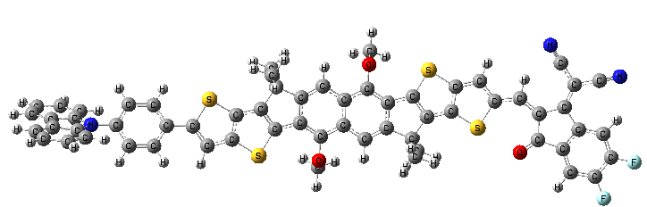** |
| --- | --- |
| **PCMR** | **PCMD1** |
| **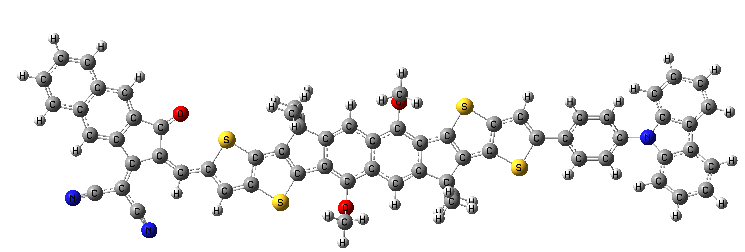** | **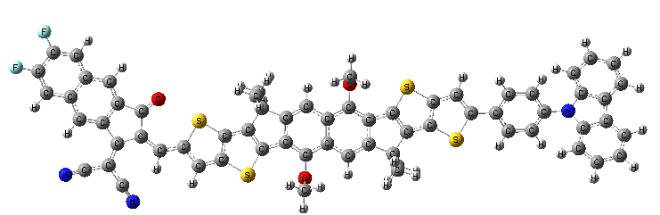** |
| **PCMD2** | **PCMD3** |
| **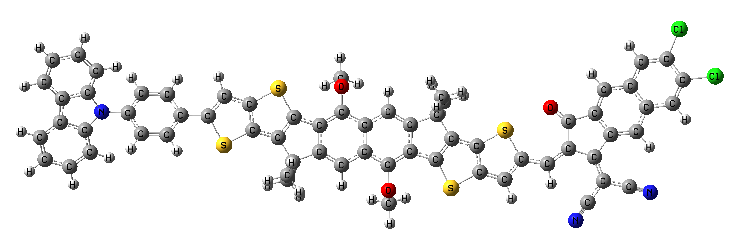** | **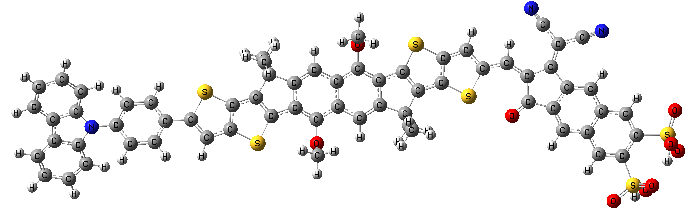** |
| **PCMD4** | **PCMD5** |
| **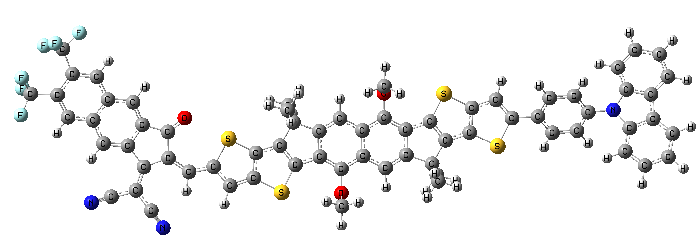** | **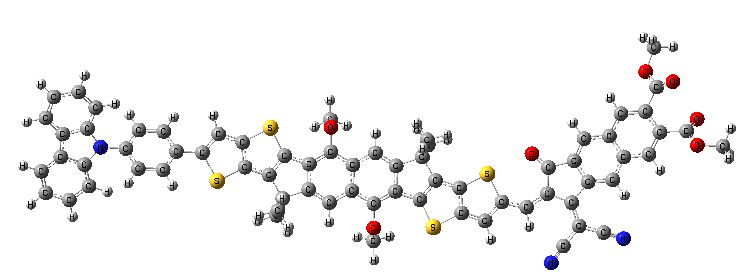** |
| **PCMD6** | **PCMD7** |
| **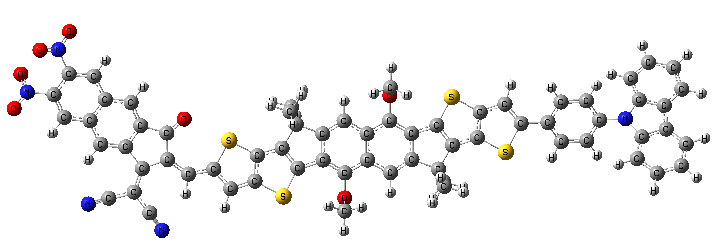** | **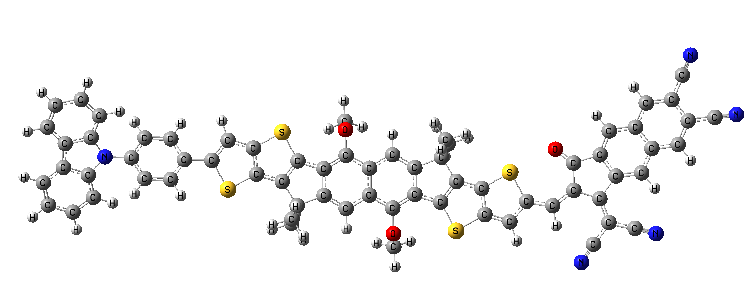** |
| **PCMD8** | **PCMD9** |

**Figure S2:** **PCMR** and **PCMD1**-**D9** optimized structures drawn *via* GaussView 6.0 program.
